# Exploring blood metabolites and thyroid disorders: a bidirectional mendelian randomization study

**DOI:** 10.3389/fendo.2023.1270336

**Published:** 2023-10-09

**Authors:** Xuan Zhang, Jiating Zhou, Zilan Xie, Xi Li, Jiaqing Hu, Hengzheng He, Zhi Li

**Affiliations:** ^1^ Department of Clinical Pharmacology, Xiangya Hospital, Central South University, Changsha, Hunan, China; ^2^ Institute of Clinical Pharmacology, Central South University, Changsha, Hunan, China; ^3^ Engineering Research Center of Applied Technology of Pharmacogenomics, Ministry of Education, Changsha, Hunan, China; ^4^ National Clinical Research Center for Geriatric Disorders, Xiangya Hospital, Changsha, Hunan, China; ^5^ Department of General Surgery, The Second People’s Hospital of Hunan, Changsha, Hunan, China; ^6^ Department of Emergency Medicine, Trauma Center, The Second People’s Hospital of Hunan, Changsha, Hunan, China

**Keywords:** metabolites, Mendelian randomization, bidirectional, thyroid cancer, autoimmune thyroid disease

## Abstract

**Background:**

Human blood metabolites have demonstrated close associations with thyroid disorders in observational studies. However, it’s essential to determine whether these correlations imply causation. Mendelian Randomization (MR) offers a promising approach to investigate these patterns.

**Aims:**

The primary aim of our investigation is to establish causality between blood metabolites and three thyroid disorders: TC, GD, and HT.

**Methods:**

We employed a two-sample bidirectional MR analysis approach to assess the relationships between 452 blood metabolites and the three aforementioned thyroid disorders. Causal links were estimated using the IVW method, with sensitivity analyses conducted via MR-Egger, Weighted Median, and MR-PRESSO. We assessed potential heterogeneity and pleiotropy using MR-Egger intercept and Cochran’s Q statistic. Additionally, we conducted pathway analysis to identify potential metabolic pathways.

**Results:**

We found 46 metabolites that showed suggestive associations with thyroid disease risk, especially Aspartate (OR_IVW_=7.41; 95%CI: 1.51-36.27; P_IVW_=0.013) and C-glycosyltryptophan (OR_IVW_=0.04; 95%CI: 0.00–0.29; P_IVW_=0.001) impacted TC, Kynurenine (OR_IVW_=2.69; 95%CI: 1.08–6.66; P_IVW_=0.032) and 4-androsten-3beta,17beta-diol disulfate 2 (OR_IVW_=0.78; 95%CI: 0.48–0.91; P_IVW_=0.024) significantly impacted GD, and Alpha-ketoglutarate (OR_IVW_=46.89; 95%CI: 4.65–473.28; P_IVW_=0.001) and X-14189–leucylalanine (OR_IVW_=0.31; 95%CI: 0.15–0.64 P_IVW_=0.001) significantly impacted HT. We also detected 23 metabolites influenced by TC and GD. Multiple metabolic pathways have been found to be involved in thyroid disease.

**Conclusion:**

Our MR findings suggest that the identified metabolites and pathways can serve as biomarkers for clinical thyroid disorder screening and prevention, while also providing new insights for future mechanistic exploration and drug target selection.

## Introduction

Thyroid diseases encompass a variety of disorders involving the thyroid gland, including thyroid cancer (TC), Graves’ disease (GD), and Hashimoto’s thyroiditis (HT). Thyroid cancer is the most prevalent malignant thyroid tumor (1), while Graves’ disease and Hashimoto’s thyroiditis are common organ-specific disorders (2), with Th17 cells playing a foundational role in these conditions (3). These three diseases notably affect both incidence rates and affected individuals’ quality of life. Thyroid hormones play a pivotal role in regulating numerous metabolic processes within the body, thereby influencing the overall metabolic status of individuals. The association between metabolic abnormalities and the incidence of thyroid disorders is of significance, as it directly impacts the quality of life for affected patients. Studying the relationship between metabolic issues and these thyroid diseases has garnered significant attention.

Observational research has shown a strong link between specific blood metabolites and the development and advancement of thyroid diseases. Regarding thyroid cancer, prior studies have established a correlation between blood thyroid hormone levels and thyroid cancer occurrence (4).. Additionally, some blood metabolites such as vitamin D, lipid metabolites, and carbohydrate metabolites have shown abnormal changes associated with thyroid cancer (5, 6). There is relatively less research on Graves’ disease and Hashimoto’s thyroiditis. Murdaca et al. discovered a certain correlation between the development of autoimmune thyroid diseases and factors such as vitamin D and the microbiome (7). In another study, there was a noticeable association between thyroid hormone levels in the blood of Graves’ disease patients and lipid metabolites (8, 9).. In studies on Hashimoto’s thyroiditis, the focus has been on the interaction between thyroid antibodies and immune-related metabolites in the blood (10, 11). Although prior research has explored the link between human blood metabolites and thyroid diseases, there remains a need for more extensive and systematic studies to fully determine the causal relationship between these diseases and metabolites.

Mendelian randomization (MR), an analytical approach, is vital for exploring the causal link between metabolites and thyroid diseases. Given the absence of viable randomized controlled trials or the practicality of initiating new ones, the MR method has emerged as a crucial alternative for evaluating the causal connection between metabolites and disease risk. Specifically, in the MR approach, single nucleotide polymorphisms (SNPs) are employed as instrumental variables (IVs) to represent the specific phenotype (12). The MR approach’s main strength is utilizing the naturally occurring genetic variations allocated randomly during fertilization. This mimics a randomized controlled trial, effectively minimizing biases from confounding factors like gender and age in causal analysis (13). Furthermore, genotype establishment happens before the onset of disease and remains mostly uninfluenced by disease advancement, thereby enabling the effective evaluation of the causal links between metabolites and thyroid diseases (14, 15).

To identify potential candidate metabolites associated with the etiology of thyroid diseases in a more exploratory manner, we performed bidirectional two-sample MR analysis using the latest and extensive genome-wide association study (GWAS) summary data available (16). The purpose of this analysis was to explore bidirectional causal associations between human blood metabolites and both TC and AITD (including GD and HT). This research approach enhances comprehension of thyroid disease pathogenesis and metabolic pathways, while also furnishing dependable evidence for devising viable strategies in thyroid screening and prevention within clinical settings.

## Materials and methods

### MR design

**Figure 1:**
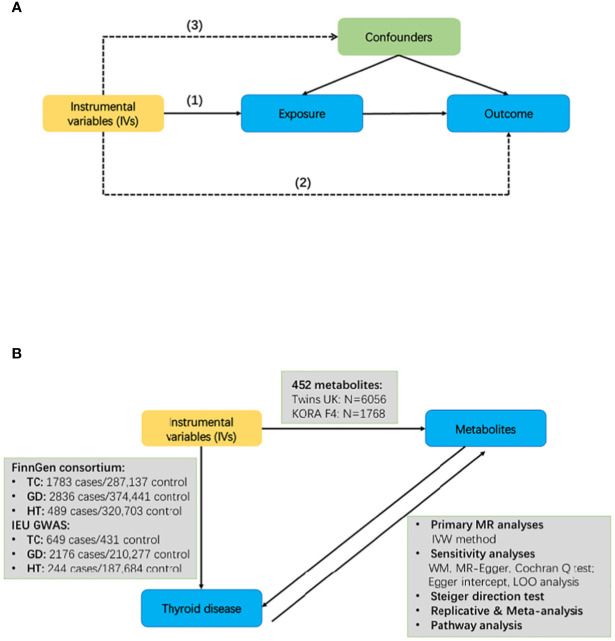
Schematic overview of the study design. **(A)** Mendelian randomization [MR] illustration. There are three principal assumptions in MR design: (1) IVs must be strongly correlated with exposure factors; (2) IVs was associated with outcomes only by exposure; (3) IVs cannot be associated with any confounding factors. **(B)** Bidirectional MR study of metabolites and thyroid disease TC, Thyroid cancer; GD, Graves' disease; HT, Hashimoto thyroiditis; IVW, Inverse variance weighted; WM, weighted median; LOO, leave-one-out.

We employed a bidirectional two-sample MR to evaluate the causal link between 452 human blood metabolites and TC and AITD. **Figure 1** provides a schematic overview of the study design and data sources. GWAS summary statistics were obtained to extract prominent single nucleotide polymorphisms [SNPs] serving as genetic instrumental variables for 452 human blood metabolites, TC, and AITD. Initially, we designated the 452 blood metabolites as the exposure and TC, along with AITD, as the outcomes to ascertain their potential roles in either inhibiting or fostering the onset of TC and AITD. TC and AITD were then used as the exposure and blood metabolites as the outcome to investigated changes in metabolites after the occurrence of disease. The summary-level data utilized in this study can be downloaded and has been obtained with ethical approval from the respective institutions overseeing each GWAS involved.

Analyses were executed using R statistical software (version 4.2.3). MR analyses were conducted with the R-based tools “TwoSampleMR” and “MR-PRESSO,” and the meta-analysis employed the “meta” package (13).

### Data sources

The data for this study were sourced from two GWASs: one focusing on metabolites and the other on thyroid disease. The dataset comprising 452 metabolites for the GWAS was amalgamated from a genome-wide association scan and high-throughput metabolic analysis study conducted by Shin et al. This study enrolled 7824 participants mainly from two European population cohorts, with screening conducted for approximately 21,000 SNPs (12, 17). The participant pool comprised 1,768 individuals from the KORA F4 study in Germany and 6,056 participants from the Twin study. Both studies received approval from local ethics committees, and all participants provided informed consent. This represents the most extensive investigation to date on the genetic impact on human serum metabolism. Following rigorous quality control, a total of 452 metabolites underwent genetic analysis in both cohorts. These encompassed 275 recognized metabolites and 177 unidentified ones. As detailed in the KEGG database, the 275 established metabolites are categorized into eight major metabolic groups: amino acids, carbohydrates, cofactors and vitamins, energy, lipids, nucleotides, peptides, and allogenic metabolism (18).

The GWAS summaries for TC, GD, and HT were acquired from the FINNGEN consortium (r9.finngen.fi). The FINNGEN consortium is a Finnish national meta-analysis of GWAS that analyzed 13 cohorts and biobanks. The GWAS summary data for TC included 288,920 samples (1783 individuals with TC and 287,137 without) with a dataset of 18,707,521 SNPS. The summary data for GD included 377,277 samples (2836 GD cases and 374,441 controls), encompassing a dataset of 18,709,621 SNPs. The GWAS summary data for HT contained 321,192 samples (489 Hashimoto’s thyroiditis and 320,703 controls) with a dataset of 18,708,398 SNPS. **Supplementary Table** **S2** shows the detailed information.

### Selection criteria for genetic variants

In the process of selecting instrumental variables (IVs) to signify potential exposure-outcome links, varied thresholds were established according to exposure variations. First, 452 metabolites were designated as the exposure. In this case, SNP with an association threshold of P<1×10^-5^ were extracted (19), mainly for MR Analysis, while a linkage disequilibrium parameter (r^2^) of <0.001 was set in the 10,000 kb window of the European 1000 Genome reference Panel to obtain top-level independent SNP. When the chosen SNPs from the exposure dataset were not present in the outcome dataset, proxy SNPs displaying substantial association with the selected variants (R^2^>0.8) were employed instead. Secondly, When TC and AITD were designated as exposures, IV significance adhered to the genome-wide statistical significance threshold (p<5×10^-8^) (20). Moreover, a linkage disequilibrium threshold of 0.001 and a clumping window of 10,000 kb were established. MR Steiger filters were employed to exclude SNPs with incorrect causal directions. Furthermore, R2 and F-statistic of the IVs were computed to identify potential weak IV bias (21). SNPs with F<10 were deemed weak instruments and removed to guarantee ample variance from all SNPs for the respective exposed group (22). The design formula is shown in **Table S1**, adhering to the common recommendation of using a threshold of F>10 for MR analysis. Then, exposure SNPs were isolated from outcome data, while excluding SNPs associated with the outcome. Following this, alignment of allele information for exposure and outcome SNPs was performed during data harmonization. Lastly, metabolites with a minimum of 2 SNPs were retained for MR analysis.

### Primary analysis

This study employed bidirectional two-sample MR to estimate causal effects between metabolites and thyroid disease using inverse variance weighting (IVW) (23). IVW is a prominent method frequently employed in MR studies, effectively aggregating Wald ratios for each SNP to yield a consolidated estimate. The random effects model of IVW is utilized in cases of heterogeneity, while the fixed effects model is applied in its absence. We further applied a multiple-testing corrected threshold of P<0.05/275 (where 275 represents the count of known metabolites) using the Bonferroni correction to elucidate statistical significance (24). P-values ranging from 1.82×10^-4^ to 0.05 were regarded as possible associations.

### Sensitivity analysis and direction validation

To validate that IV influences the outcome solely through the exposure, and to improve the robustness of the findings, sensitivity analysis is also required. Therefore, Different approaches such as weighted median, MR-Egger, MR-PRESSO, and leave-one-out were employed in sensitivity analysis to validate the stability of the significant estimates found (IVW P<0.05). Among them, The weighted median method enhances causal effect detection and reduces type I errors. MR-Egger identifies IV assumption violations and offers unaffected effect estimates. Concurrently, we examined the presence of horizontal pleiotropy using MR-Egger regression and the MR-PRESSO Global test (25). Horizontal pleiotropy suggests that IVs might be linked to outcomes through non-causal pathways, possibly yielding false positives (p<0.05). The leave-one-out method assesses whether individual SNPs influence the results (26). Furthermore, the Cochran Q test was employed to identify heterogeneity, with a resulting Cochran-Q derived p-value below 0.05 indicating its presence (27). Lastly, the MR Steiger directionality test was performed to validate if our findings aligned with our hypothesis (28).

### Replication analysis

We replicated the IVW analysis using GWAS data for TC, GD, and HT from the IEU Open database (29), aiming to cross-validate the reliability of our findings. (**Table S2**)

### Metabolic pathway analysis

The chosen metabolite metabolic pathways were investigated using the web-based tool Metaconflict 5.0 (30) https://www.metaboanalyst.ca/. MetaboAnalyst 5.0 serves as a user-friendly online tool for efficient metabolomics data analysis. In this study, only metabolites exceeding the advised threshold were analyzed for metabolic pathways (P_IVW_<0.05).

## Results

### Causal effects of the human blood metabolites on TC, GD, and HT

The metabolite instrumental variables ranged from 4 to 150, with a median count of 12. SNP F-statistics exceeded 10, indicating absence of weak instrumental variables. (**Table S3**). Based on these instrumental variables we performed the IVW MR Analysis for each pair of metabolites and TC and AITD. A total of 46 metabolites (P_IVW_<0.05) with significant associations were identified, including 22 known metabolites and 24 unknown metabolites. Among these, 10 (**Figures S1-S4**), 5(**Figures S5-S8**) and 7(**Figures S9-S12**) associations of known metabolites (**Table 1**) and 9, 4 and 11 unknown metabolite associations were identified with increased risk of TC, GD, and HT, respectively. Genetic variants elucidating metabolite associations with three thyroid diseases were presented in **Tables S5-S7**. **Supplementary Figures** displayed scatter plots, funnel plots, and leave-one-out sensitivity analysis.

**Table 1:** Effect of metabolites on thyroid disease.

Outcome	Exposure	N	IVW		Heterogeneity		Pleiotropy		Steiger direction	
			OR(95%CI)	P	IVW Q	P	Intercept	P	correct_causal_direction	steiger_pval
**Thyroid cancer**	Phenylalanine	5	258.64(2.30-29102.41)	0.021	1.260	0.868	-0.0125196	0.802	TRUE	2.923E-38
**Thyroid cancer**	Aspartate	4	7.41(1.51-36.27)	0.013	2.712	0.438	-0.0740803	0.364	TRUE	5.46977E-27
**Thyroid cancer**	C-glycosyltryptophan*	20	0.04(0.00-0.29)	0.002	11.796	0.894	0.0062277	0.854	TRUE	2.5161E-153
**Thyroid cancer**	Carnitine	150	0.26(0.08-0.81)	0.020	152.504	0.405	-0.013534	0.144	TRUE	0
**Thyroid cancer**	1-linoleoylglycerol (1-monolinolein)	13	0.54(0.30-0.98)	0.044	13.888	0.308	-0.0328429	0.363	TRUE	9.0806E-80
**Thyroid cancer**	Stearoylcarnitine	6	5.77(1.11-29.90)	0.037	1.651	0.895	0.0495728	0.639	TRUE	2.86814E-32
**Thyroid cancer**	Gamma-glutamylglutamine	19	3.54(1.15-10.90)	0.028	14.826	0.674	-0.0284283	0.115	TRUE	1.0729E-135
**Thyroid cancer**	Gamma-glutamylleucine	32	0.28(0.08-0.96)	0.043	31.096	0.461	-0.0184384	0.221	TRUE	3.8975E-197
**Thyroid cancer**	Uridine	18	0.06(0.01-0.43)	0.006	12.667	0.758	0.0165484	0.641	TRUE	3.49E-118
**Thyroid cancer**	Myristoleate (14:1n5)	14	0.35(0.17-0.72)	0.005	8.587	0.803	-0.0292185	0.206	TRUE	9.0905E-106
**Graves’ disease**	Kynurenine	32	2.69(1.08-6.66)	0.033	26.860	0.679	0.0030935	0.823	TRUE	3.3375E-260
**Graves’ disease**	Taurochenodeoxycholate	11	0.70(0.50-0.98)	0.040	6.428	0.778	-0.0282023	0.134	TRUE	3.8636E-99
**Graves’ disease**	4-androsten-3beta,17beta-diol disulfate 2*	18	0.48(0.25-0.91)	0.024	21.012	0.226	0.0277824	0.346	TRUE	5.1913E-104
**Graves’ disease**	Phenylalanylphenylalanine	4	3.66(1.10-12.19)	0.035	0.899	0.826	0.0211657	0.685	TRUE	5.87742E-25
**Graves’ disease**	Phosphate	4	0.09(0.01-0.86)	0.037	2.511	0.473	-0.0348529	0.394	TRUE	4.17112E-28
**Hashimoto thyroiditis**	Kynurenine	32	13.00(1.15-147.44)	0.038	38.838	0.157	0.012907	0.732	TRUE	1.6446E-257
**Hashimoto thyroiditis**	3-methylhistidine	8	0.29(0.09-0.91)	0.034	9.720	0.205	-0.0195768	0.862	TRUE	1.55711E-71
**Hashimoto thyroiditis**	Phenol sulfate	10	0.21(0.05-0.85)	0.029	8.984	0.439	-0.0009382	0.993	TRUE	1.41162E-77
**Hashimoto thyroiditis**	2-palmitoylglycerophosphocholine*	17	9.83(1.19-81.37)	0.034	17.157	0.376	-0.0139517	0.679	TRUE	4.571E-102
**Hashimoto thyroiditis**	X-14189–leucylalanine	11	0.31(0.15-0.64)	0.002	7.408	0.686	-0.0208127	0.644	TRUE	6.48194E-84
**Hashimoto thyroiditis**	Gamma-tocopherol	11	0.21(0.06-0.71)	0.012	7.813	0.647	-0.019332	0.711	TRUE	6.5734E-104
**Hashimoto thyroiditis**	Alpha-ketoglutarate	11	46.89(4.65-473.28)	0.001	11.052	0.354	-0.0087717	0.957	TRUE	1.6218E-113

Specifically, Aspartate (OR_IVW_=7.41; 95%CI: 1.51-36.27; P_IVW_=0.013), Kynurenine (OR_IVW_=2.69; 95%CI: 1.08–6.66; P_IVW_=0.032), Alpha-ketoglutarate (OR_IVW_=46.89; 95%CI: 4.65–473.28; P_IVW_=0.001) were the most notably risky metabolites for thyroid cancer, Graves’ disease and Hashimoto’s thyroiditis. On the contrary, C-glycosyltryptophan (OR_IVW_=0.04; 95%CI: 0.00–0.29; P_IVW_=0.001), 4-androsten-3beta,17beta-diol disulfate 2 (OR_IVW_=0.78; 95%CI: 0.48–0.91; P_IVW_=0.024), X-14189–leucylalanine (OR_IVW_=0.31; 95%CI: 0.15–0.64; P_IVW_=0.001) were factors with highest protective value for TC, GD and HT (**Table 2**). **Table 1** shows the characteristics of all significant pathogenic relationships between known metabolites of different types of thyroid disease. Subsequently, Bonferroni correction was employed to identify causal association characteristics (P<1.82×10^-4^). The results showed that the P-values of the selected metabolites were all between 1.82×10^-4^ and 0.05, indicating that some metabolites were potentially associated with TC and ATD, but there was no clear causal relationship. Additional research is necessary to validate their relationship.

**Table 2:** The most detrimental and protective factors for the three thyroid diseases.

Trait	Exposure	IVW		MR-Egger		Weighted median	
		OR (95% CI)	P value	OR (95% CI)	P value	OR (95% CI)	P value
**Thyroid cancer**	Aspartate	7.41(1.51-36.27)	0.013	89.24(1.01-7882.92)	0.188	6.67(0.80-55.52)	0.079
**Thyroid cancer**	C-glycosyltryptophan	0.04(0.00-0.29)	0.001	0.02(0.00-14.33)	0.261	0.04(0.00-0.88)	0.041
**Graves’ disease**	Kynurenine	2.69(1.08-6.66)	0.033	2.10(0.21-21.28)	0.533	1.05(0.26-4.19)	0.945
**Graves’ disease**	4-androsten-3beta,17beta-diol disulfate 2	0.48(0.25-0.91)	0.024	0.17(0.02-1.53)	0.133	0.40(0.17-0.94)	0.036
**Hashimoto thyroiditis**	Alpha-ketoglutarate	46.89(4.65-473.28)	0.001	74.02(0.00-875286845)	0.617	11.17(0.46-268.59)	0.137
**Hashimoto thyroiditis**	X-14189–leucylalanine	0.31(0.15-0.64)	0.001	0.40(0.11-1.50)	0.209	0.34(0.13-0.92)	0.033

IVW, inverse variance weighted.

Because IVW methods are susceptible to weak instrumental bias, sensitivity analyses were conducted to ensure the robustness of the causal assessment. The MR-Egger, weighted mode, simple mode, and weighted median approaches yield consistent causal estimates in terms of both strength and direction. Directed pleiotropy was evaluated using MR-Egger intercept and Mendelian randomized pleiotropy residuals (MR-PRESSO), indicating no significant findings (all P > 0.05). Heterogeneity was assessed through IVW test and Cochran Q statistic in MR-Egger regression. There was also no indication of significant heterogeneity between instrument SNP effects. We further performed Steiger tests to verify the direction of effects from metabolites to TC, GD and HT. The Steiger P-value suggests no reverse causality bias in the identified causality (**Table 1**).

### Causal effects of TC, GD, and HT on human blood metabolites

To assess any reverse causal effects, we performed a reverse MR Analysis using TC, GD and HT as exposures and 452 blood metabolites as results. Among them, the number of instrumental variables for each disease ranged from 4 to 11, and all IVs had F-statistics considerably > 10. (**Table S4**) Results suggested a causal effect of TC on Glutamine (OR_IVW_=1.00, 95%CI=0.99–1.00, P_IVW_ = 0.016), Ornithine (OR_IVW_=0.99, 95%CI=0.98–1.00, P_IVW_ = 0.036), 5-oxoproline (OR_IVW_=0.99, 95%CI = 0.99–1.00, P_IVW_= 0.037), X-12095–N1-methyl-3-pyridone-4-carboxamide (OR_IVW_=1.01, 95%CI = 1.00–1.02, P_IVW_= 0.021), and Taurolithocholate 3-sulfate (OR_IVW_=0.98, 95%CI = 0.96–1.00, P_IVW_ = 0.009) (**Figures S13-S16**). GD has potential causal relationship with 13 metabolites such as Glutamine (OR_IVW_=1.00, 95% CI = 0.99–1.00, P_IVW_= 0.027) (**Table 3**; **Figures S17-S20**). The Q statistic of Cochran showed no heterogeneity (P>0.05). An MR-Egger intercept revealed no horizontal pleiotropy (P>0.05), while Cochran’s Q statistics indicated no heterogeneity (P>0.05). By conducting MR Steiger’s directionality test, SNPs with wrong directionality were excluded, and the results showed that HT had no causal relationship with metabolites. **Tables S8-S9** provide the genetic variants responsible for the association between the three thyroid diseases and identified metabolites.

**Table 3:** Effect of thyroid disease on metabolites.

Outcome	Exposure	N	IVW		Heterogeneity		Pleiotropy		Steiger direction	
			OR(95%CI)	P	IVW Q	P	Intercept	P	correct_causal_direction	steiger_pval
**Glutamine**	Thyroid cancer	4	1.00(0.99-1.00)	0.017	0.469	0.926	-0.0007116	0.745	TRUE	0.323031496
**Ornithine**	Thyroid cancer	4	0.99(0.98-1.00)	0.036	1.687	0.640	0.0046533	0.375	TRUE	0.605736847
**5-oxoproline**	Thyroid cancer	4	0.99(0.99-1.00)	0.037	3.150	0.369	-0.0034355	0.315	TRUE	0.780286918
**X-12095–N1-methyl-3-pyridone-4-carboxamide**	Thyroid cancer	4	1.01(1.00-1.02)	0.022	0.171	0.982	0.0011305	0.835	TRUE	0.409721711
**Taurolithocholate 3-sulfate**	Thyroid cancer	4	0.98(0.96-1.00)	0.009	0.593	0.898	0.0058003	0.637	TRUE	0.777404811
**Glutamine**	Graves’ disease	11	1.00(0.99-1.00)	0.028	5.464	0.858	-0.000132	0.896	TRUE	0.480345297
**Stearate (18:0)**	Graves’ disease	11	1.00(1.00-1.01)	0.049	6.283	0.791	0.0019111	0.273	TRUE	0.176588404
**2-hydroxystearate**	Graves’ disease	11	1.01(1.00-1.01)	0.047	7.492	0.678	0.0021971	0.272	TRUE	0.27966876
**1-oleoylglycerol (1-monoolein)**	Graves’ disease	11	1.02(1.00-1.03)	0.008	7.539	0.674	0.0009299	0.832	TRUE	0.946667749
**2-hydroxyisobutyrate**	Graves’ disease	6	1.00(0.99-1.01)	0.041	2.819	0.728	0.008937	0.268	TRUE	0.916619542
**3-(4-hydroxyphenyl)lactate**	Graves’ disease	8	1.00(0.99-1.00)	0.043	5.290	0.382	-0.0063934	0.437	TRUE	0.379018338
**Scyllo-inositol**	Graves’ disease	9	1.01(1.00-1.02)	0.046	4.350	0.825	-0.00558	0.149	TRUE	0.452788967
1-arachidonoylglycerophosphocholine*	Graves’ disease	11	1.01(1.00-1.02)	0.007	6.976	0.728	-0.0007676	0.760	TRUE	0.598643183
1-palmitoleoylglycerophosphocholine*	Graves’ disease	11	1.01(1.00-1.02)	0.003	5.263	0.873	-0.0014652	0.578	TRUE	0.523981309
1-docosahexaenoylglycerophosphocholine*	Graves’ disease	11	1.01(1.00-1.02)	0.014	4.268	0.934	-0.0015551	0.592	TRUE	0.238630271
**N2,N2-dimethylguanosine**	Graves’ disease	6	0.99(0.99-1.00)	0.049	2.120	0.832	0.00183	0.371	TRUE	0.536838388
**Glutaroyl carnitine**	Graves’ disease	11	1.01(1.00-1.01)	0.044	10.978	0.359	-0.0020548	0.335	TRUE	0.290557508
**1-myristoylglycerophosphocholine**	Graves’ disease	11	1.01(1.00-1.02)	0.029	3.766	0.957	-0.0009854	0.737	TRUE	0.144661835

### Replication analysis

**Figure 2:**
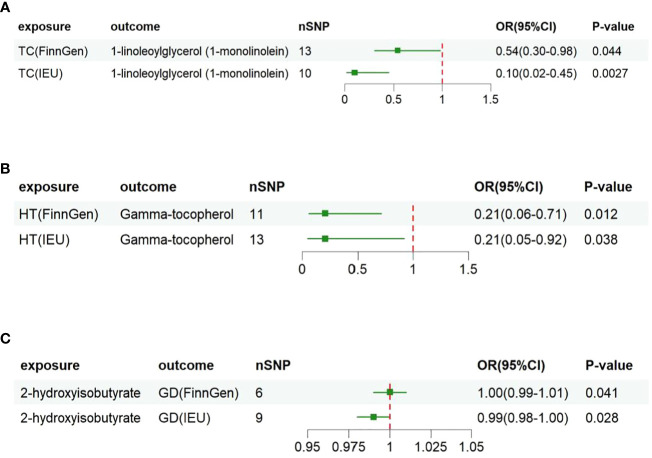
Associations of genetically predicted three metabolites with risk of thyroid disorders using IVW method with different GWAS datasets. OR, odds ratio; CI, confidence. **(A)** 1-linoleoylglycerol (1-monolinolein) shows potential causal association in two different thyroid cancer datasets (FinnGen Consortium and IEU Open GWAS); **(B)** Gamma-tocopherol shows potential causal association in two different Hashimoto's thyroiditis datasets; **(C)** Potential causal effects of 2-hydroxyisobutyrate in two different Graves' disease datasets.

To further validate our findings, we conducted replication analyses using GWAS data from IEU for TC, GD, and HT. The results demonstrated that the three metabolites, 1-linoleoylglycerol(1-monolinolein), Gamma-tocopherol, and 2-hydroxyisobutyrate, all passed the replication test based on IVW analysis. Subsequent combined analyses of FINNGEN and IEU Open GWAS datasets further confirmed that higher levels of 1-linoleoylglycerol (1-monolinolein) and Gamma-tocopherol were associated with reduced risk of TC and HT in individuals with genetic susceptibility. Additionally, fluctuations in 2-hydroxyisobutyrate levels were observed following the occurrence of GD (**Figure 2**).

### Metabolic pathway analysis

**Figure 3:**
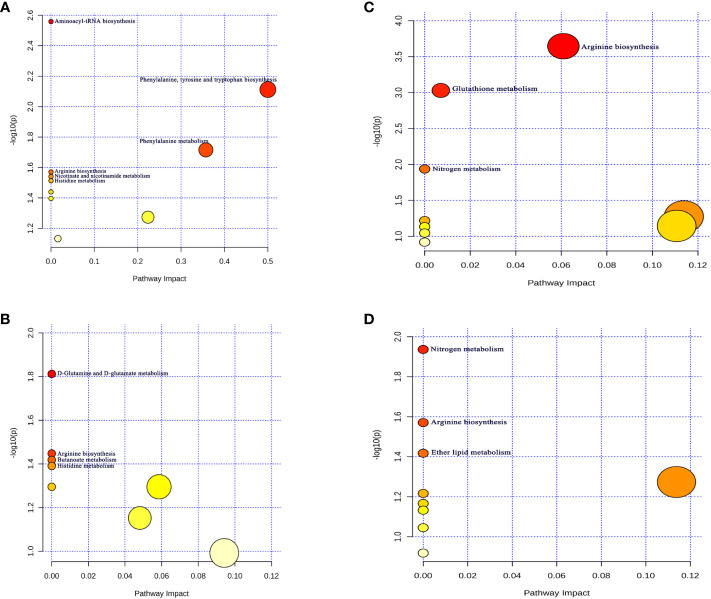
Potential metabolic pathways associated with thyroid disease. **(A)** Potential metabolic pathways involved in the pathogenesis of thyroid cancer in positive MR Analysis; **(B)** Hashimoto thyroiditis (The positive MR Analysis); related to **Additional File 2** **Table S7** **(C)** Thyroid cancer (The reverse MR Analysis); **(D)** Graves' disease (The reverse MR Analysis). related to **Additional File 2** **Table S8** Pathway analysis based on "Kyoto Encyclopedia of Genes and Genomes" (KEGG). The color and size of each circle is based on p-values (yellow: higher p-values and red: lower p-values) and pathway impact values (the larger the circle the higher the impact score) calculated from the topological analysis, respectively. Pathways were considered significantly enriched if p < 0.05, impact >0.1 and number of metabolite hits in the pathway >1.

We further carried out the metabolite pathway analysis using all metabolites discovered through the IVW approach (P<0.05). Through forward MR Analysis, we detected six potential metabolic pathways for thyroid cancer and four for Hashimoto’s thyroiditis. (**Table S7**). The results show that “Aminoacyl-tRNA biosynthesis” (P=0.002), “Phenylalanine, tyrosine and tryptophan biosynthesis” (P=0.007) pathway might be participated in the genesis of TC (**Figure 3A**). “D-Glutamine and D-glutamate metabolism” (P=0.01) and “Butanoate metabolism” (P=0.03) pathway might be associated with HT (**Figure 3B**). Among them, there exists a common metabolic pathway (i.e., “Arginine biosynthesis” and “Histidine metabolism”) shared by TC (P=0.02, P=0.03) and HT (P=0.03, P=0.04).

In reverse MR Analysis, we observed that TC (**Figure 3C**) and GD (**Figure 3D**) may increase or decrease the levels of related metabolites *in vivo* through four potential metabolic pathways. Among them, “arginine biosynthesis”, “D-glutamine and D-glutamate metabolism”, and “nitrogen metabolism” were three shared metabolic pathways present in both TC and GD (P < 0.05) (**Table S8**).

## Discussion

Observational studies have reported associations between metabolites and certain types of thyroid disorders. In this study, we conducted a two-sample bidirectional Mendelian Randomization (MR) analysis using publicly available GWAS summary statistics to investigate the causal relationships between human blood metabolites and various thyroid disorders, including Graves’ disease, Hashimoto’s thyroiditis, and thyroid cancer. Our findings revealed intricate interactions between metabolites and thyroid diseases. To the best of our knowledge, this represents the inaugural MR investigation into establishing causality between blood metabolites and thyroid disorders. In the forward MR Analysis, we found a total of 46 suggestive associations. Thyroid disease is linked to risk factors like Aspartate and protective factors like C-glycosyltryptophan. In the replication analysis, the results revealed that each of the three thyroid disorders had one overlapping metabolite in different datasets. Extensive sensitivity analyses showed that these associations were robust to the pleipotency of the MR Methods and tools used, using MR-PRESSO and leave-one analyses, and showed consistent findings.

Over the past decade, continuous discoveries of metabolic biomarkers related to thyroid disorders have emerged in the field of metabolomics research. Tissues, blood, urine, and feces are typical sample sources for metabolomic profiling. Among these, blood serves as a rich reservoir of readily accessible metabolites, making it valuable for identifying circulating biomarkers in thyroid disease screening. In metabolomic studies pertaining to plasma/serum, alterations in the metabolic profiles of three thyroid disorders have been observed, with the most common metabolic categories being amino acids, fatty acids, and lipids. For instance, in a study by Zhao et al. (31), metabolic differences were compared between thyroid cancer patients and healthy controls. This analysis revealed a significant increase in serum amino acid metabolite levels in thyroid cancer patients, while levels of lipid and choline metabolites were comparatively lower. Our research indicates that Aspartate, Stearoylcarnitine, and Gamma-glutamylglutamine are associated with a higher risk of thyroid cancer. Furthermore, after the onset of TC and GD, there is a decrease in glutamine levels. In TC patients, Taurolithocholate 3-sulfate increases, while GD patients exhibit higher levels of N2,N2-dimethylguanosine. Similar results were reported by Liu et al. (9) in their serum metabolomics pattern analysis of autoimmune thyroid disease patients, revealing differences in amino acids, fatty acids, and lipid-related metabolites among all subjects. Conversely, the occurrence of thyroid disorders also leads to fluctuations in metabolite levels. Patients with thyroid dysfunction exhibit more significant variations in metabolite levels. In our reverse MR analysis, we identified 23 metabolites with suggestive associations, suggesting that the onset of TC and GD alters the composition of metabolites. These findings may have implications for public health interventions aimed at reducing the risk of thyroid disease.

This MR analysis additionally recognized specific metabolites, some of which had been previously reported in other studies. C-glycosyltryptophan is a blood metabolite in the tryptophan pathway, and as a strong protective factor of TC, it has been shown to have a causal association with chronic kidney disease (CKD) in Cheng et al. (32). In our study, phenylalanine increased the risk of TC and kynurenine increased the risk of GD. This echoes prior discoveries. For instance, in line with metabolomics, heightened phenylalanine levels were observed in TC patients, and seven metabolites, including kynurenine, showed marked elevation in GD patients when compared to healthy controls (33). Nonetheless, the sample sizes in these investigations span from dozens to hundreds, which might not adequately represent the broader population. The metabolite and thyroid disease data in this study included a larger sample size, making this study more representative. Carnitine is a quaternary ammonium compound synthesized from phosphatidylcholine, as well as the amino acids lysine and methionine. It has been studied as a therapy or protective agent for many diseases (34). Marie-Josée et al. discovered (35) that carnitine can enhance the *in vivo* bioavailability of butyrate. When combined, butyrate—a potential anticancer compound—can inhibit proliferation and induce apoptosis in human colon cancer cells. Another study (36) confirmed that carnitine reduced the effect of butyrate as an HDAC inhibitor and restrained the induction of H3 acetylation by butyrate in colorectal cancer cells. Our results confirm that carnitine is a protective factor for TC, further research is needed to explore the potential mechanisms underlying the combined impact of carnitine and butyrate in thyroid cancer. γ-tocopherol has demonstrated a protective effect against HT in two different GWAS databases. Previous analyses have reported unique antioxidant and anti-inflammatory activities associated with γ-tocopherol, suggesting its potential role in preventing thyroid disorders through the regulation of inflammation-related mechanisms (37). Future functional analysis is necessary, substantial experimental endeavors are still required in later stages to establish a more precise assessment of the initial speculation.

In this study, we identified ten significant metabolic pathways associated with TC and HT through pathway enrichment analysis. Among these pathways, “Arginine Biosynthesis” and “Histidine Metabolism” were found to be linked to both TC and HT. Medullary thyroid carcinoma is a malignant tumor originating from the C-cells of the thyroid, which synthesize and secrete calcitonin (38, 39), with arginine serving as a precursor for calcitonin synthesis (40). Patients with this condition typically require lifelong arginine supplementation to maintain normal arginine levels in their bodies. Additionally, thyroid hormone synthesis depends on both arginine and histidine (41), with tyrosine in the histidine metabolism pathway being a precursor for thyroid hormone synthesis (42). In individuals with Hashimoto’s thyroiditis, the production of anti-thyroid peroxidase antibodies (anti-TPO antibodies) and anti-thyroglobulin antibodies can lead to attacks on thyroid tissue, affecting thyroid hormone synthesis and causing inflammation and damage (43). Furthermore, we also identified “Aminoacyl-tRNA Biosynthesis” as the most strongly associated pathway with TC, while “D-Glutamine and D-Glutamate Metabolism” pathway was primarily linked to GD and HT. Aminoacyl-tRNA is synthesized through esterification of tRNA with the appropriate amino acid at its 3’-end. Oxidative stress induces the rapid translocation of TyrRS from the cytoplasm to the nucleus to prevent DNA damage (44). Inhibiting tRNA aminoacylation has proven to be an effective antibacterial strategy, impeding a critical step in protein synthesis. Furthermore, Yu et al. (45) observed microbial changes in TCs that led to alterations in aminoacyl-tRNA biosynthesis. In humans (46), autosomal recessive mutations in glutamate-pyruvate transaminase 2 (GPT2) cause a neurological syndrome characterized by intellectual disability, microcephaly, and progressive motor symptoms. Thyroid dysfunction is a major contributor to muscle weakness. Thyroid hormones (TH) serve as crucial metabolic regulators that coordinate short-term and long-term energy demands (47). Related research has shown that THs regulate glutamine and glucose metabolism through GPT2, coupling glycolysis with the TCA cycle to maintain muscle mass. In summary, these findings suggest that aminoacyl-tRNA biosynthesis and glutamine metabolism may play vital roles in the biological mechanisms of thyroid disorders. Our research findings provide a profound perspective in understanding the relationship between metabolites and thyroid disorders, revealing strong associations between various metabolites and thyroid diseases. These insights pave the way for future investigations to enhance the diagnosis, prevention, and treatment of thyroid disorders.

This study boasts several key strengths. Firstly, it stands as the most comprehensive MR investigation concerning the metabolite-thyroid disease association, boasting the largest sample size to date. Secondly, we meticulously opted for GWAS data from the FINNGEN Consortium as a primary analysis, and corroborated our findings using IEU Open GWAS datasets, markedly enhancing result reliability. Thirdly, both reverse MR Analysis and sensitivity analysis indicated the absence of pleiotropy or heterogeneity, underscoring the statistical robustness of our outcomes.

Nonetheless, certain limitations should be acknowledged in this study. Initially, owing to the constrained count of SNPs achieving genome-wide significance, we opted for a relaxed P threshold—a prevalent practice employed in similar contexts. Furthermore, the veracity of SNPs with relaxed P-values is corroborated by the genuine direction ascertained from the Steiger test. Secondly, while it’s generally advisable to employ sizable GWAS sample sizes for MR Studies, our research employed a relatively modest metabolite GWAS sample size, potentially impacting the robustness of our MR findings. Another limitation pertains to the predominantly European ancestry of our participants. Caution must be exercised when generalizing these findings to other ethnic groups, necessitating further investigation. Fourthly, metabolite levels exhibit variation across diverse cell and tissue types. Our study solely examines the causal link between blood-measured metabolites and thyroid disease, failing to address the relevance of metabolite levels in more biologically pertinent tissues, such as the thyroid. Finally, post Bonferroni correction, we didn’t ascertain a distinct causal connection between metabolites and thyroid disease, implying the requirement for additional research to corroborate this relationship.

## Conclusion

In summary, this study establishes a bidirectional causal link between human blood metabolites and thyroid disease. Our forward MR Analysis pinpointed 46 metabolites potentially influencing thyroid disease progression, while reverse MR Analysis identified 23 metabolites possibly influenced by thyroid disease development. Additionally, potential metabolic pathways underlying metabolite-thyroid disease association were identified. Yet, comprehensive clinical investigations are required to unveil the precise metabolite-thyroid disease relationship and unravel the mechanistic underpinnings further.

## Data availability statement

The datasets presented in this study can be found in online repositories. The names of the repository/repositories and accession number(s) can be found in the article/**Supplementary Material**.

## Author contributions

ZL: Funding acquisition, Project administration, Resources, Writing – review & editing. XZ: Writing – original draft, Writing – review & editing. JZ: Conceptualization, Investigation, Software, Writing – review & editing. ZX: Data curation, Investigation, Methodology, Writing – review & editing. XL: Data curation, Formal Analysis, Project administration. JH: Writing – review & editing, Data curation, Formal Analysis, Project administration. HH: Writing – review & editing, Project administration, Validation, Visualization.
