# Supplementary material for: Exploring blood metabolites and thyroid disorders: a bidirectional mendelian randomization study
Source: Front Endocrinol (Lausanne). 2023 Oct 9;14:1270336. doi: 10.3389/fendo.2023.1270336 (PMC10591305; doi:10.3389/fendo.2023.1270336)
Supplement: Supplementary file 1 [file DataSheet_1.pdf]

Supplementary Figure Legends

Figure S1. Scatter plots showing the causal association of ten metabolites on the risk of thyroid cancer. **(**A)Phenylalanine, (B)Aspartate, (C)C-glycosyltryptophan*, (D)Carnitine, (E)1-linoleoylglycerol (1-monolinolein), (F)Stearoylcarnitine, (G)Gamma-glutamylglutamine, (H)Gamma-glutamylleucine, (I)Uridine, (J)Myristoleate (14:1n5)

Figure S2. Forest plots for the ten potential metabolites on thyroid cancer.

Figure S3. Funnel plots for the ten potential metabolites on thyroid cancer.

Figure S4. Leave-one-out plots for the ten potential metabolites on thyroid cancer.

Figure S5. Scatter plots showing the causal association of five metabolites on the risk of Graves' disease. **(**A) Kynurenine, (B) Taurochenodeoxycholate, (C) 4-androsten-3beta,17beta-diol disulfate 2*, (D) Phenylalanylphenylalanine, (E) Phosphate

Figure S6. Forest plots for the five potential metabolites on Graves' disease.

Figure S7. Funnel plots for the five potential metabolites on Graves' disease.

Figure S8. Leave-one-out plots for the five potential metabolites on Graves' disease.

Figure S9. Scatter plots showing the causal association of seven metabolites on the risk of Hashimoto thyroiditis. **(**A) Kynurenine, (B) 3-methylhistidine, (C) Phenol sulfate, (D) 2-palmitoylglycerophosphocholine*, (E) X-14189—leucylalanine, (F) Gamma-tocopherol, (G) Alpha-ketoglutarate

Figure S10. Forest plots for the seven potential metabolites on Hashimoto thyroiditis.

Figure S11. Funnel plots for the seven potential metabolites on Hashimoto thyroiditis.

Figure S12. Leave-one-out plots for the seven potential metabolites on Hashimoto thyroiditis.

Figure S13. Scatter plots for the thyroid cancer on five potential metabolites. **(**A) Glutamine, (B) Ornithine, (C) 5-oxoproline, (D) X-12095--N1-methyl-3-pyridone-4-carboxamide, (E) Taurolithocholate 3-sulfate

Figure S14. Forest plots for the thyroid cancer on five potential metabolites.

Figure S15. Funnel plots for the thyroid cancer on five potential metabolites.

Figure S16. Leave-one-out plots for the thyroid cancer on five potential metabolites.

Figure S17. Scatter plots for the Graves' disease on thirteen potential metabolites. (A) Glutamine, (B) Stearate (18:0), (C) 2-hydroxystearate, (D) 1-oleoylglycerol (1-monoolein), (E) 2-hydroxyisobutyrate, (F) 3-(4-hydroxyphenyl)lactate, (G) Scyllo-inositol, (H) 1-arachidonoylglycerophosphocholine*, (I) 1-palmitoleoylglycerophosphocholine*, (J) 1-docosahexaenoylglycerophosphocholine*, (K) N2,N2-dimethylguanosine, (L) Glutaroyl carnitine, (M) 1-myristoylglycerophosphocholine

Figure S18. Forest plots for the Graves' disease on thirteen potential metabolites.

Figure S19. Funnel plots for the Graves' disease on thirteen potential metabolites.

Figure S20. Leave-one-out plots for the Graves' disease on thirteen potential metabolites.

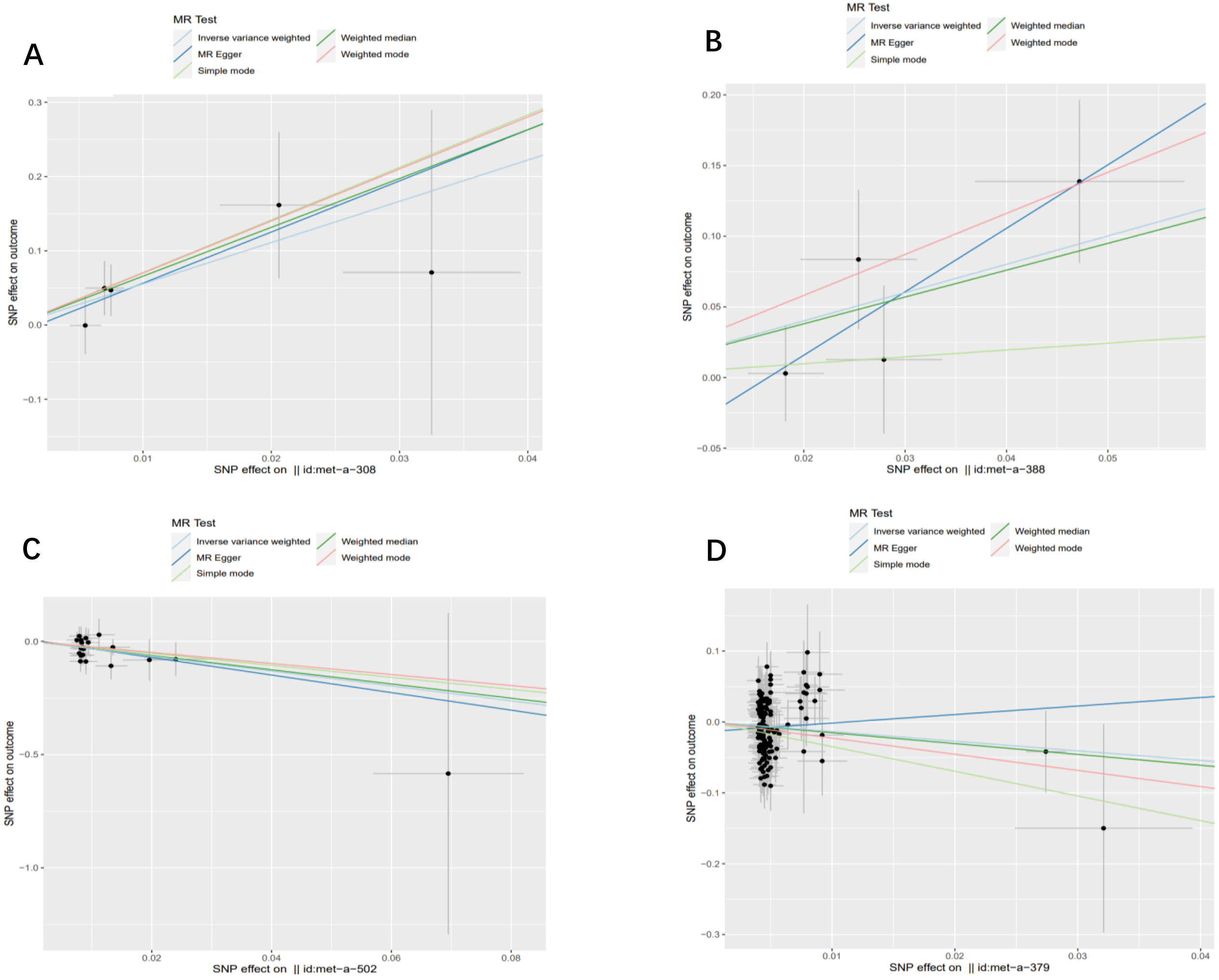
A
B
C
D
MR Test
Inverse variance weighted
MR Egger
Simple mode
Weighted median
Weighted mode
SNP effect on outcome
SNP effect on || id:met−a−308
SNP effect on || id:met−a−388
SNP effect on || id:met−a−502
SNP effect on || id:met−a−379

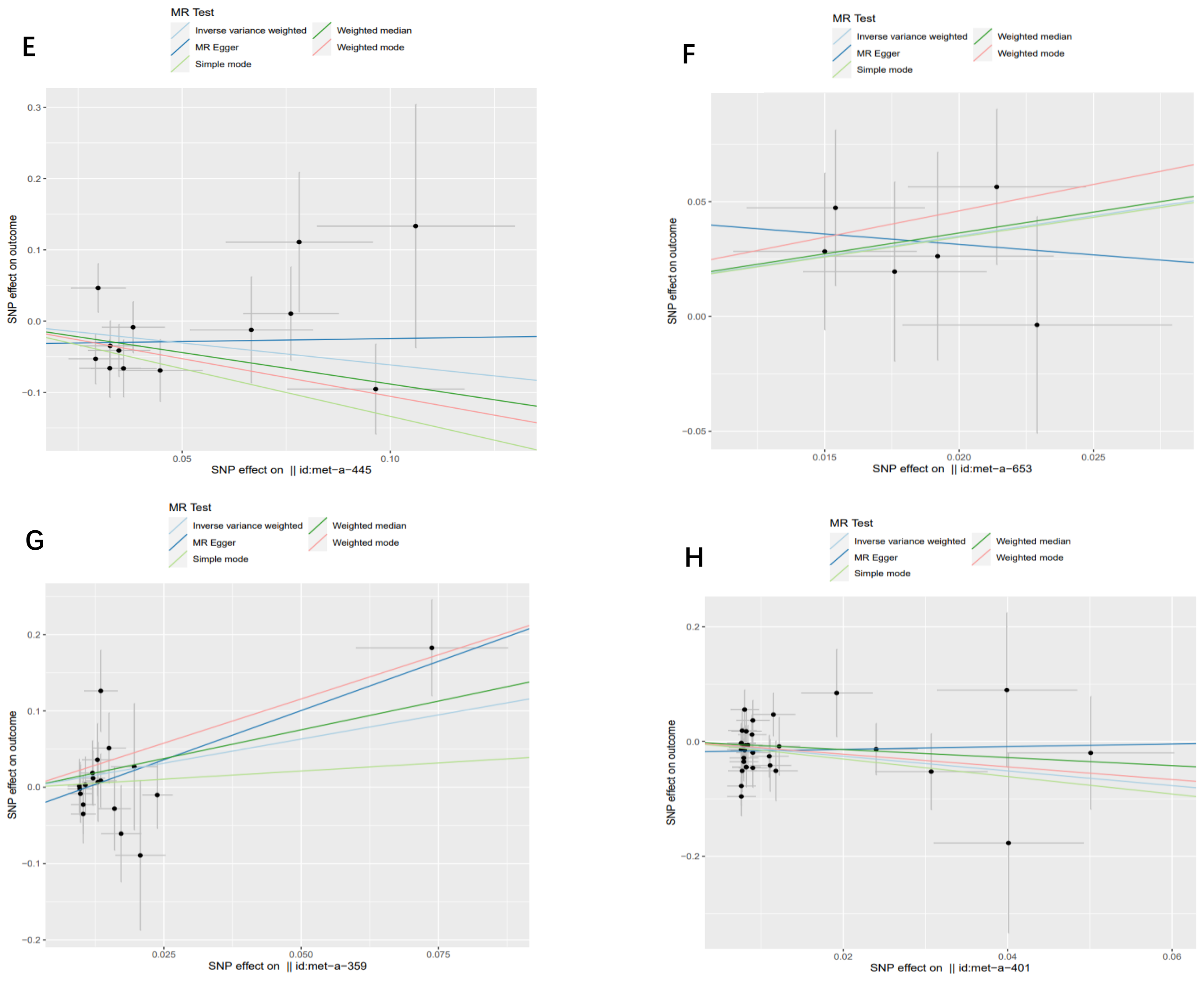

E
MR Test
Inverse variance weighted
MR Egger
Simple mode
Weighted median
Weighted mode
SNP effect on outcome
0.3
0.2
0.1
0.0
−0.1
0.05
0.10
SNP effect on || id:met−a−445
F
MR Test
Inverse variance weighted
MR Egger
Simple mode
Weighted median
Weighted mode
SNP effect on outcome
0.05
0.00
−0.05
0.015
0.020
0.025
SNP effect on || id:met−a−653
G
MR Test
Inverse variance weighted
MR Egger
Simple mode
Weighted median
Weighted mode
SNP effect on outcome
0.2
0.1
0.0
−0.1
−0.2
0.025
0.050
0.075
SNP effect on || id:met−a−359
H
MR Test
Inverse variance weighted
MR Egger
Simple mode
Weighted median
Weighted mode
SNP effect on outcome
0.2
0.0
−0.2
0.02
0.04
0.06
SNP effect on || id:met−a−401

I

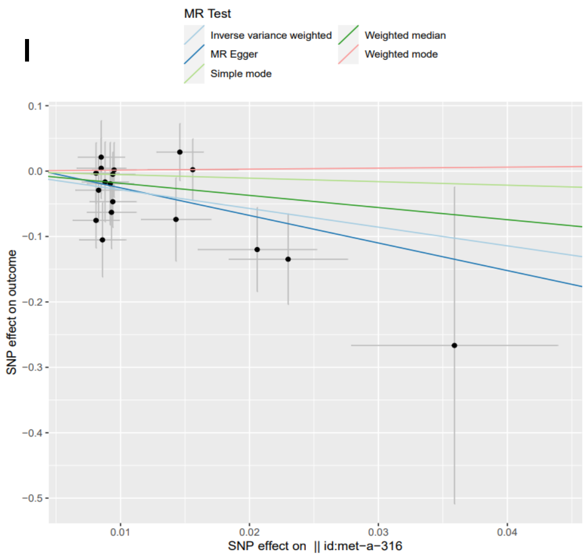

J

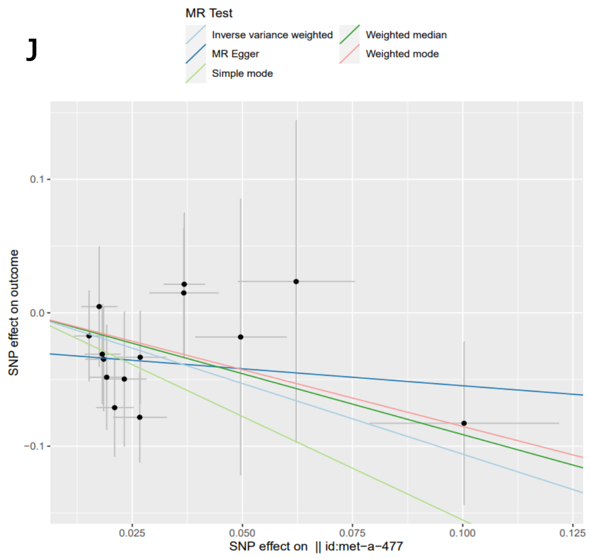

Figure S1 Scatter plots showing the causal association of ten metabolites on the risk of thyroid cancer. (A)Phenylalanine, (B)Aspartate, (C)C-glycosyltryptophan*, (D)Carnitine, (E)1-linoleoylglycerol (1-monolinolein), (F)Stearoylcarnitine, (G)Gamma-glutamylglutamine, (H)Gamma-glutamylleucine, (I)Uridine, (J)Myristoleate (14:1n5)

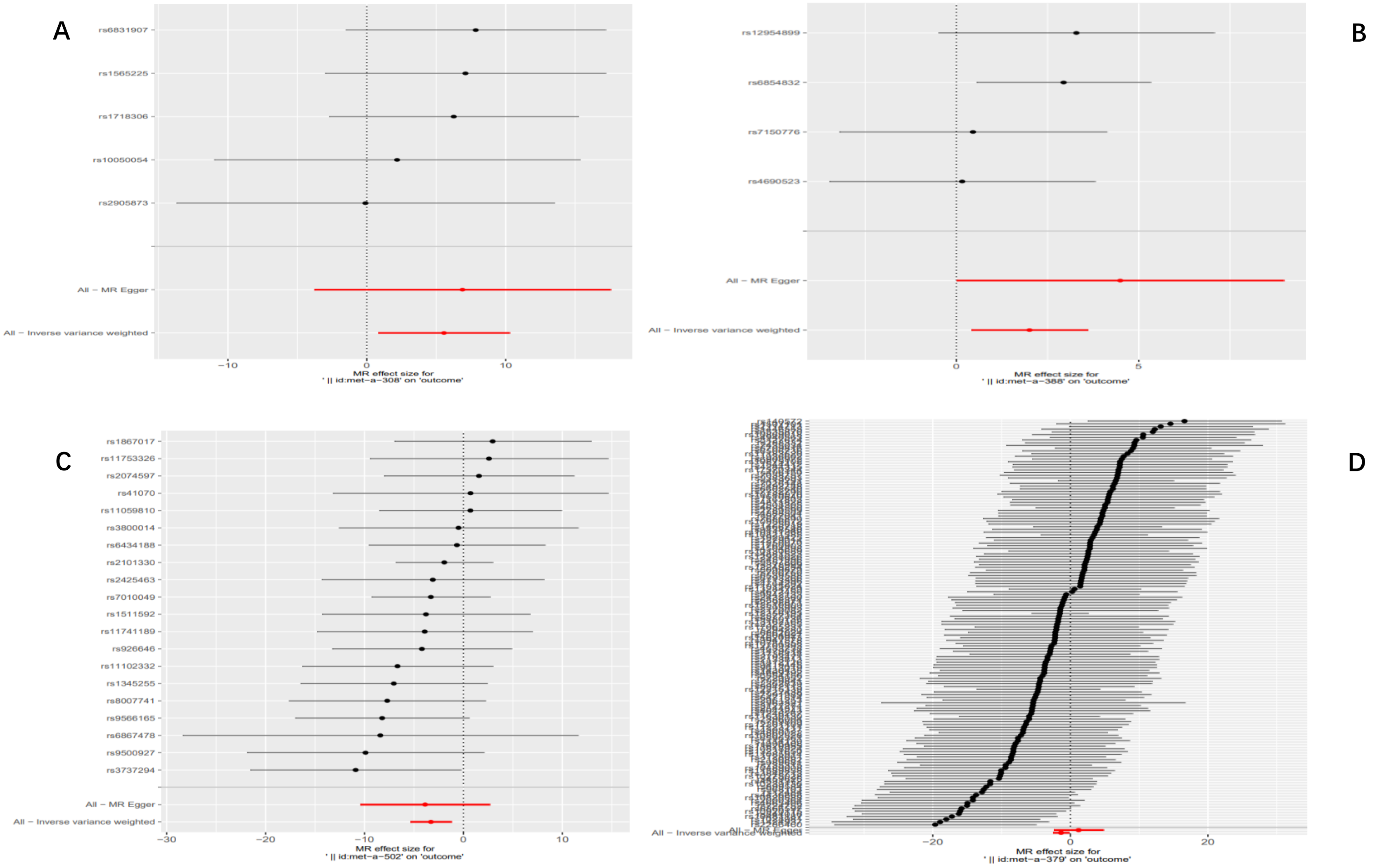
A
rs6831907
rs1565225
rs1718306
rs10050054
rs2905873
All − MR Egger
All − Inverse variance weighted
−10
0
10
MR effect size for
' || id:met−a−308' on 'outcome'
B
rs12954899
rs6854832
rs7150776
rs4690523
All − MR Egger
All − Inverse variance weighted
0
5
MR effect size for
' || id:met−a−388' on 'outcome'
C
rs1867017
rs11753326
rs2074597
rs41070
rs11059810
rs3800014
rs6434188
rs2101330
rs2425463
rs7010049
rs1511592
rs11741189
rs926646
rs11102332
rs1345255
rs8007741
rs9566165
rs6867478
rs9500927
rs3737294
All − MR Egger
All − Inverse variance weighted
−30
−20
−10
0
10
MR effect size for
' || id:met−a−502' on 'outcome'
D
All − Inverse variance weighted
−20
0
20
MR effect size for
' || id:met−a−379' on 'outcome'

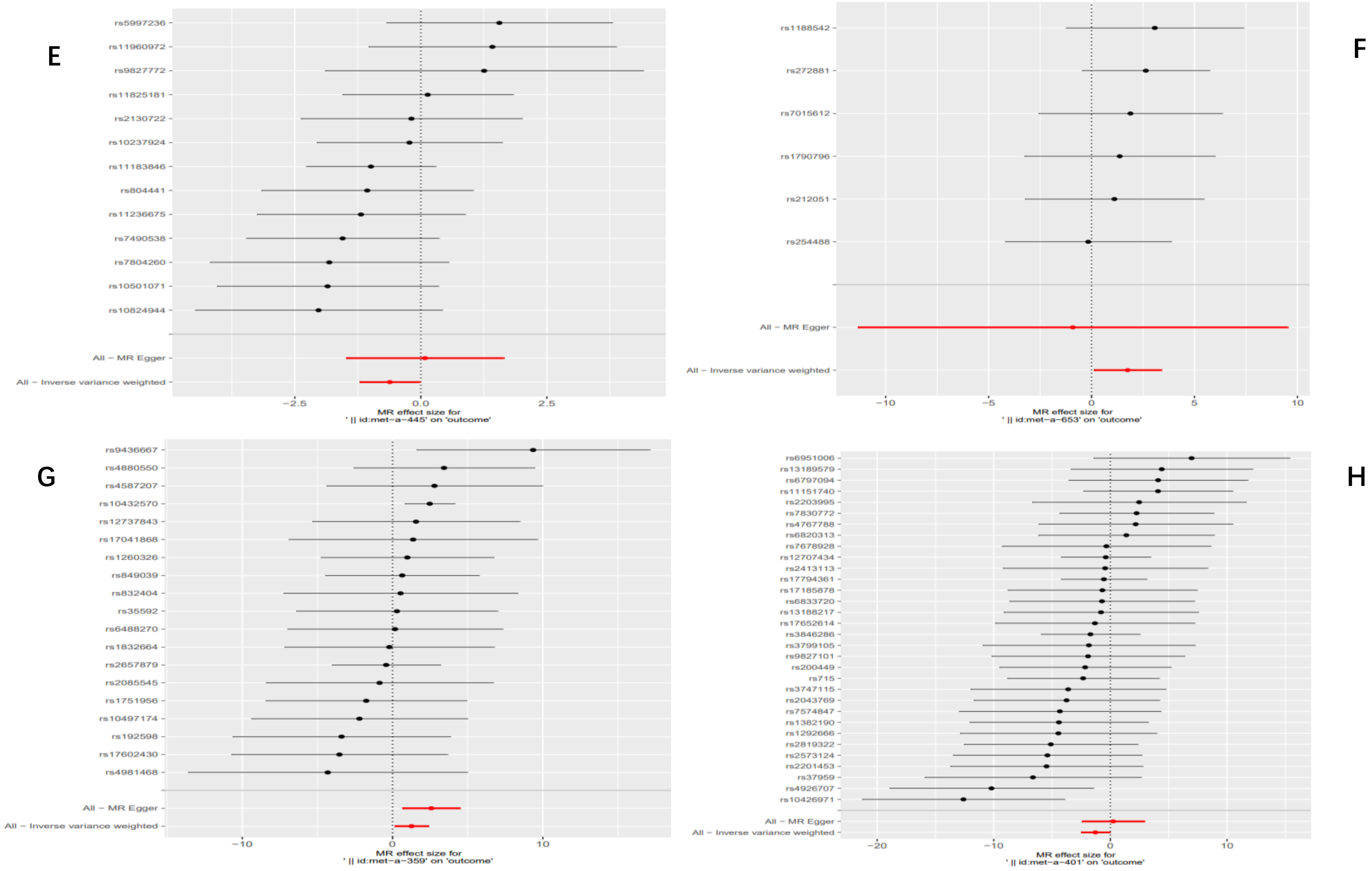
E
rs5997236
rs11960972
rs9827772
rs11825181
rs2130722
rs10237924
rs11183846
rs804441
rs11236675
rs7490538
rs7804260
rs10501071
rs10824944
All − MR Egger
All − Inverse variance weighted
−2.5
0.0
2.5
MR effect size for
' || id:met−a−445' on 'outcome'
F
rs1188542
rs272881
rs7015612
rs1790796
rs212051
rs254488
All − MR Egger
All − Inverse variance weighted
−10
−5
0
5
10
MR effect size for
' || id:met−a−653' on 'outcome'
G
rs9436667
rs4880550
rs4587207
rs10432570
rs12737843
rs17041868
rs1260326
rs849039
rs832404
rs35592
rs6488270
rs1832664
rs2657879
rs2085545
rs1751956
rs10497174
rs192598
rs17602430
rs4981468
All − MR Egger
All − Inverse variance weighted
−10
0
10
MR effect size for
' || id:met−a−359' on 'outcome'
H
rs6951006
rs13189579
rs6797094
rs11151740
rs2203995
rs7830772
rs4767788
rs6820313
rs7678928
rs12707434
rs2413113
rs17794361
rs17185878
rs6833720
rs13188217
rs17652614
rs3846286
rs3799105
rs9827101
rs200449
rs715
rs3747115
rs2043769
rs7574847
rs1382190
rs1292666
rs2819322
rs2573124
rs2201453
rs37959
rs4926707
rs10426971
All − MR Egger
All − Inverse variance weighted
−20
−10
0
10
MR effect size for
' || id:met−a−401' on 'outcome'

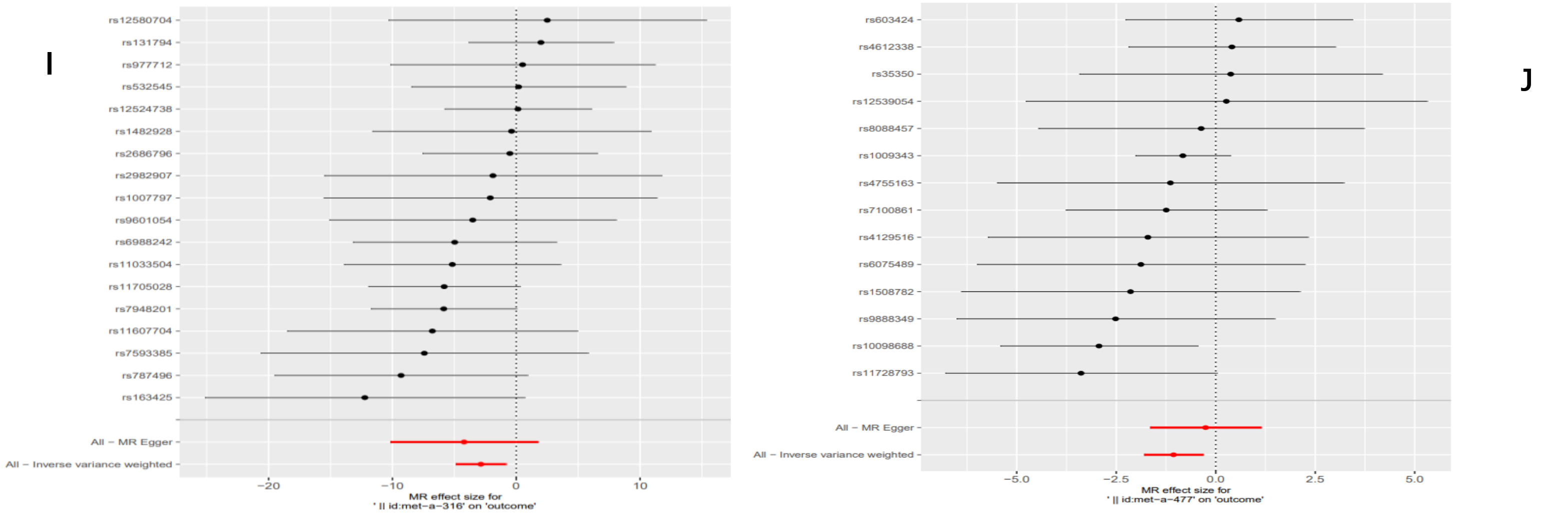

Figure S2 Forest plots for the ten potential metabolites on thyroid cancer. (A)Phenylalanine, (B)Aspartate, (C)C-glycosyltryptophan*, (D)Carnitine, (E)1-linoleoylglycerol (1-monolinolein), (F)Stearoylcarnitine, (G)Gamma-glutamylglutamine, (H)Gamma-glutamylleucine, (I)Uridine, (J)Myristoleate (14:1n5)

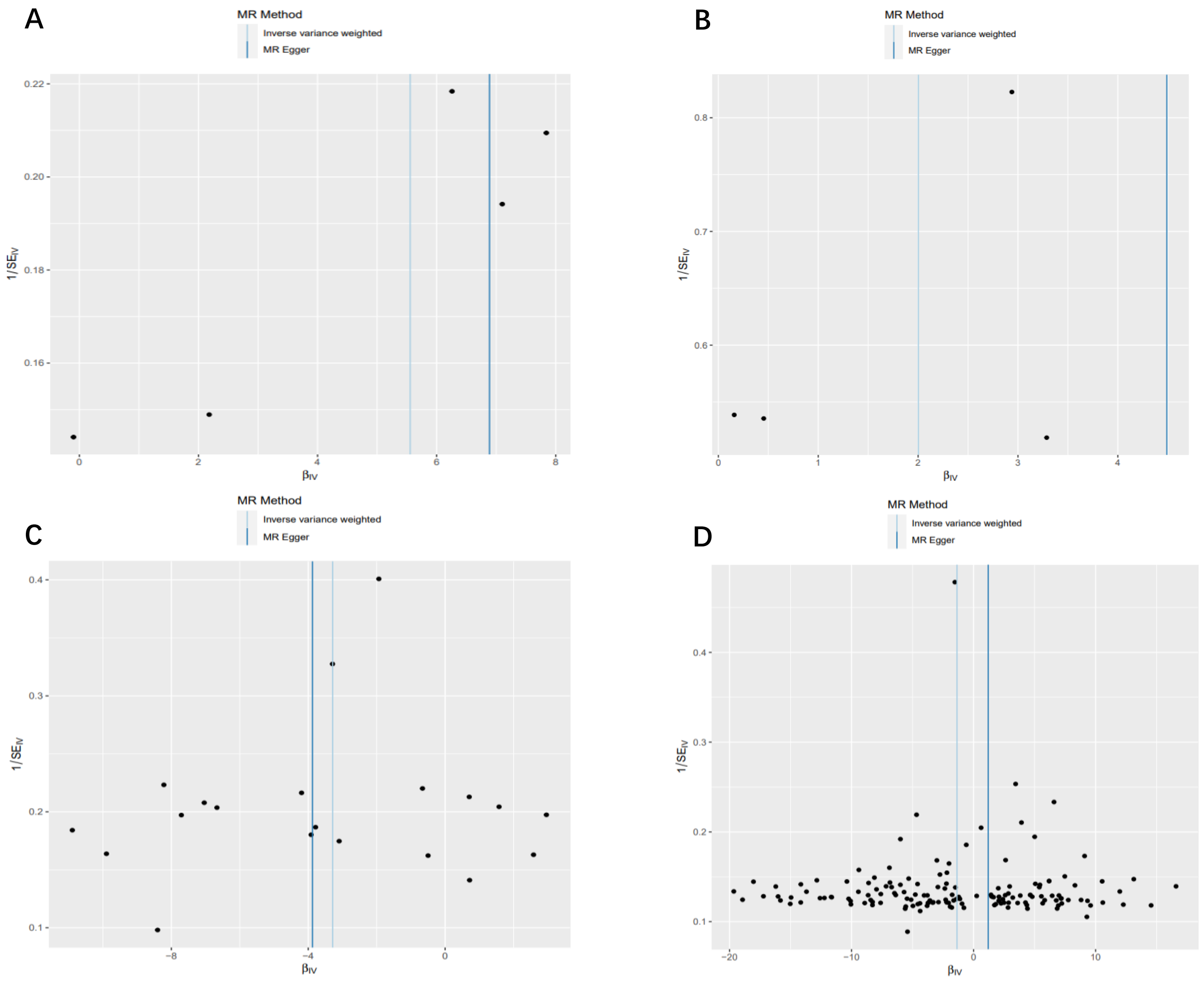
A
B
C
D
MR Method
Inverse variance weighted
MR Egger
1/SEIV
βIV

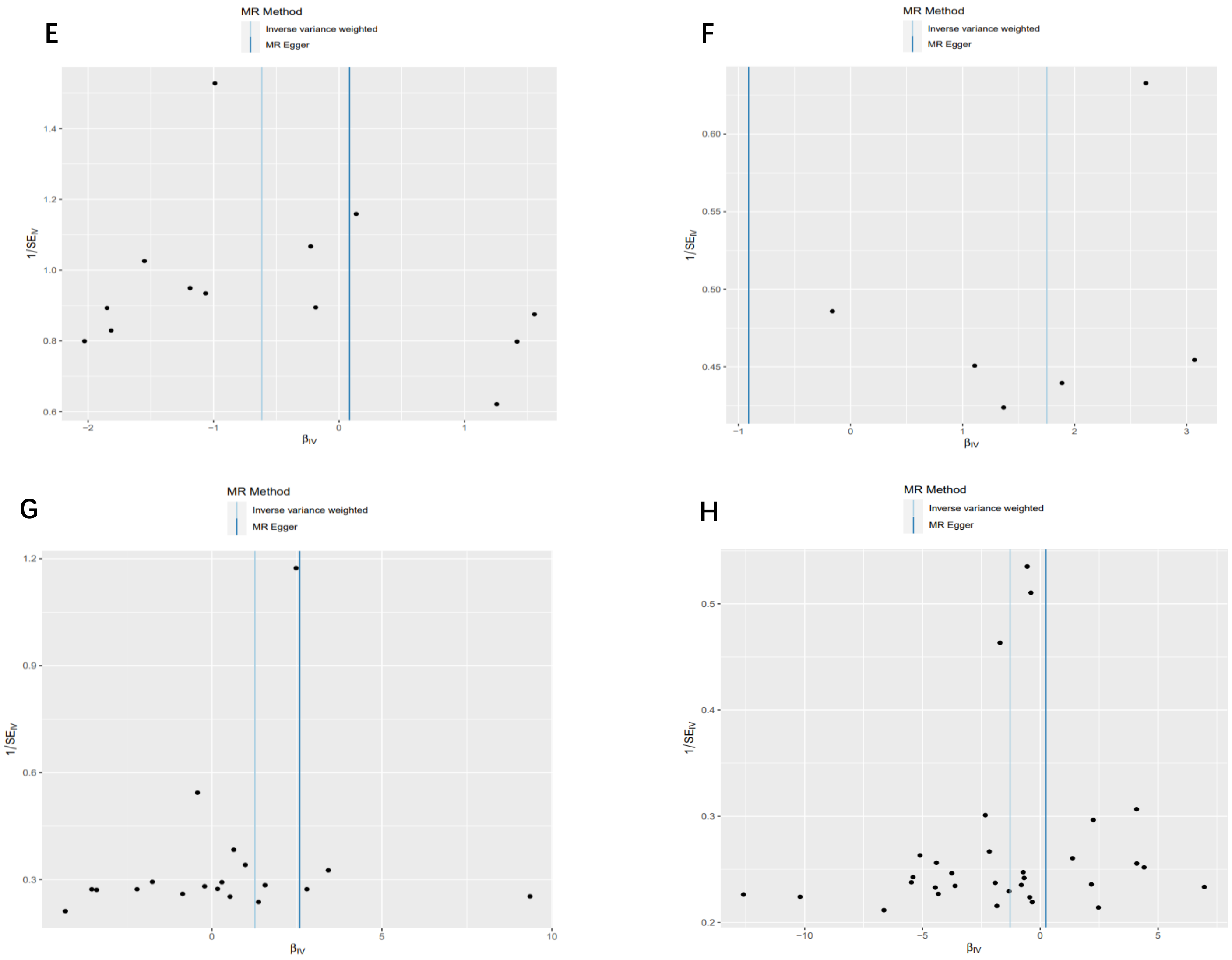
E
MR Method
Inverse variance weighted
MR Egger
1/SE_IV
β_IV
1.4
1.2
1.0
0.8
0.6
−2
−1
0
1
F
MR Method
Inverse variance weighted
MR Egger
1/SE_IV
β_IV
0.60
0.55
0.50
0.45
−1
0
1
2
3
G
MR Method
Inverse variance weighted
MR Egger
1/SE_IV
β_IV
1.2
0.9
0.6
0.3
0
5
10
H
MR Method
Inverse variance weighted
MR Egger
1/SE_IV
β_IV
0.5
0.4
0.3
0.2
−10
−5
0
5

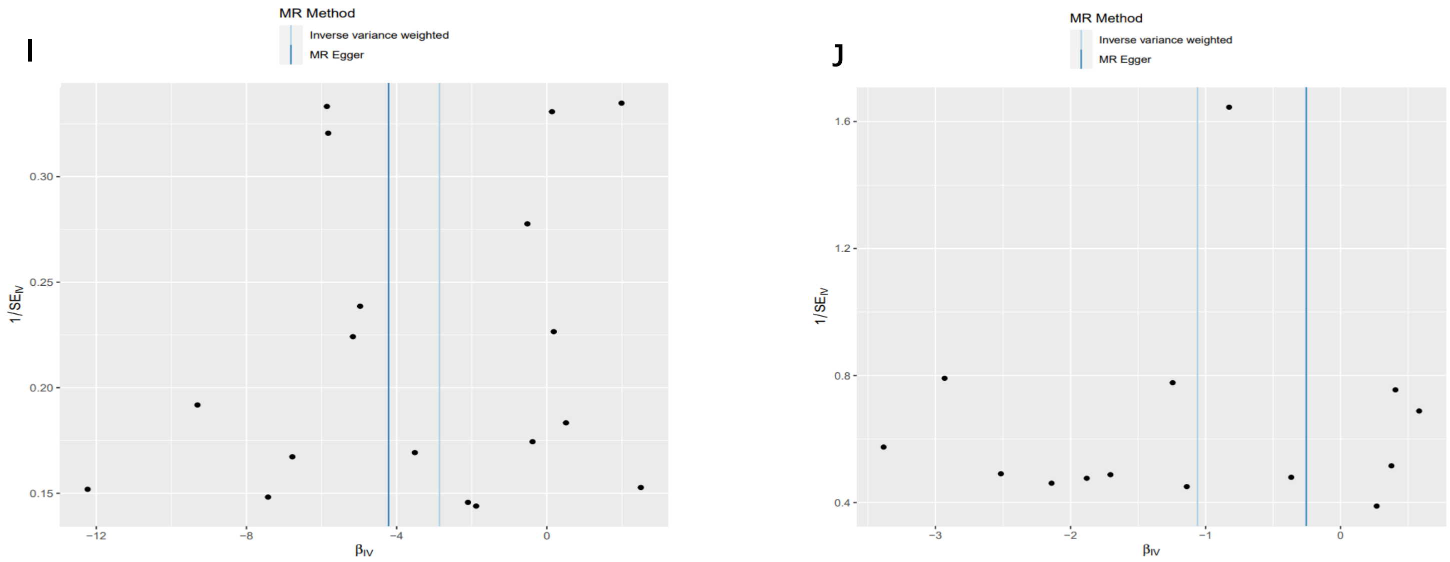

Figure S3 Funnel plots for the ten potential metabolites on thyroid cancer. (A)Phenylalanine, (B)Aspartate, (C)C-glycosyltryptophan*, (D)Carnitine, (E)1-linoleoylglycerol (1-monolinolein), (F)Stearoylcarnitine, (G)Gamma-glutamylglutamine, (H)Gamma-glutamylleucine, (I)Uridine, (J)Myristoleate (14:1n5)

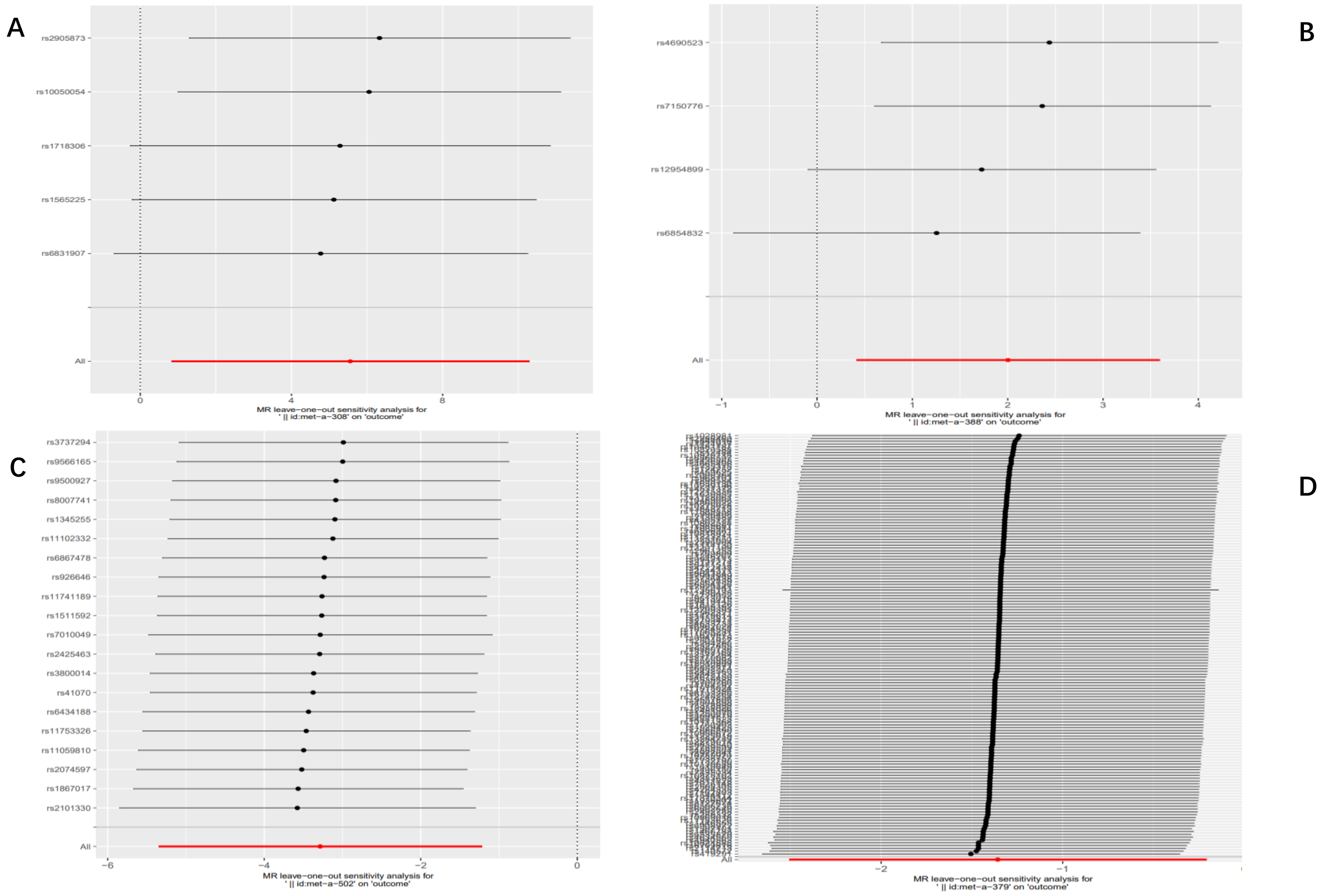
A
rs2905873
rs10050054
rs1718306
rs1565225
rs6831907
All
0
4
8
MR leave−one−out sensitivity analysis for
' || id:met−a−308' on 'outcome'
B
rs4690523
rs7150776
rs12954899
rs6854832
All
−1
0
1
2
3
4
MR leave−one−out sensitivity analysis for
' || id:met−a−388' on 'outcome'
C
rs3737294
rs9566165
rs9500927
rs8007741
rs1345255
rs11102332
rs6867478
rs926646
rs11741189
rs1511592
rs7010049
rs2425463
rs3800014
rs41070
rs6434188
rs11753326
rs11059810
rs2074597
rs1867017
rs2101330
All
−6
−4
−2
0
MR leave−one−out sensitivity analysis for
' || id:met−a−502' on 'outcome'
D
All
−2
−1
MR leave−one−out sensitivity analysis for
' || id:met−a−379' on 'outcome'

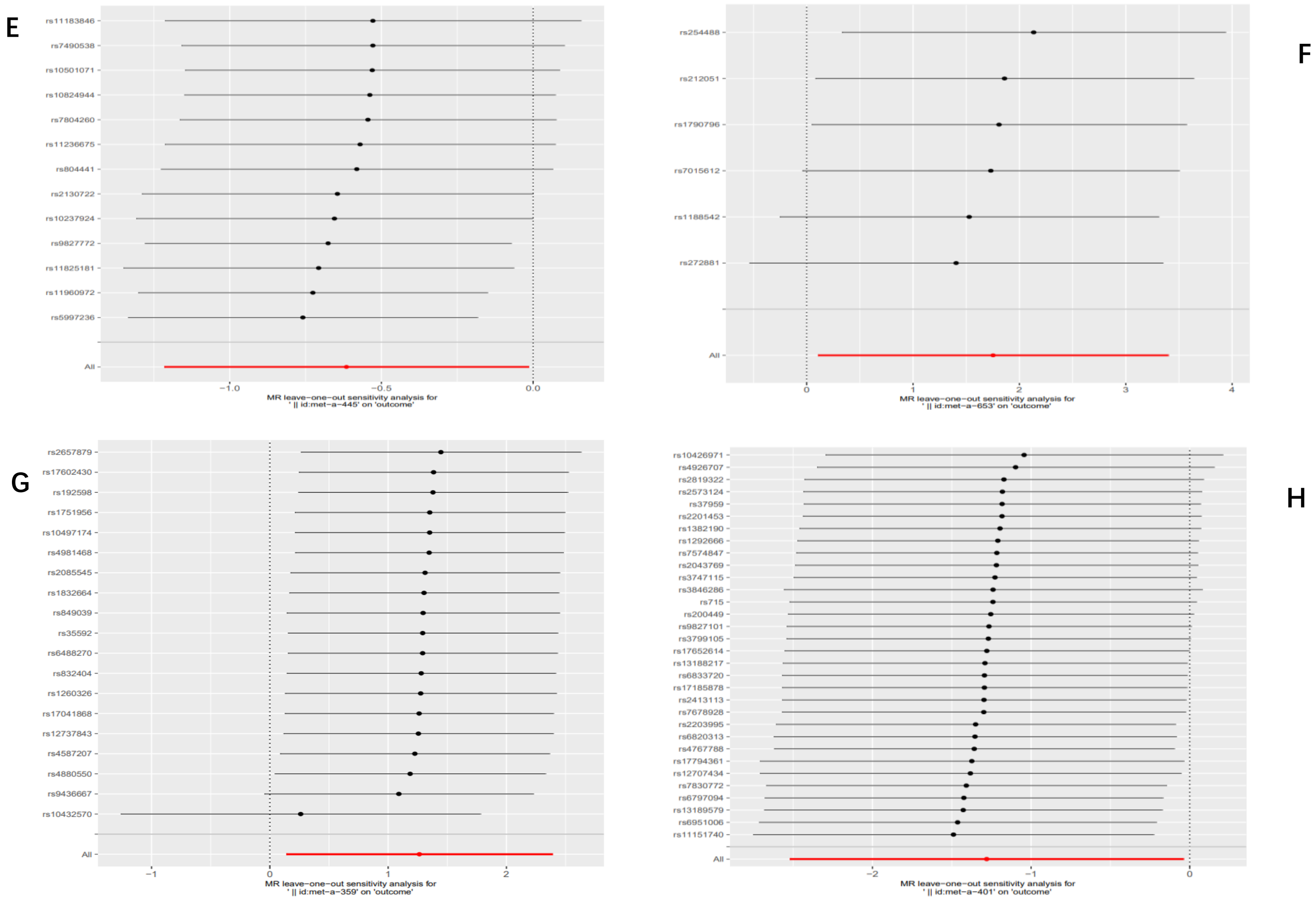
E
rs11183846
rs7490538
rs10501071
rs10824944
rs7804260
rs11236675
rs804441
rs2130722
rs10237924
rs9827772
rs11825181
rs11960972
rs5997236
All
−1.0
−0.5
0.0
MR leave−one−out sensitivity analysis for
' || id:met−a−445' on 'outcome'
F
rs254488
rs212051
rs1790796
rs7015612
rs1188542
rs272881
All
0
1
2
3
4
MR leave−one−out sensitivity analysis for
' || id:met−a−653' on 'outcome'
G
rs2657879
rs17602430
rs192598
rs1751956
rs10497174
rs4981468
rs2085545
rs1832664
rs849039
rs35592
rs6488270
rs832404
rs1260326
rs17041868
rs12737843
rs4587207
rs4880550
rs9436667
rs10432570
All
−1
0
1
2
MR leave−one−out sensitivity analysis for
' || id:met−a−359' on 'outcome'
H
rs10426971
rs4926707
rs2819322
rs2573124
rs37959
rs2201453
rs1382190
rs1292666
rs7574847
rs2043769
rs3747115
rs3846286
rs715
rs200449
rs9827101
rs3799105
rs17652614
rs13188217
rs6833720
rs17185878
rs2413113
rs7678928
rs2203995
rs6820313
rs4767788
rs17794361
rs12707434
rs7830772
rs6797094
rs13189579
rs6951006
rs11151740
All
−2
−1
0
MR leave−one−out sensitivity analysis for
' || id:met−a−401' on 'outcome'

I

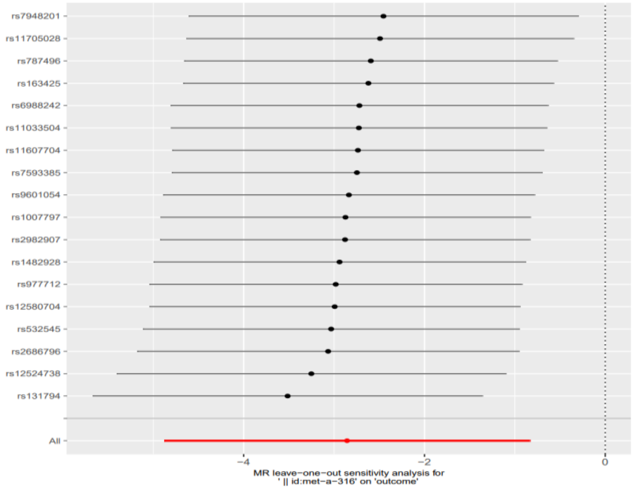

J

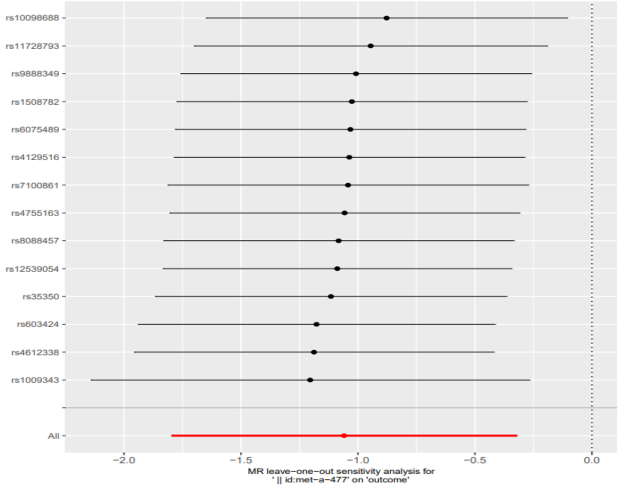

Figure S4 Leave-one-out plots for the ten potential metabolites on thyroid cancer. (A)Phenylalanine, (B)Aspartate, (C)C-glycosyltryptophan*, (D)Carnitine, (E)1-linoleoylglycerol (1-monolinolein), (F)Stearoylcarnitine, (G)Gamma-glutamylglutamine, (H)Gamma-glutamylleucine, (I)Uridine, (J)Myristoleate (14:1n5)

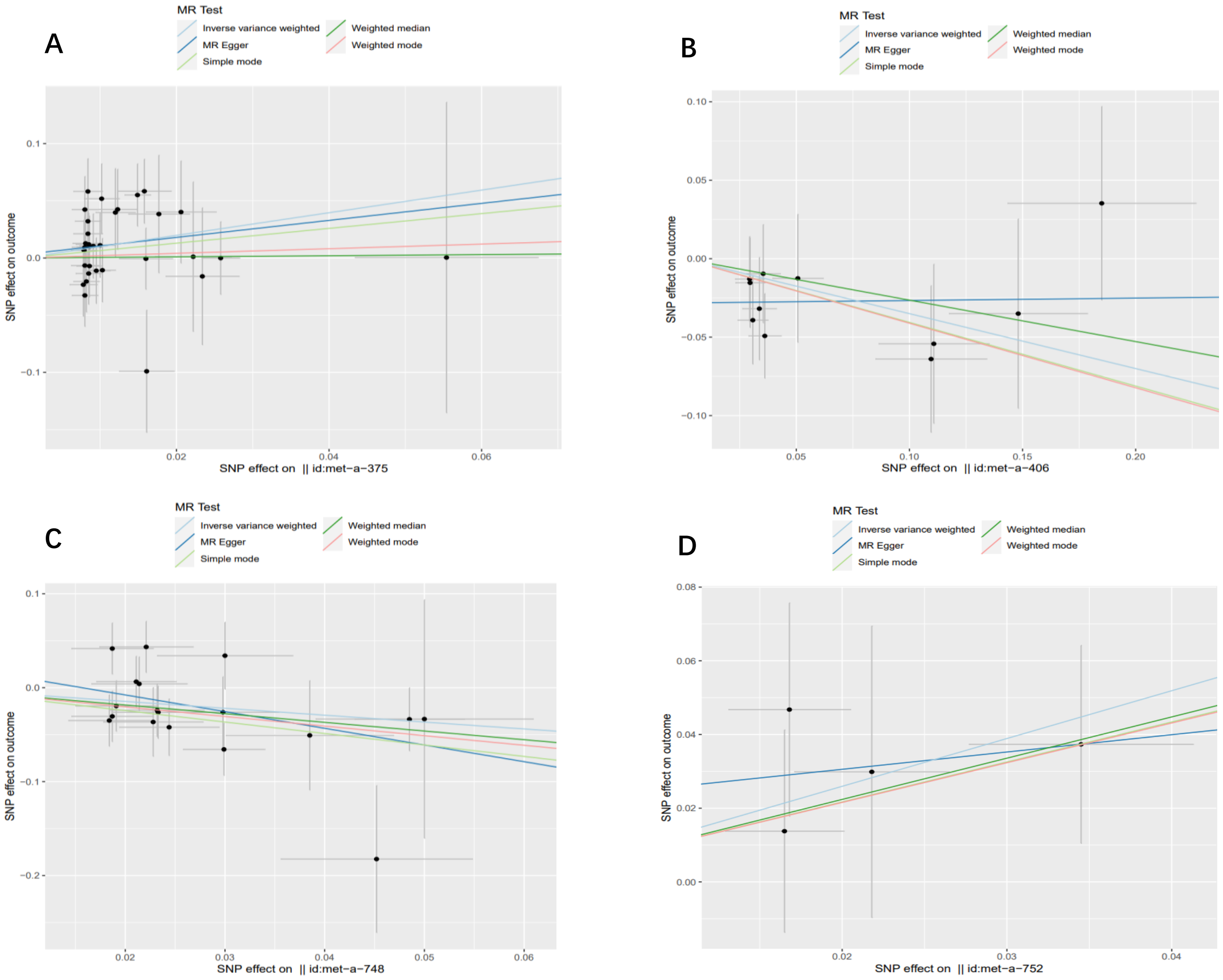
A
B
C
D
MR Test
Inverse variance weighted
MR Egger
Simple mode
Weighted median
Weighted mode
SNP effect on outcome
SNP effect on || id:met-a-375
SNP effect on || id:met-a-406
SNP effect on || id:met-a-748
SNP effect on || id:met-a-752

E

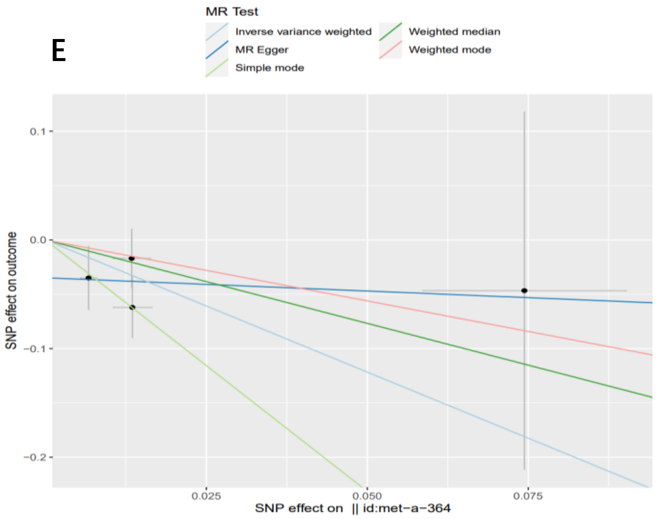

Figure S5 Scatter plots showing the causal association of five metabolites on the risk of Graves' disease. (A) Kynurenine, (B) Taurochenodeoxycholate, (C) 4-androsten-3beta,17beta-diol disulfate 2*, (D) Phenylalanylphenylalanine, (E) Phosphate

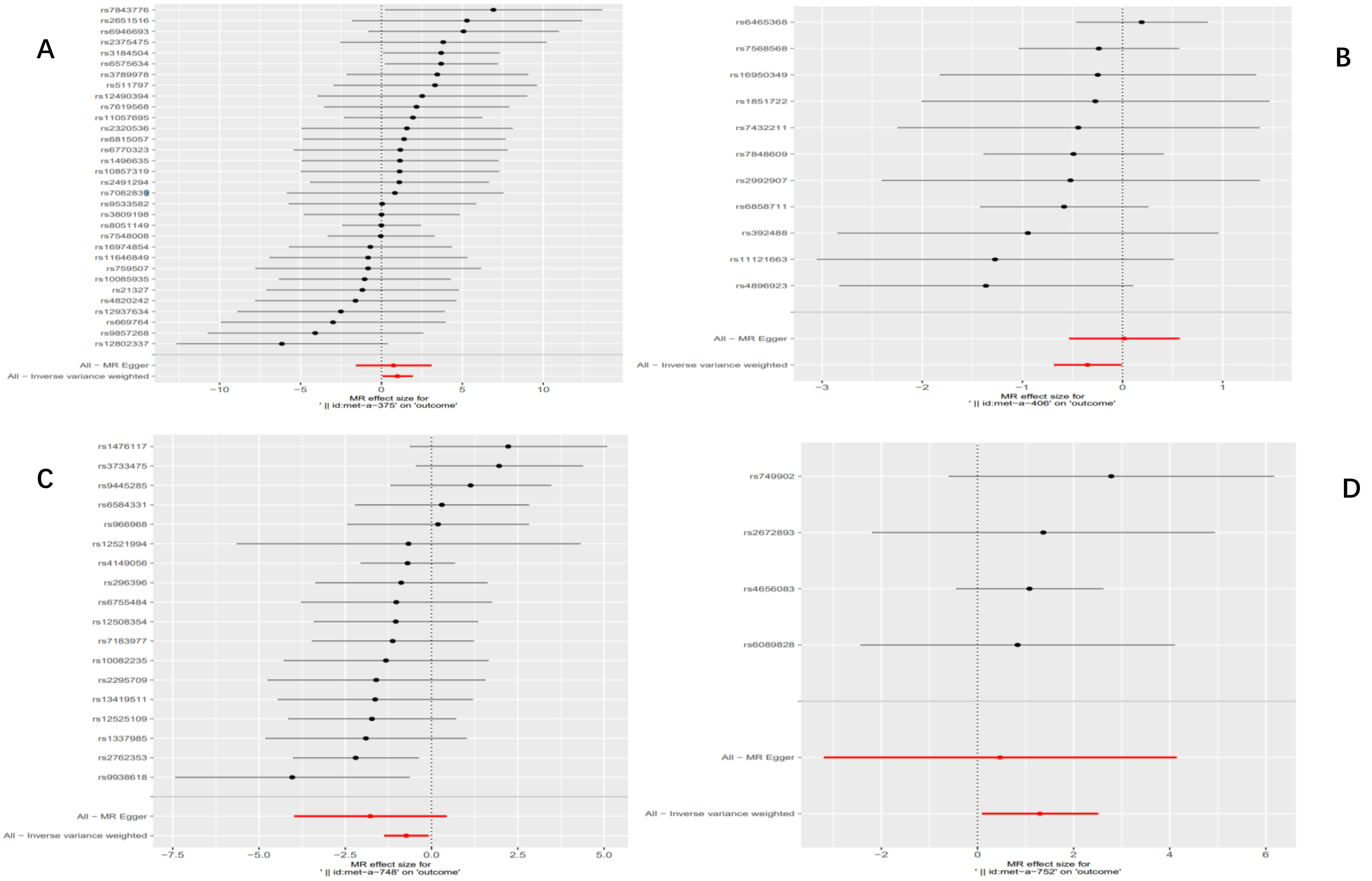
A
rs7843776
rs2651516
rs6946693
rs2375475
rs3184504
rs6575634
rs3789978
rs511797
rs12490394
rs7619568
rs11057695
rs2320536
rs6815057
rs6770323
rs1496635
rs10857319
rs2491294
rs7082839
rs9533582
rs3809198
rs8051149
rs7548008
rs16974854
rs11646849
rs759507
rs10085935
rs21327
rs4820242
rs12937634
rs669764
rs9857268
rs12802337
All − MR Egger
All − Inverse variance weighted
−10
−5
0
5
10
MR effect size for
' || id:met-a-375' on 'outcome'
B
rs6465368
rs7568568
rs16950349
rs1851722
rs7432211
rs7848609
rs2992907
rs6858711
rs392488
rs11121663
rs4896923
All − MR Egger
All − Inverse variance weighted
−3
−2
−1
0
1
MR effect size for
' || id:met-a-406' on 'outcome'
C
rs1476117
rs3733475
rs9445285
rs6584331
rs966968
rs12521994
rs4149056
rs296396
rs6755484
rs12508354
rs7183977
rs10082235
rs2295709
rs13419511
rs12525109
rs1337985
rs2762353
rs9938618
All − MR Egger
All − Inverse variance weighted
−7.5
−5.0
−2.5
0.0
2.5
5.0
MR effect size for
' || id:met-a-748' on 'outcome'
D
rs749902
rs2672893
rs4656083
rs6089828
All − MR Egger
All − Inverse variance weighted
−2
0
2
4
6
MR effect size for
' || id:met-a-752' on 'outcome'

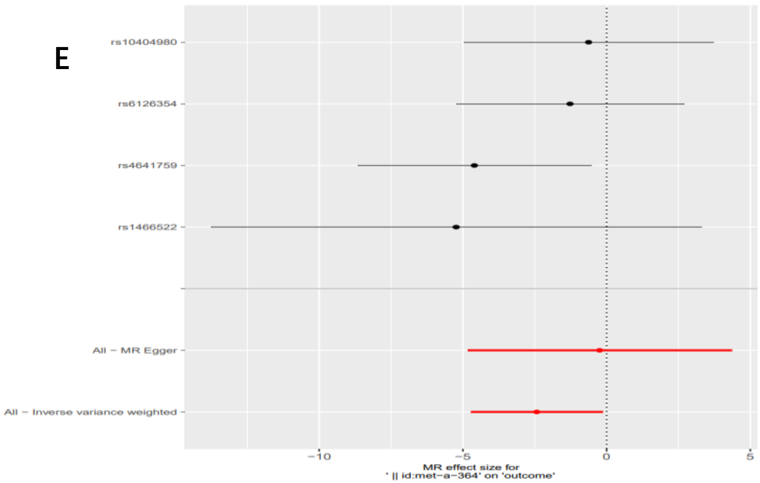

Figure S6 Forest plots for the five potential metabolites on Graves' disease. (A) Kynurenine, (B) Taurochenodeoxycholate, (C) 4-androsten-3beta,17beta-diol disulfate 2*, (D) Phenylalanylphenylalanine, (E) Phosphate

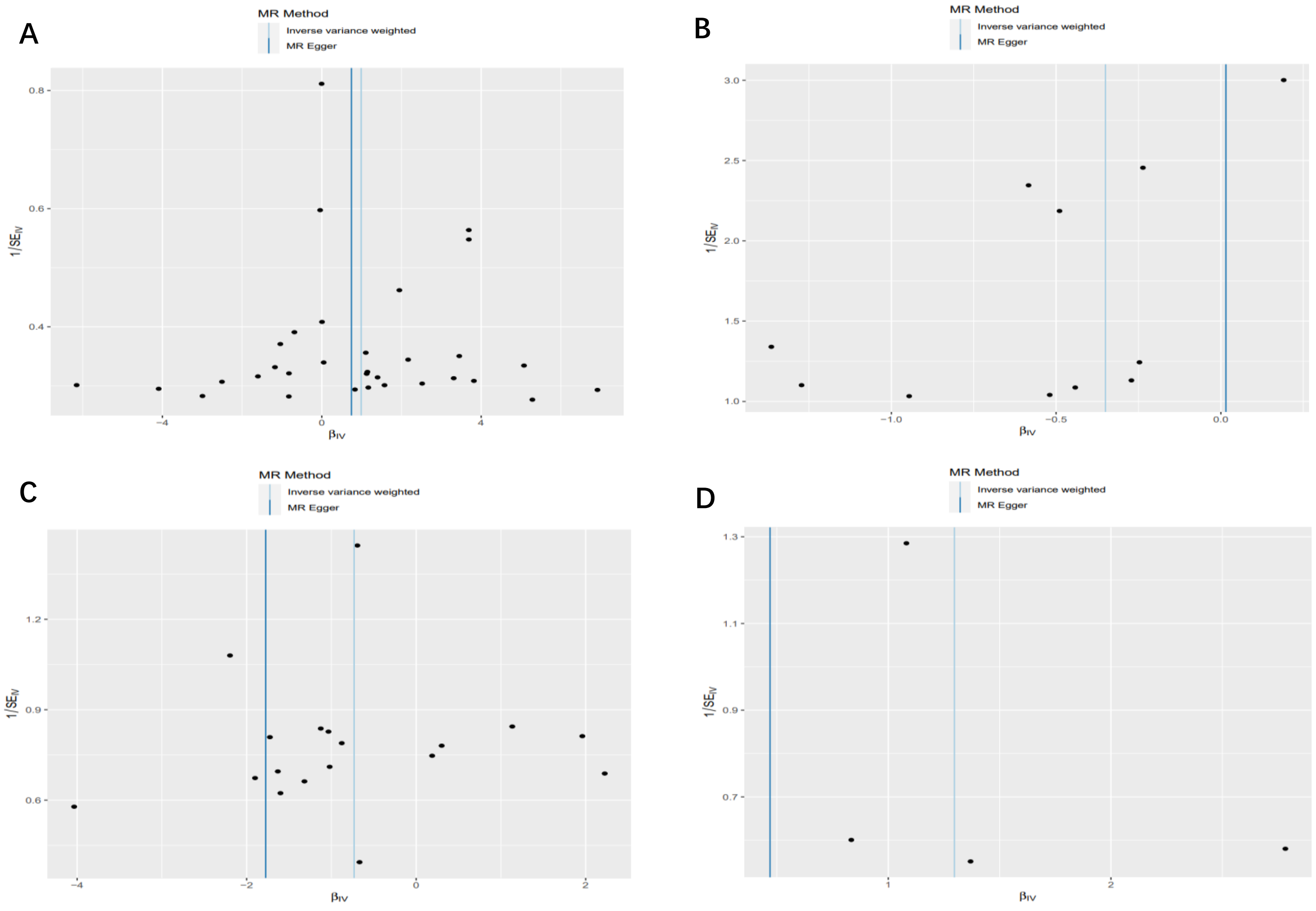
A
MR Method
Inverse variance weighted
MR Egger
0.8
0.6
0.4
1/SE$_{IV}$
−4
0
4
β$_{IV}$
B
MR Method
Inverse variance weighted
MR Egger
3.0
2.5
2.0
1.5
1.0
1/SE$_{IV}$
−1.0
−0.5
0.0
β$_{IV}$
C
MR Method
Inverse variance weighted
MR Egger
1.2
0.9
0.6
1/SE$_{IV}$
−4
−2
0
2
β$_{IV}$
D
MR Method
Inverse variance weighted
MR Egger
1.3
1.1
0.9
0.7
1/SE$_{IV}$
1
2
β$_{IV}$

E

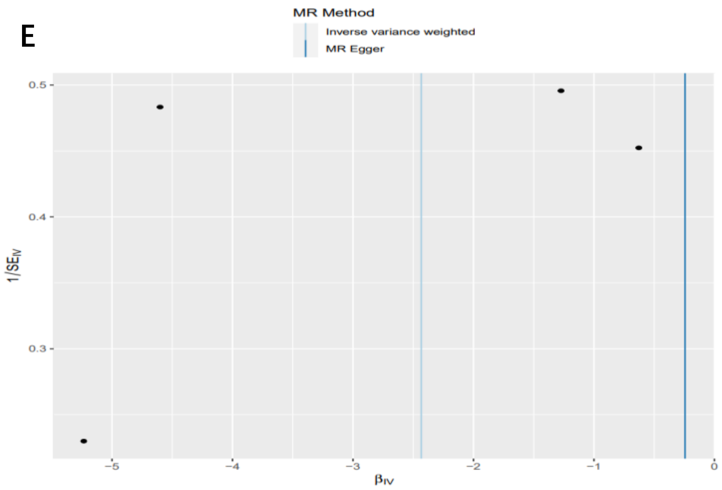

Figure S7 Funnel plots for the five potential metabolites on Graves' disease. ((A) Kynurenine, (B) Taurochenodeoxycholate, (C) 4-androsten-3beta,17beta-diol disulfate 2*, (D) Phenylalanylphenylalanine, (E) Phosphate

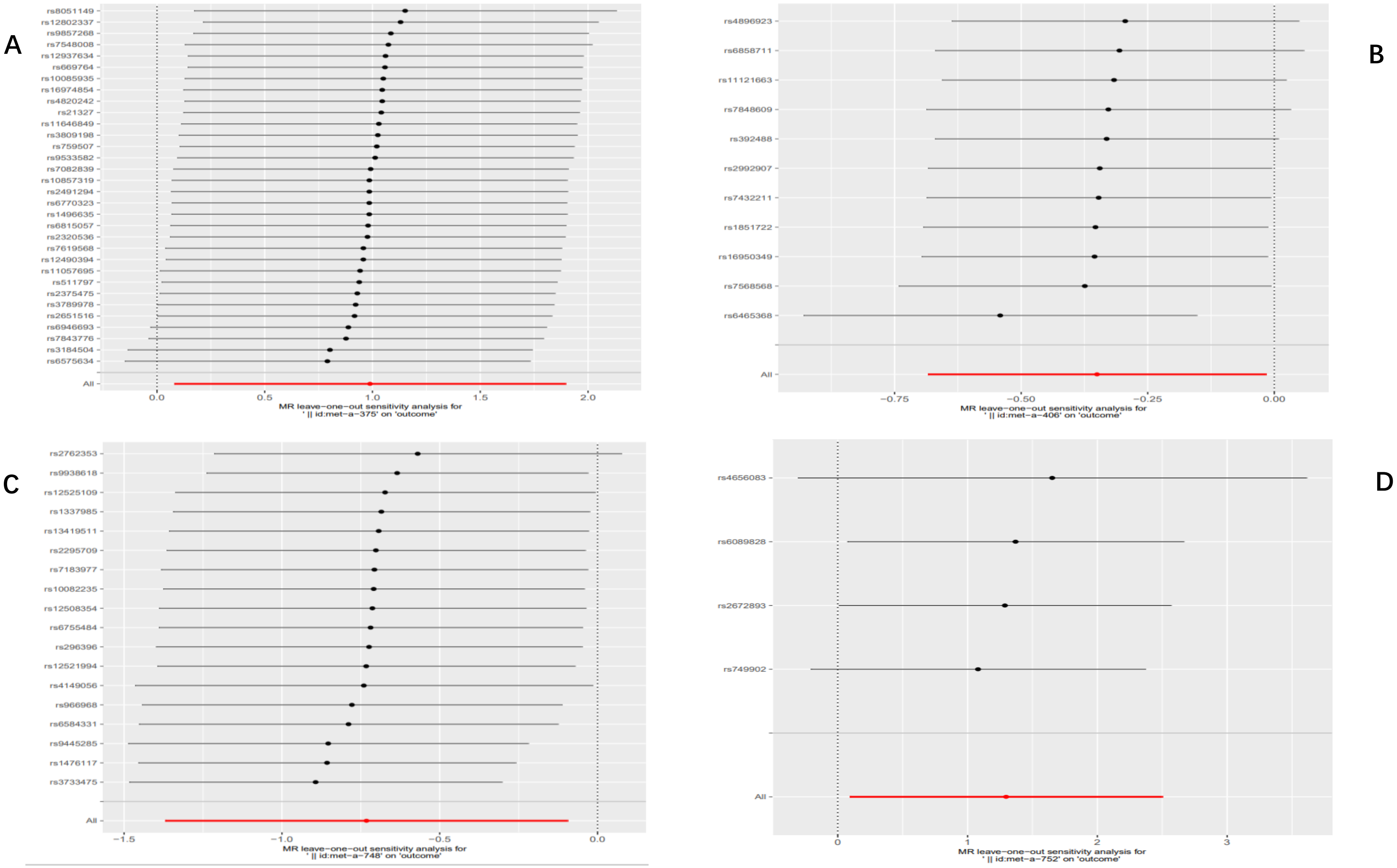
A
rs8051149
rs12802337
rs9857268
rs7548008
rs12937634
rs669764
rs10085935
rs16974854
rs4820242
rs21327
rs11646849
rs3809198
rs759507
rs9533582
rs7082839
rs10857319
rs2491294
rs6770323
rs1496635
rs6815057
rs2320536
rs7619568
rs12490394
rs11057695
rs511797
rs2375475
rs3789978
rs2651516
rs6946693
rs7843776
rs3184504
rs6575634
All
0.0
0.5
1.0
1.5
2.0
MR leave−one−out sensitivity analysis for
' || id:met−a−375' on 'outcome'
B
rs4896923
rs6858711
rs11121663
rs7848609
rs392488
rs2992907
rs7432211
rs1851722
rs16950349
rs7568568
rs6465368
All
−0.75
−0.50
−0.25
0.00
MR leave−one−out sensitivity analysis for
' || id:met−a−406' on 'outcome'
C
rs2762353
rs9938618
rs12525109
rs1337985
rs13419511
rs2295709
rs7183977
rs10082235
rs12508354
rs6755484
rs296396
rs12521994
rs4149056
rs966968
rs6584331
rs9445285
rs1476117
rs3733475
All
−1.5
−1.0
−0.5
0.0
MR leave−one−out sensitivity analysis for
' || id:met−a−748' on 'outcome'
D
rs4656083
rs6089828
rs2672893
rs749902
All
0
1
2
3
MR leave−one−out sensitivity analysis for
' || id:met−a−752' on 'outcome'

E

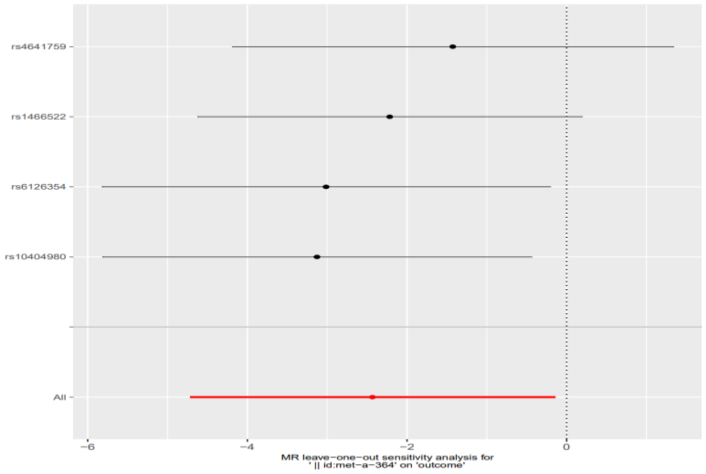

Figure S8 Leave-one-out plots for the five potential metabolites on Graves' disease. (A) Kynurenine, (B) Taurochenodeoxycholate, (C) 4-androsten-3beta,17beta-diol disulfate 2*, (D) Phenylalanylphenylalanine, (E) Phosphate

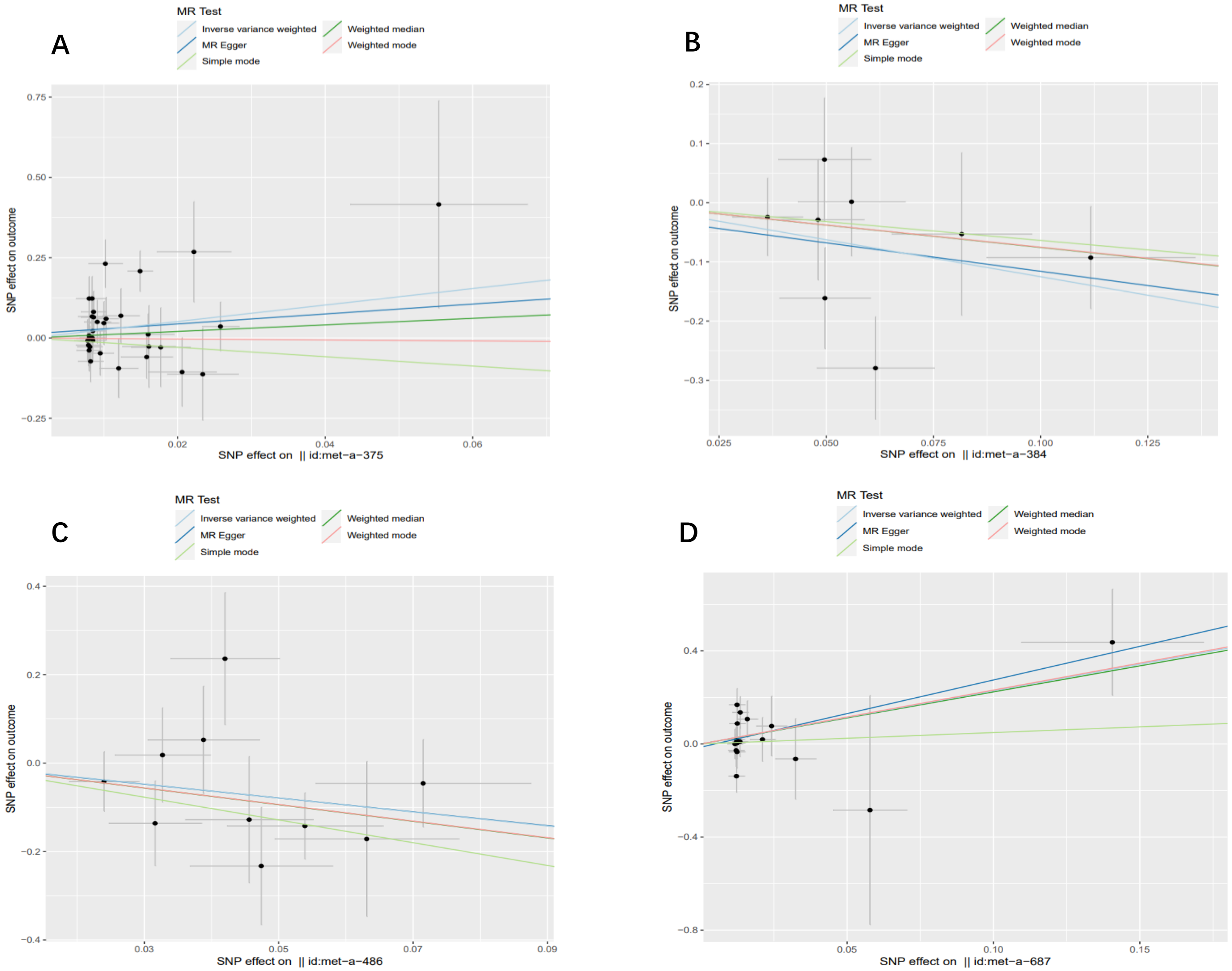
A
MR Test
Inverse variance weighted
MR Egger
Simple mode
Weighted median
Weighted mode
SNP effect on outcome
SNP effect on || id:met-a-375
B
MR Test
Inverse variance weighted
MR Egger
Simple mode
Weighted median
Weighted mode
SNP effect on outcome
SNP effect on || id:met-a-384
C
MR Test
Inverse variance weighted
MR Egger
Simple mode
Weighted median
Weighted mode
SNP effect on outcome
SNP effect on || id:met-a-486
D
MR Test
Inverse variance weighted
MR Egger
Simple mode
Weighted median
Weighted mode
SNP effect on outcome
SNP effect on || id:met-a-687

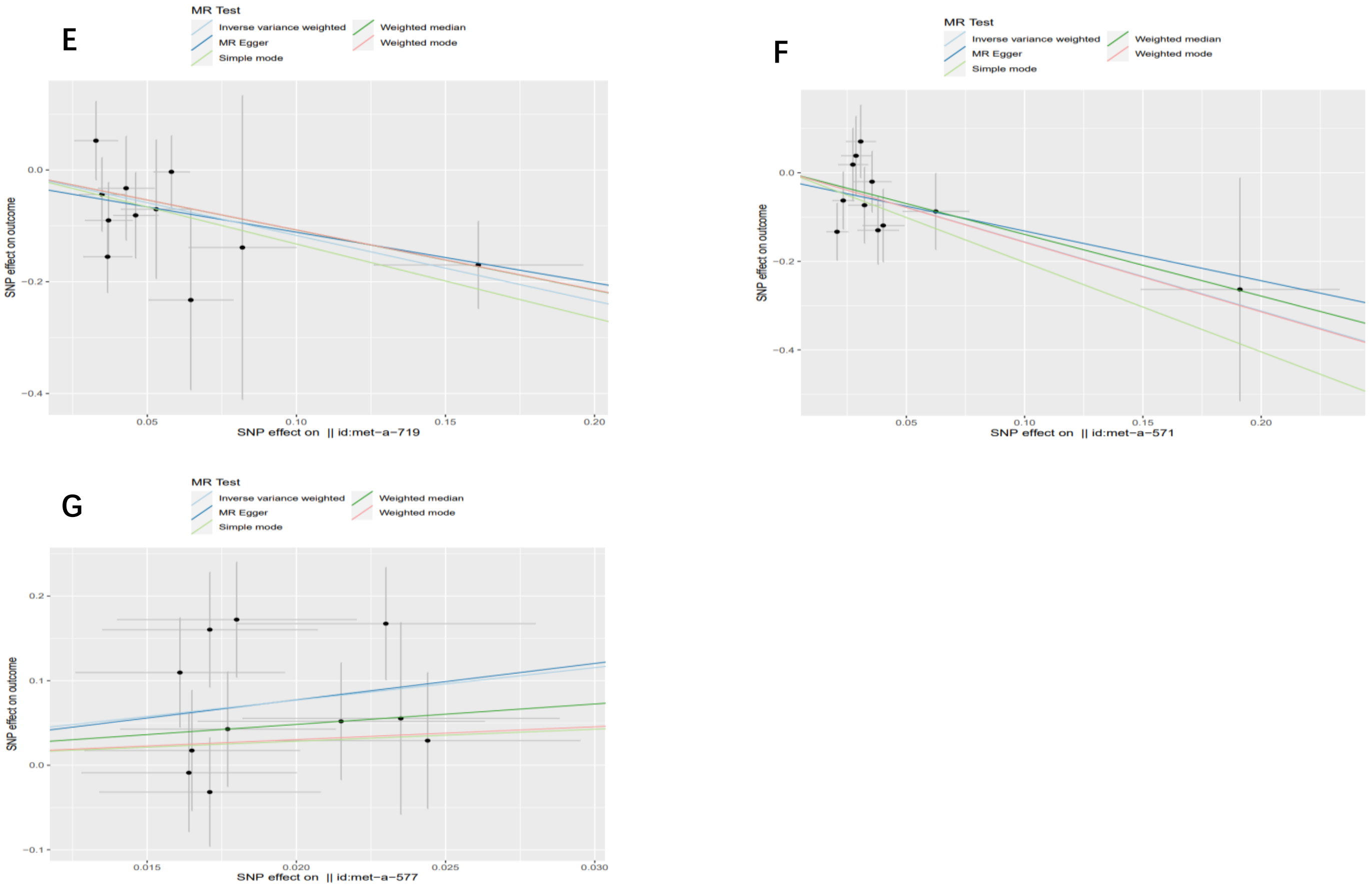

Figure S9 Scatter plots showing the causal association of seven metabolites on the risk of Hashimoto thyroiditis. (A) Kynurenine, (B) 3-methylhistidine, (C) Phenol sulfate, (D) 2-palmitoylglycerophosphocholine*, (E) X-14189—leucylalanine, (F) Gamma-tocopherol, (G) Alpha-ketoglutarate

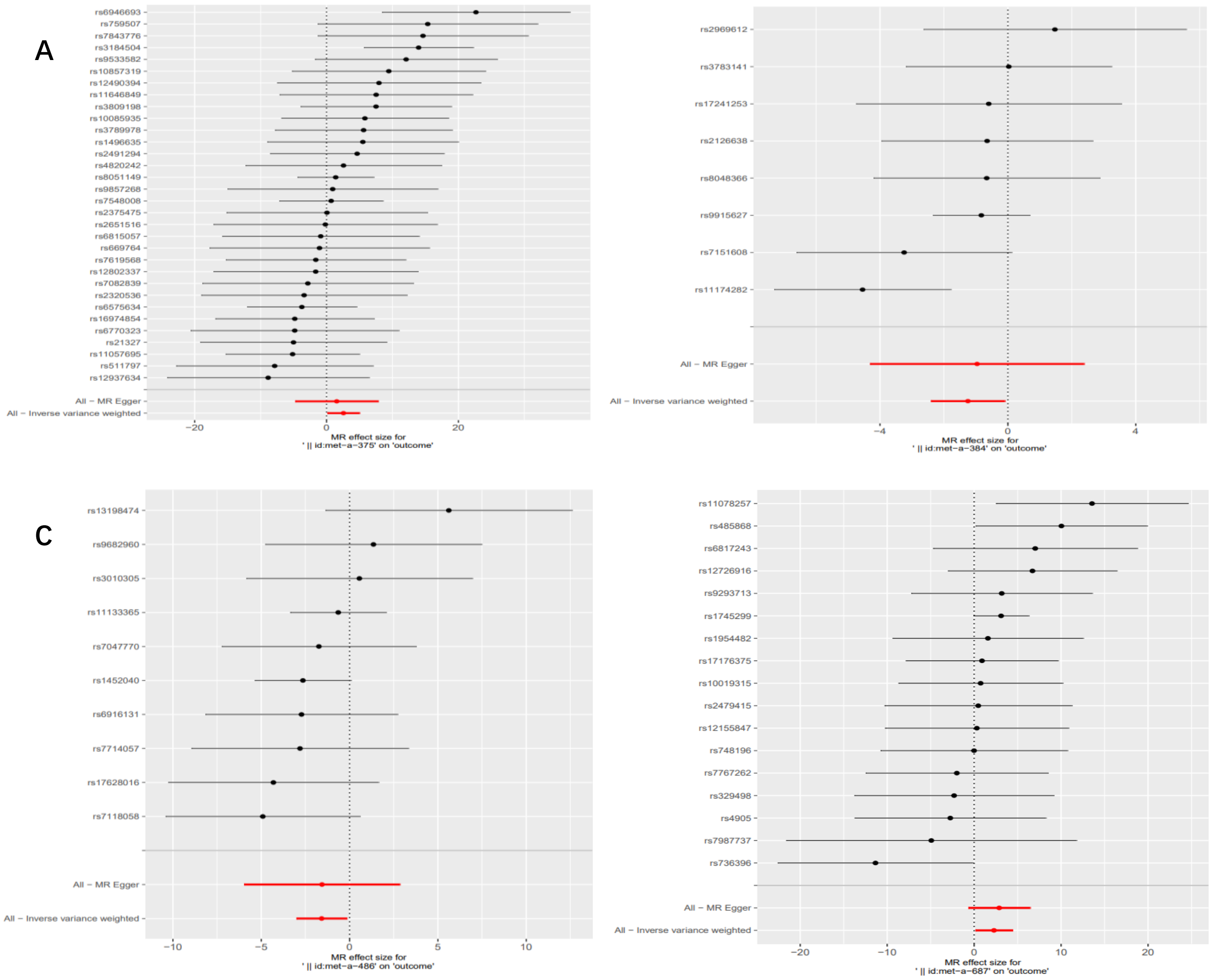
A
rs6946693
rs759507
rs7843776
rs3184504
rs9533582
rs10857319
rs12490394
rs11646849
rs3809198
rs10085935
rs3789978
rs1496635
rs2491294
rs4820242
rs8051149
rs9857268
rs7548008
rs2375475
rs2651516
rs6815057
rs669764
rs7619568
rs12802337
rs7082839
rs2320536
rs6575634
rs16974854
rs6770323
rs21327
rs11057695
rs511797
rs12937634
All − MR Egger
All − Inverse variance weighted
−20
0
20
MR effect size for
' || id:met−a−375' on 'outcome'
B
rs2969612
rs3783141
rs17241253
rs2126638
rs8048366
rs9915627
rs7151608
rs11174282
All − MR Egger
All − Inverse variance weighted
−4
0
4
MR effect size for
' || id:met−a−384' on 'outcome'
C
rs13198474
rs9682960
rs3010305
rs11133365
rs7047770
rs1452040
rs6916131
rs7714057
rs17628016
rs7118058
All − MR Egger
All − Inverse variance weighted
−10
−5
0
5
10
MR effect size for
' || id:met−a−486' on 'outcome'
D
rs11078257
rs485868
rs6817243
rs12726916
rs9293713
rs1745299
rs1954482
rs17176375
rs10019315
rs2479415
rs12155847
rs748196
rs7767262
rs329498
rs4905
rs7987737
rs736396
All − MR Egger
All − Inverse variance weighted
−20
−10
0
10
20
MR effect size for
' || id:met−a−687' on 'outcome'

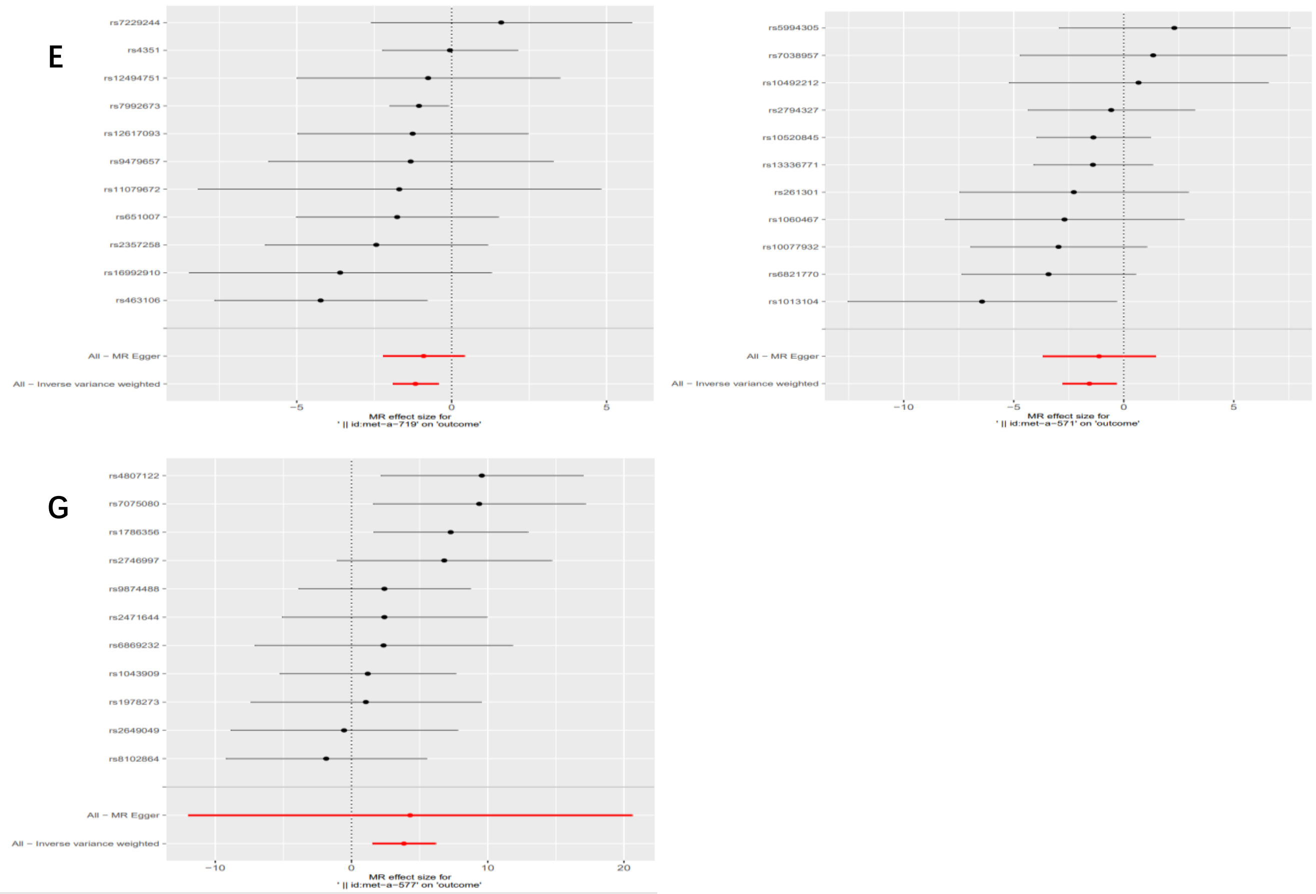

Figure S10 Forest plots for the seven potential metabolites on Hashimoto thyroiditis. (A) Kynurenine, (B) 3-methylhistidine, (C) Phenol sulfate, (D) 2-palmitoylglycerophosphocholine*, (E) X-14189—leucylalanine, (F) Gamma-tocopherol, (G) Alpha-ketoglutarate

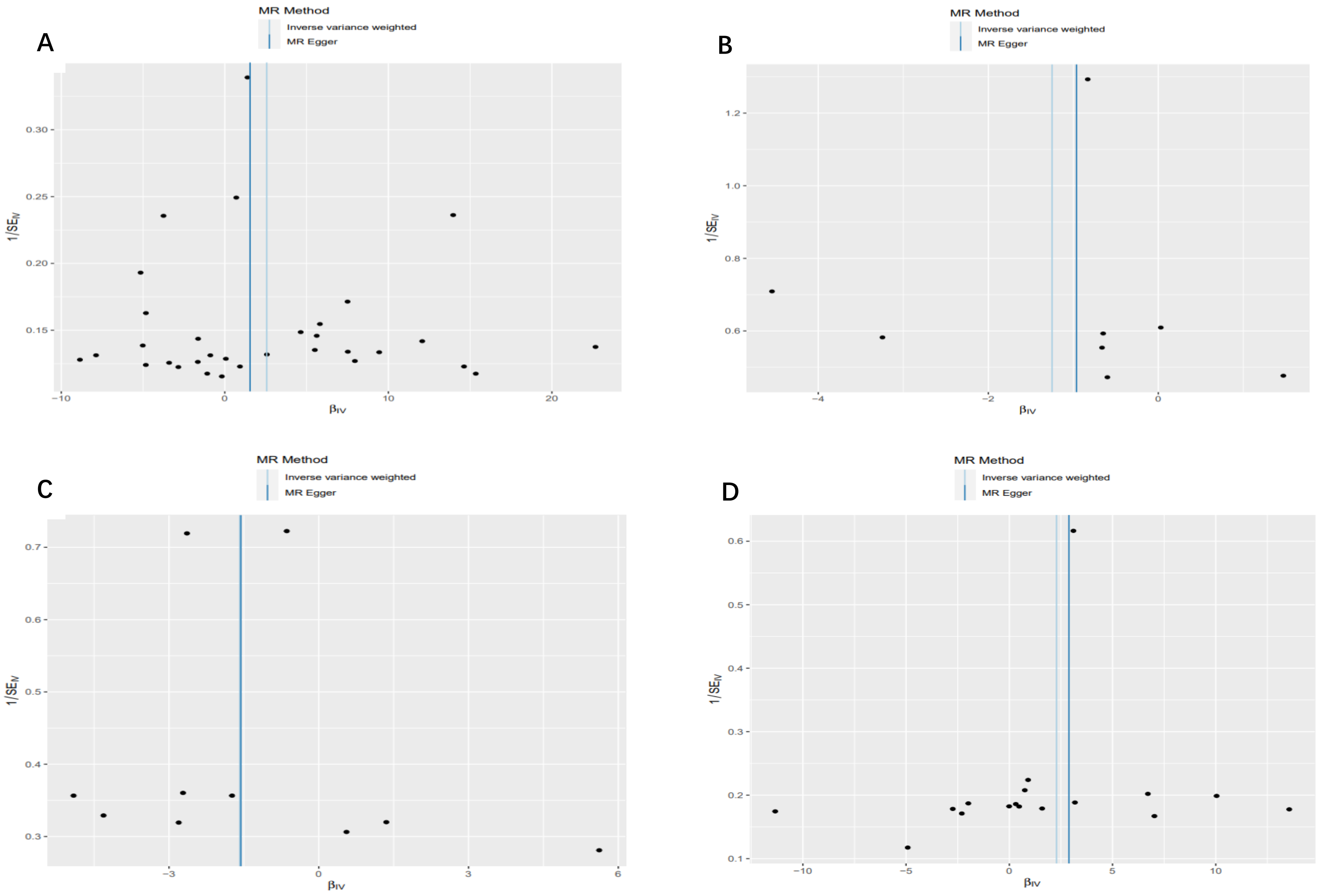

A
MR Method
Inverse variance weighted
MR Egger
1/SE_IV
0.30
0.25
0.20
0.15
β_IV
−10
0
10
20
B
MR Method
Inverse variance weighted
MR Egger
1/SE_IV
1.2
1.0
0.8
0.6
β_IV
−4
−2
0
C
MR Method
Inverse variance weighted
MR Egger
1/SE_IV
0.7
0.6
0.5
0.4
0.3
β_IV
−3
0
3
6
D
MR Method
Inverse variance weighted
MR Egger
1/SE_IV
0.6
0.5
0.4
0.3
0.2
0.1
β_IV
−10
−5
0
5
10

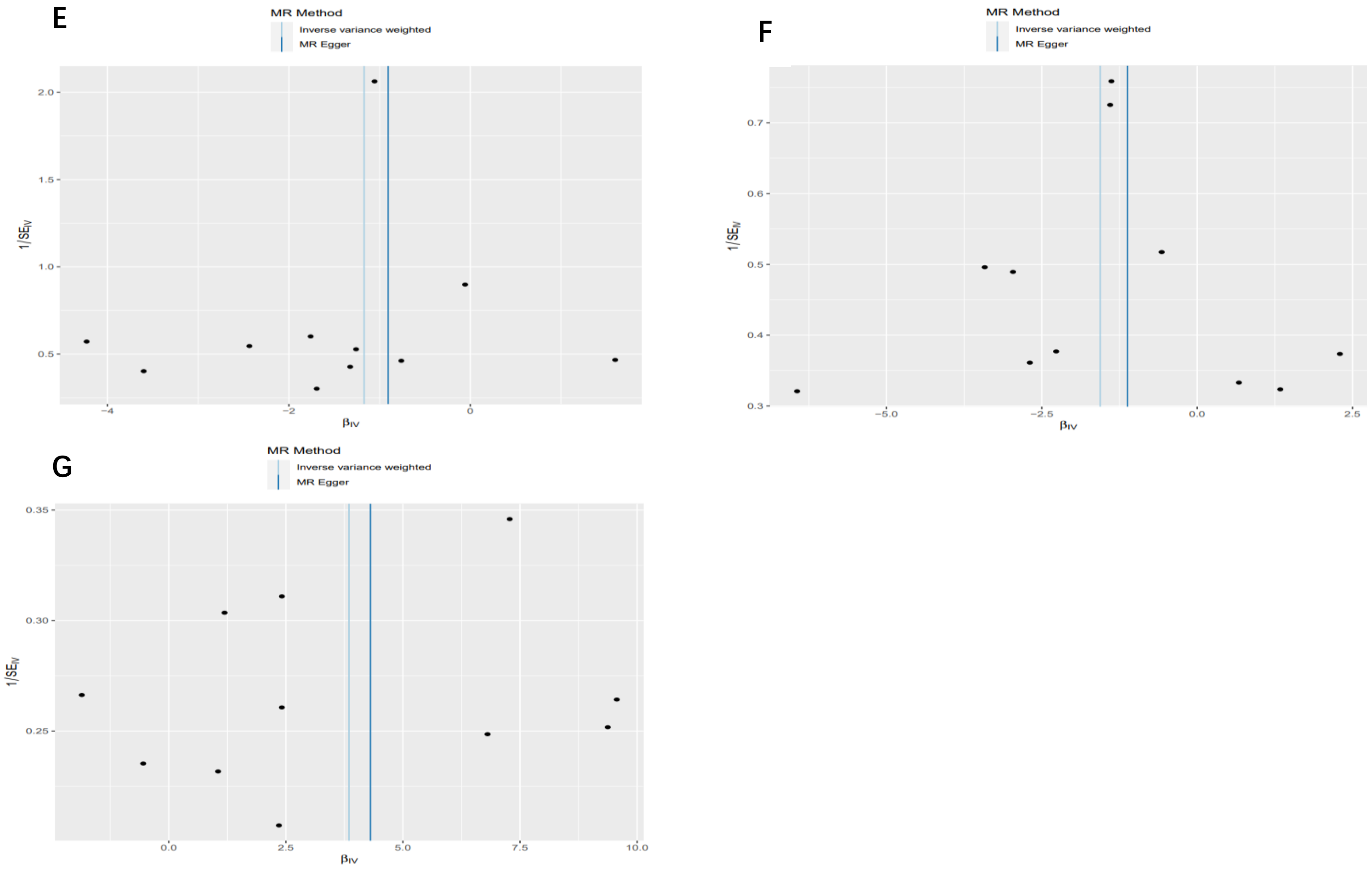

Figure S11 Funnel plots for the seven potential metabolites on Hashimoto thyroiditis. (A) Kynurenine, (B) 3-methylhistidine, (C) Phenol sulfate, (D) 2-palmitoylglycerophosphocholine*, (E) X-14189—leucylalanine, (F) Gamma-tocopherol, (G) Alpha-ketoglutarate

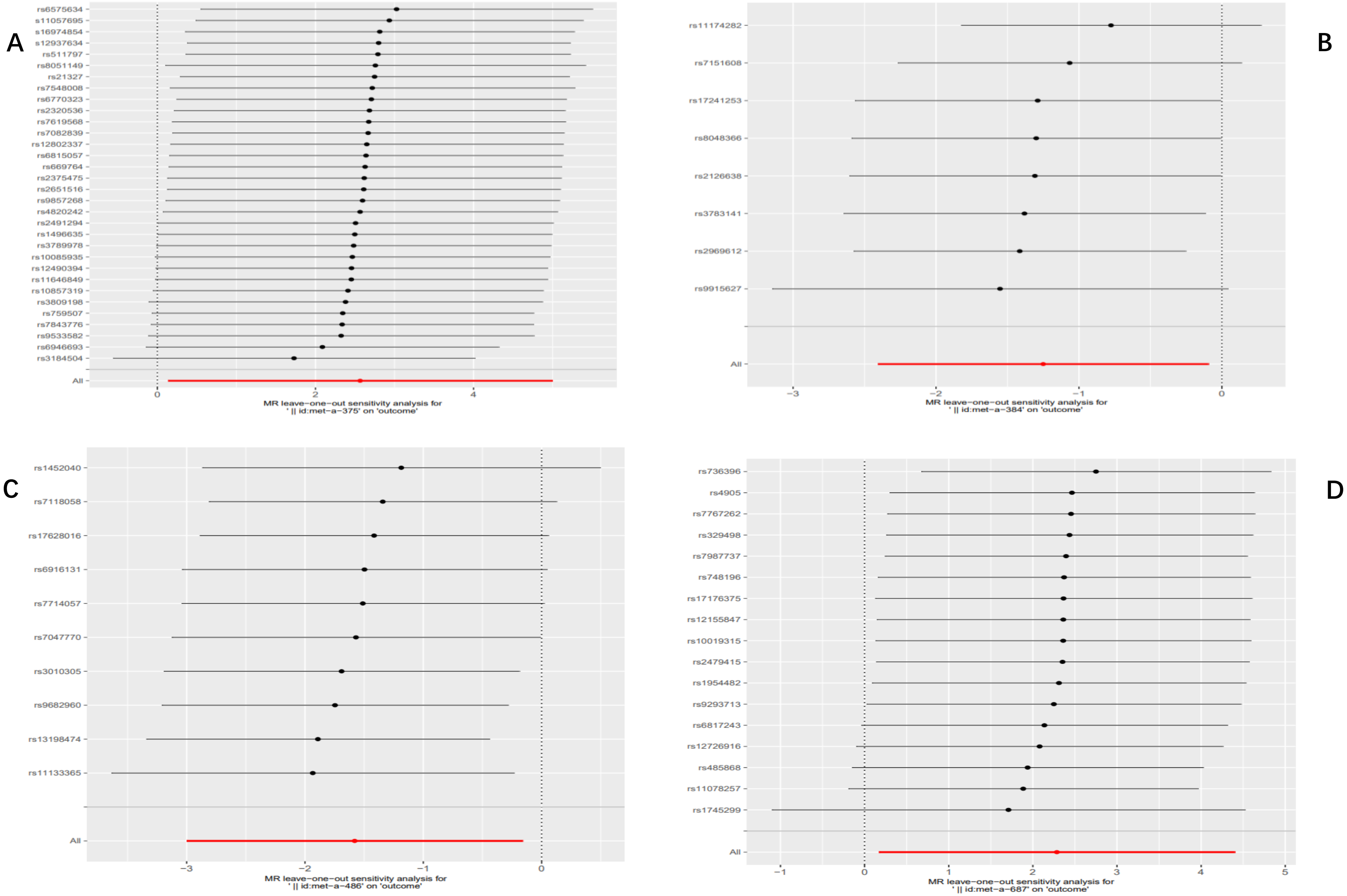

A
rs6575634
s11057695
s16974854
s12937634
rs511797
rs8051149
rs21327
rs7548008
rs6770323
rs2320536
rs7619568
rs7082839
rs12802337
rs6815057
rs669764
rs2375475
rs2651516
rs9857268
rs4820242
rs2491294
rs1496635
rs3789978
rs10085935
rs12490394
rs11646849
rs10857319
rs3809198
rs759507
rs7843776
rs9533582
rs6946693
rs3184504
All
0
2
4
MR leave−one−out sensitivity analysis for
' || id:met−a−375' on 'outcome'
B
rs11174282
rs7151608
rs17241253
rs8048366
rs2126638
rs3783141
rs2969612
rs9915627
All
−3
−2
−1
0
MR leave−one−out sensitivity analysis for
' || id:met−a−384' on 'outcome'
C
rs1452040
rs7118058
rs17628016
rs6916131
rs7714057
rs7047770
rs3010305
rs9682960
rs13198474
rs11133365
All
−3
−2
−1
0
MR leave−one−out sensitivity analysis for
' || id:met−a−486' on 'outcome'
D
rs736396
rs4905
rs7767262
rs329498
rs7987737
rs748196
rs17176375
rs12155847
rs10019315
rs2479415
rs1954482
rs9293713
rs6817243
rs12726916
rs485868
rs11078257
rs1745299
All
−1
0
1
2
3
4
5
MR leave−one−out sensitivity analysis for
' || id:met−a−687' on 'outcome'

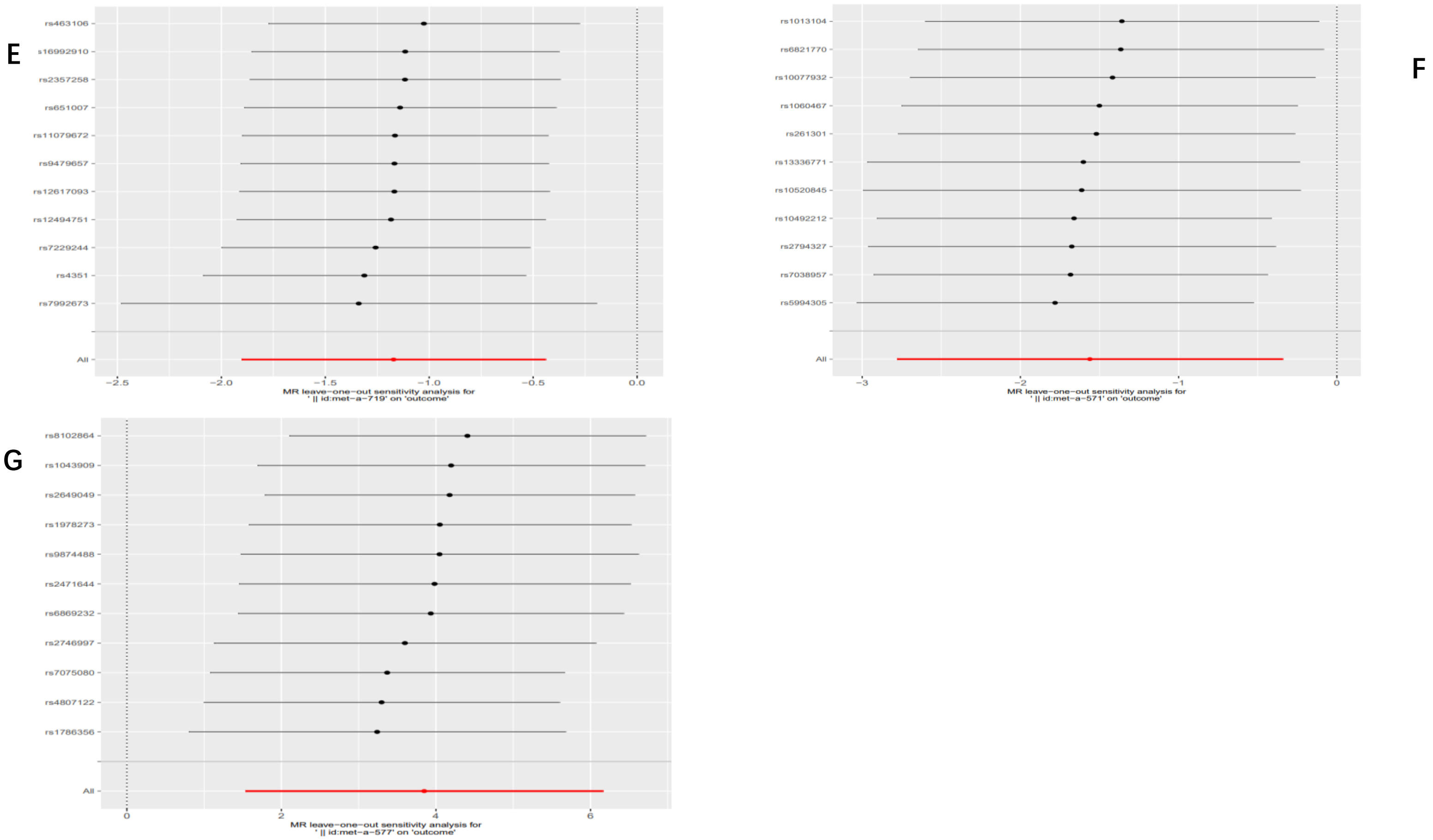

Figure S12 Leave-one-out plots for the seven potential metabolites on Hashimoto thyroiditis. (A) Kynurenine, (B) 3-methylhistidine, (C) Phenol sulfate, (D) 2-palmitoylglycerophosphocholine*, (E) X-14189—leucylalanine, (F) Gamma-tocopherol, (G) Alpha-ketoglutarate

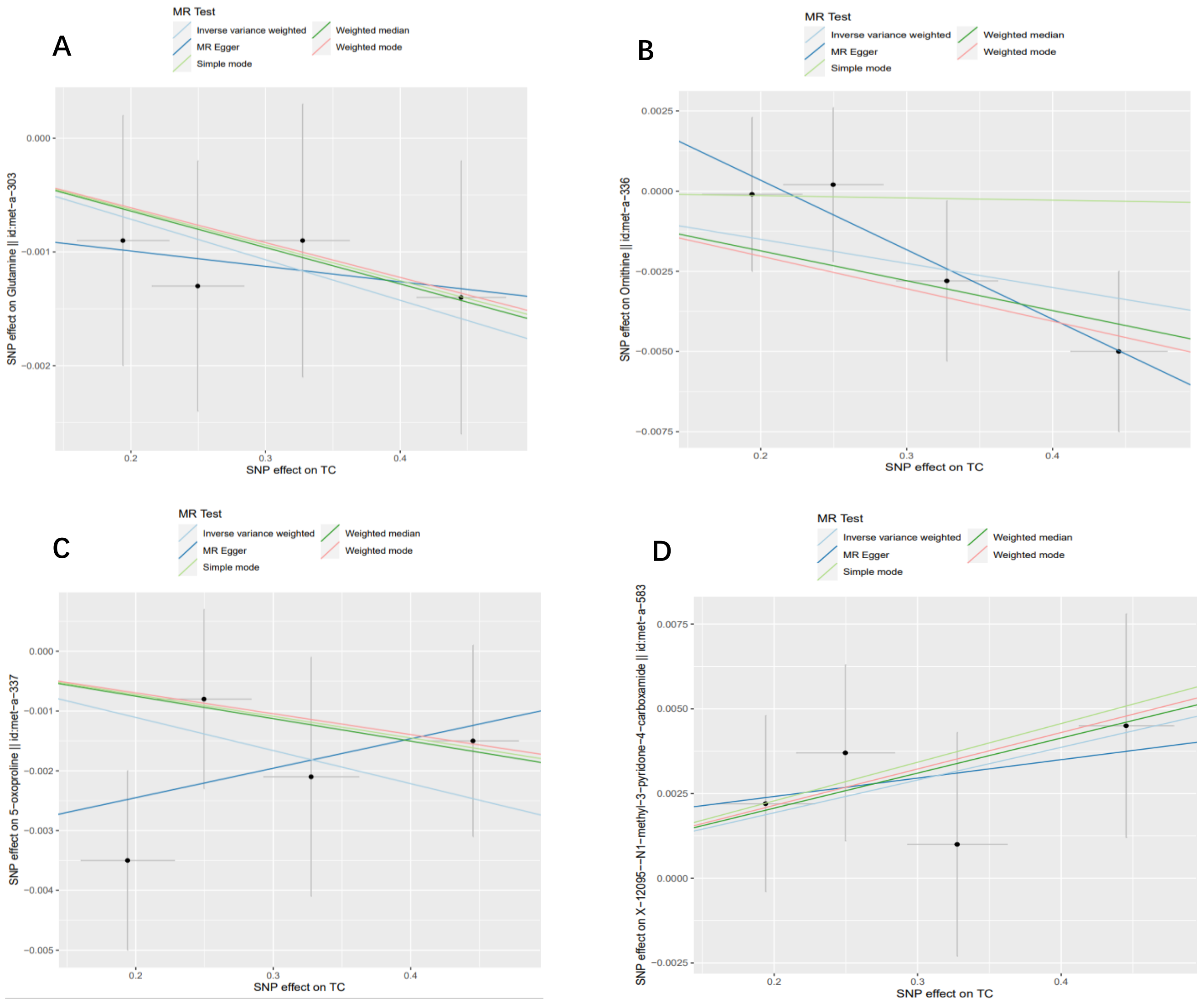
A
B
C
D
MR Test
Inverse variance weighted
MR Egger
Simple mode
Weighted median
Weighted mode
SNP effect on TC
SNP effect on Glutamine || id:met-a-303
SNP effect on Ornithine || id:met-a-336
SNP effect on 5-oxoproline || id:met-a-337
SNP effect on X-12095--N1-methyl-3-pyridone-4-carboxamide || id:met-a-583

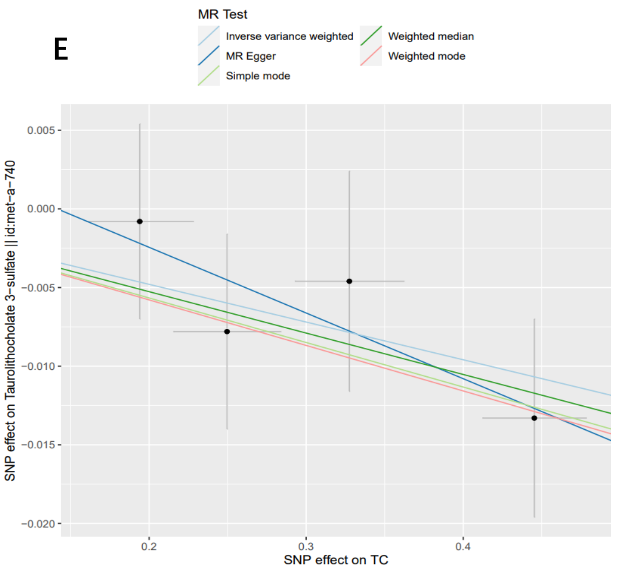

Figure S13 Scatter plots for the thyroid cancer on five potential metabolites. (A) Glutamine, (B) Ornithine, (C) 5-oxoproline, (D) X-12095--N1-methyl-3-pyridone-4-carboxamide, (E) Taurolithocholate 3-sulfate

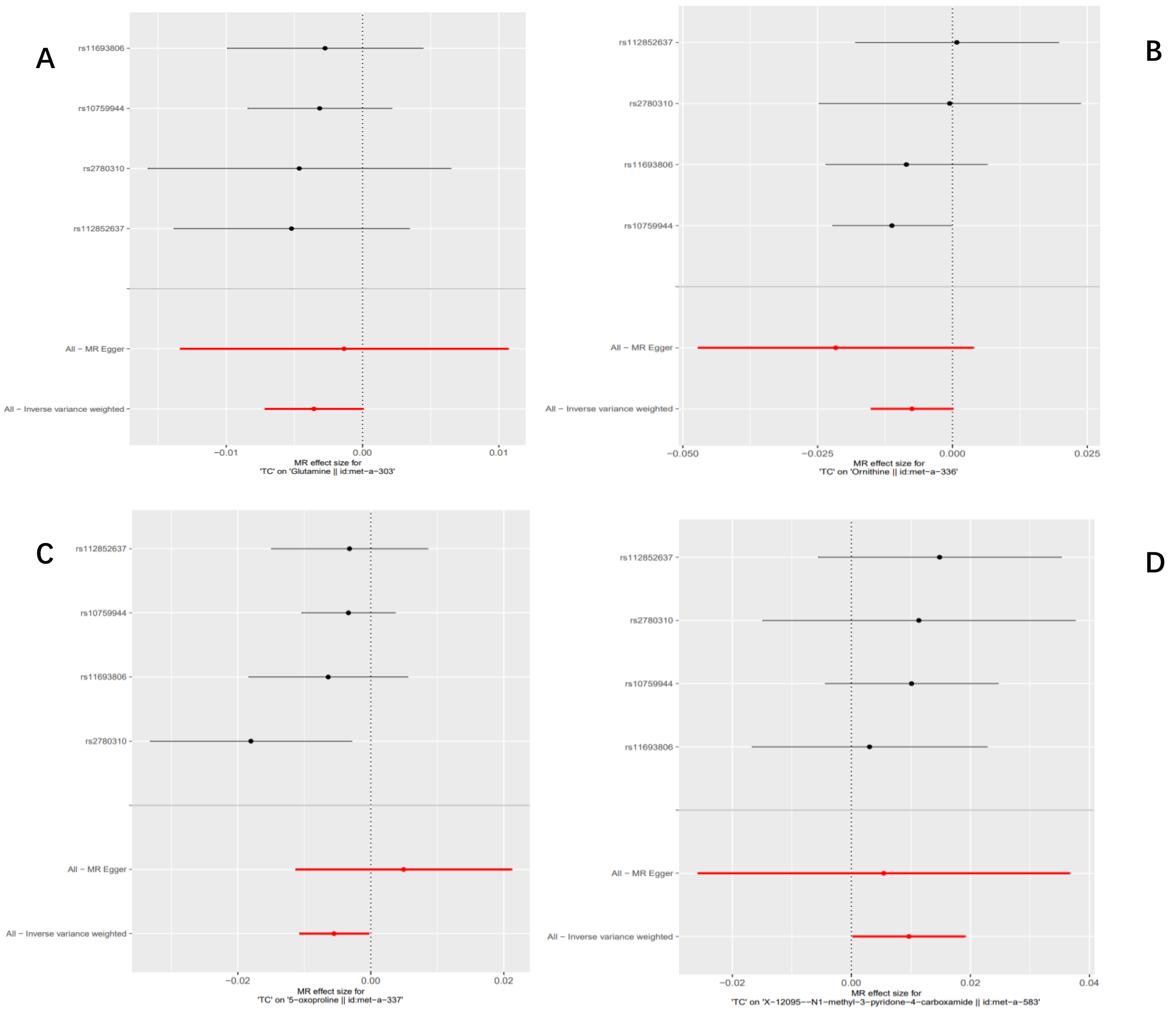
A
rs11693806
rs10759944
rs2780310
rs112852637
All − MR Egger
All − Inverse variance weighted
−0.01
0.00
0.01
MR effect size for
'TC' on 'Glutamine || id:met−a−303'
B
rs112852637
rs2780310
rs11693806
rs10759944
All − MR Egger
All − Inverse variance weighted
−0.050
−0.025
0.000
0.025
MR effect size for
'TC' on 'Ornithine || id:met−a−336'
C
rs112852637
rs10759944
rs11693806
rs2780310
All − MR Egger
All − Inverse variance weighted
−0.02
0.00
0.02
MR effect size for
'TC' on '5−oxoproline || id:met−a−337'
D
rs112852637
rs2780310
rs10759944
rs11693806
All − MR Egger
All − Inverse variance weighted
−0.02
0.00
0.02
0.04
MR effect size for
'TC' on 'X−12095−−N1−methyl−3−pyridone−4−carboxamide || id:met−a−583'

E

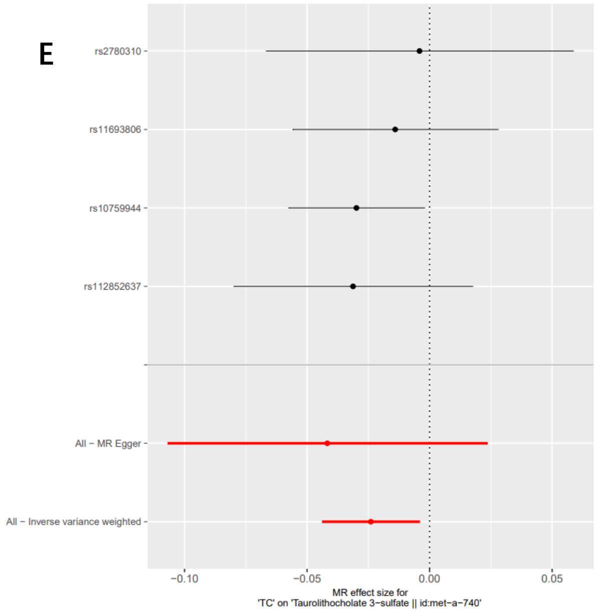

Figure S14 Forest plots for the thyroid cancer on five potential metabolites. (A) Glutamine, (B) Ornithine, (C) 5-oxoproline, (D) X-12095--N1-methyl-3-pyridone-4-carboxamide, (E) Taurolithocholate 3-sulfate

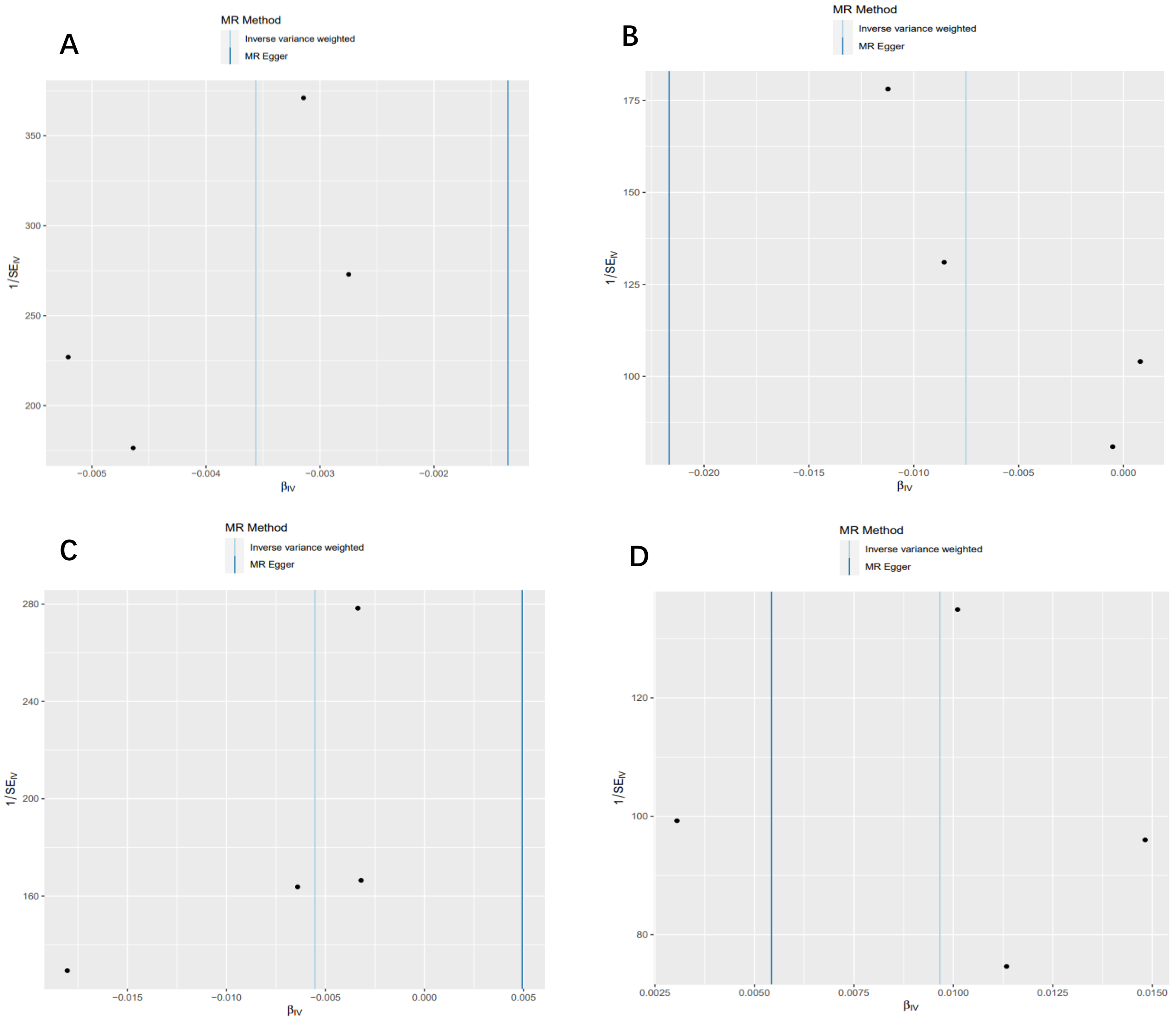
A
B
C
D
MR Method
Inverse variance weighted
MR Egger
1/SE$_{IV}$
β$_{IV}$

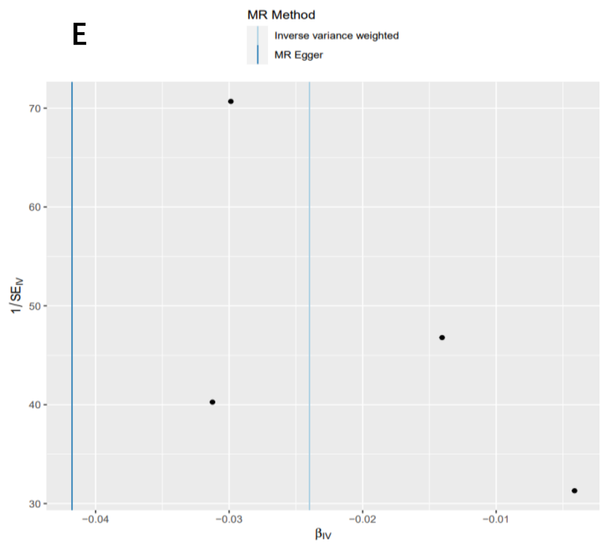

Figure S15 Funnel plots for the thyroid cancer on five potential metabolites. (A) Glutamine, (B) Ornithine, (C) 5-oxoproline, (D) X-12095--N1-methyl-3-pyridone-4-carboxamide, (E) Taurolithocholate 3-sulfate

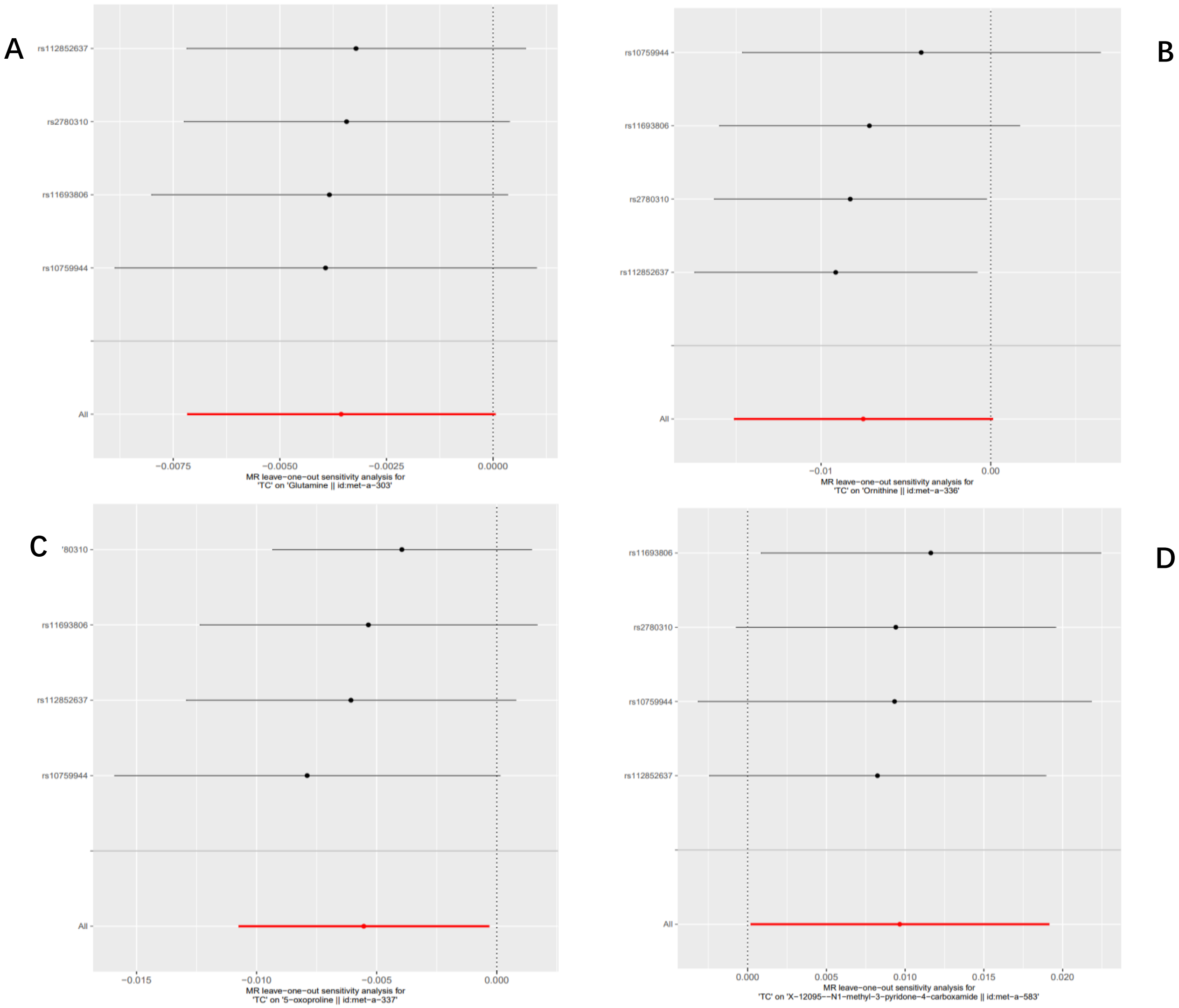
A
rs112852637
rs2780310
rs11693806
rs10759944
All
−0.0075
−0.0050
−0.0025
0.0000
MR leave−one−out sensitivity analysis for
'TC' on 'Glutamine || id:met−a−303'
B
rs10759944
rs11693806
rs2780310
rs112852637
All
−0.01
0.00
MR leave−one−out sensitivity analysis for
'TC' on 'Ornithine || id:met−a−336'
C
'80310
rs11693806
rs112852637
rs10759944
All
−0.015
−0.010
−0.005
0.000
MR leave−one−out sensitivity analysis for
'TC' on '5−oxoproline || id:met−a−337'
D
rs11693806
rs2780310
rs10759944
rs112852637
All
0.000
0.005
0.010
0.015
0.020
MR leave−one−out sensitivity analysis for
'TC' on 'X−12095−−N1−methyl−3−pyridone−4−carboxamide || id:met−a−583'

E

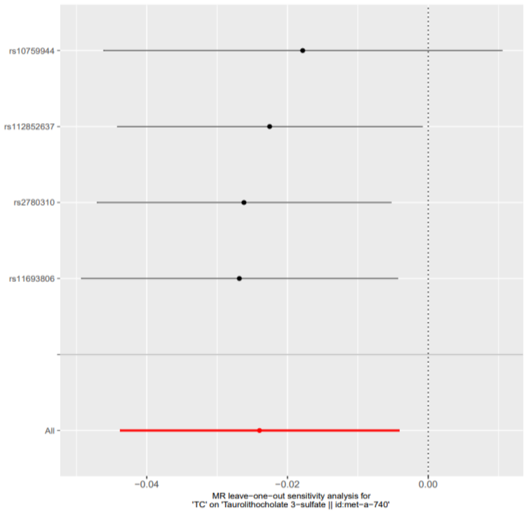

Figure S16 Leave-one-out plots for the thyroid cancer on five potential metabolites. (A) Glutamine, (B) Ornithine, (C) 5-oxoproline, (D) X-12095--N1-methyl-3-pyridone-4-carboxamide, (E) Taurolithocholate 3-sulfate

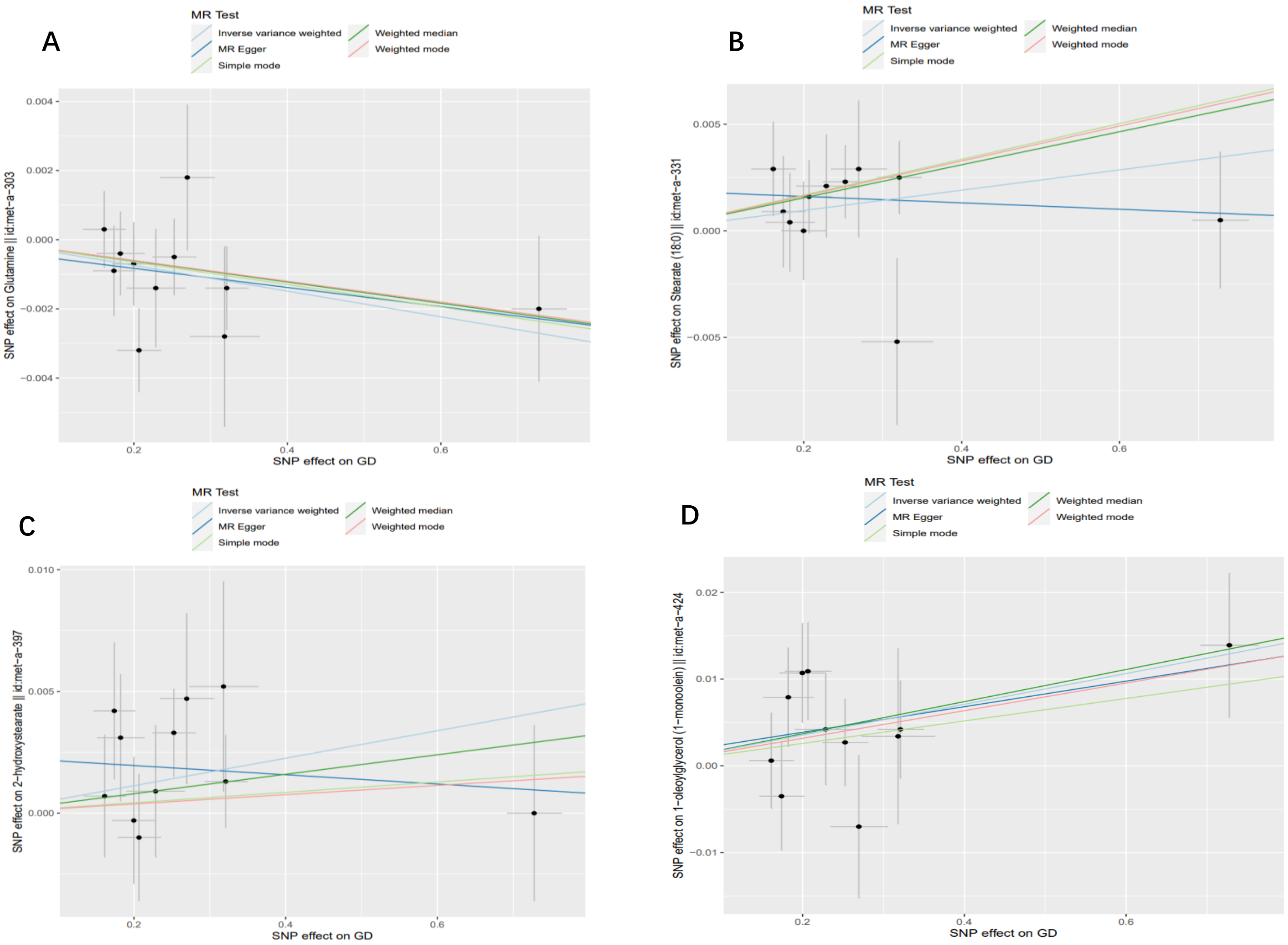
A
MR Test
Inverse variance weighted
MR Egger
Simple mode
Weighted median
Weighted mode
SNP effect on Glutamine || id:met-a-303
SNP effect on GD
B
MR Test
Inverse variance weighted
MR Egger
Simple mode
Weighted median
Weighted mode
SNP effect on Stearate (18:0) || id:met-a-331
SNP effect on GD
C
MR Test
Inverse variance weighted
MR Egger
Simple mode
Weighted median
Weighted mode
SNP effect on 2−hydroxystearate || id:met-a-397
SNP effect on GD
D
MR Test
Inverse variance weighted
MR Egger
Simple mode
Weighted median
Weighted mode
SNP effect on 1−oleoylglycerol (1−monoolein) || id:met-a-424
SNP effect on GD

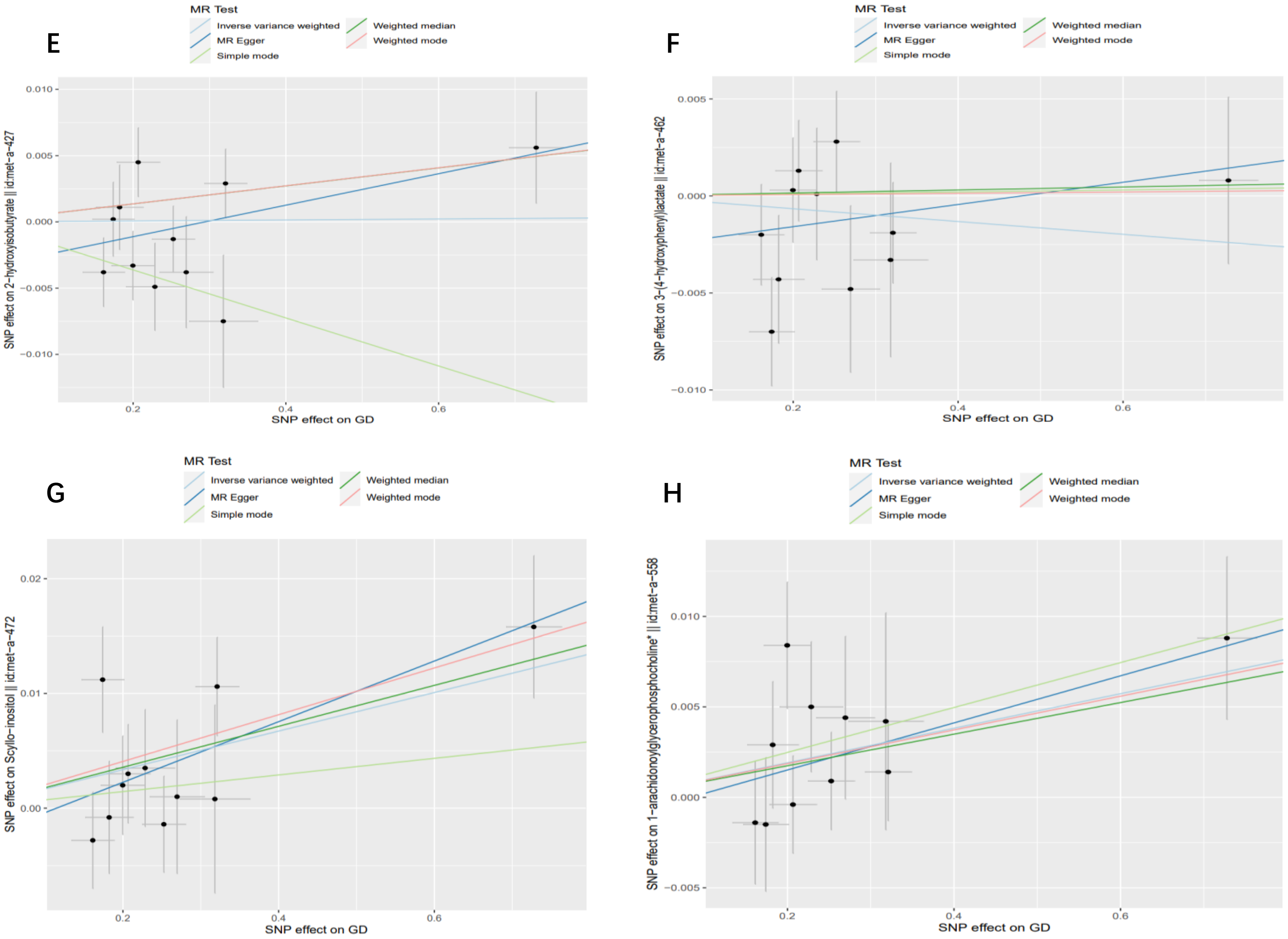
E
MR Test
Inverse variance weighted
MR Egger
Simple mode
Weighted median
Weighted mode
SNP effect on 2-hydroxyisobutyrate || id:met-a-427
SNP effect on GD
F
MR Test
Inverse variance weighted
MR Egger
Simple mode
Weighted median
Weighted mode
SNP effect on 3-(4-hydroxyphenyl)lactate || id:met-a-462
SNP effect on GD
G
MR Test
Inverse variance weighted
MR Egger
Simple mode
Weighted median
Weighted mode
SNP effect on Scyllo-inositol || id:met-a-472
SNP effect on GD
H
MR Test
Inverse variance weighted
MR Egger
Simple mode
Weighted median
Weighted mode
SNP effect on 1-arachidonoylglycerophosphocholine* || id:met-a-558
SNP effect on GD

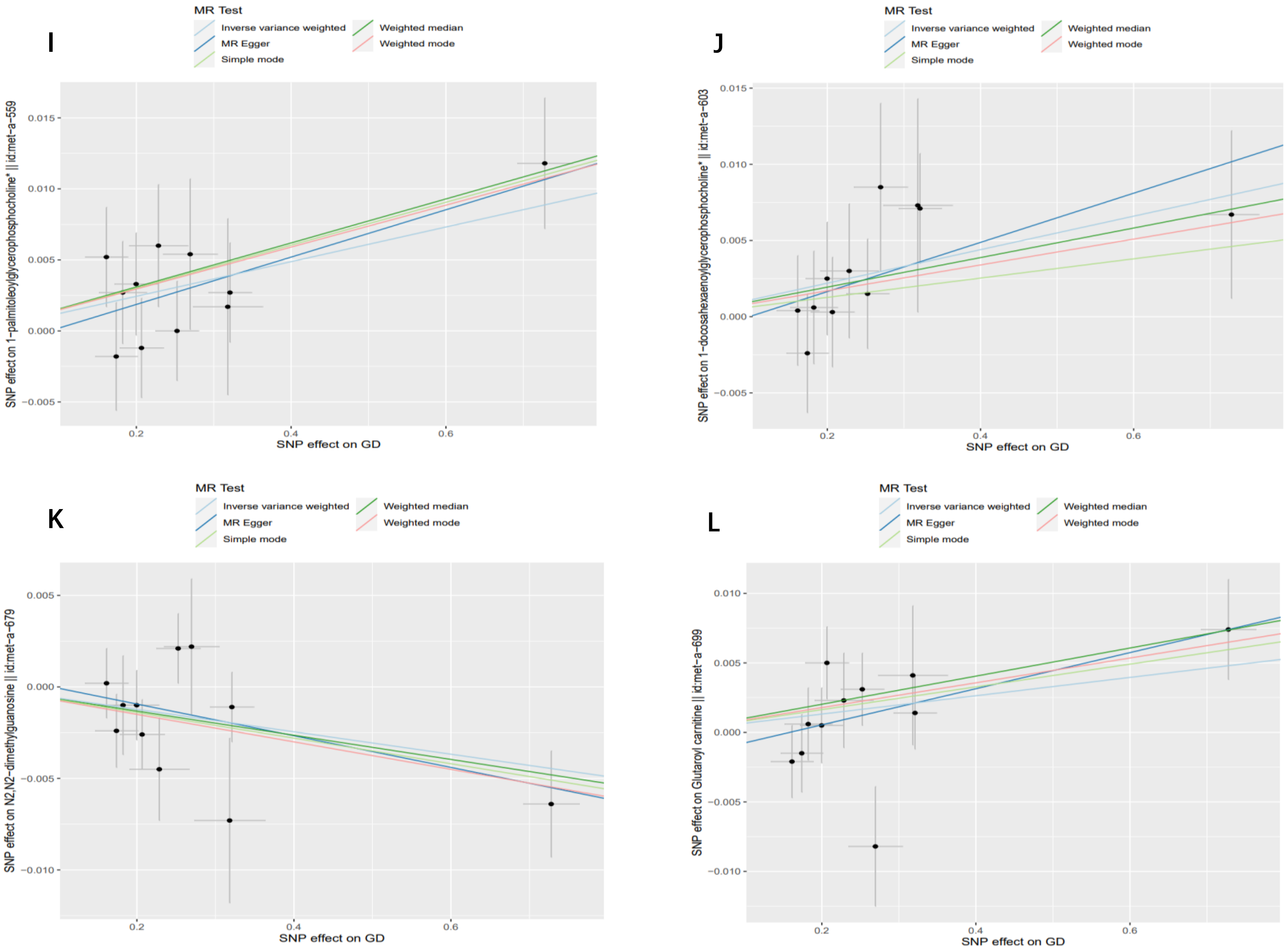
I
MR Test
Inverse variance weighted
MR Egger
Simple mode
Weighted median
Weighted mode
SNP effect on 1−palmitoleoylglycerophosphocholine* || id:met−a−559
SNP effect on GD
J
MR Test
Inverse variance weighted
MR Egger
Simple mode
Weighted median
Weighted mode
SNP effect on 1−docosahexaenoylglycerophosphocholine* || id:met−a−603
SNP effect on GD
K
MR Test
Inverse variance weighted
MR Egger
Simple mode
Weighted median
Weighted mode
SNP effect on N2,N2−dimethylguanosine || id:met−a−679
SNP effect on GD
L
MR Test
Inverse variance weighted
MR Egger
Simple mode
Weighted median
Weighted mode
SNP effect on Glutaroyl carnitine || id:met−a−699
SNP effect on GD

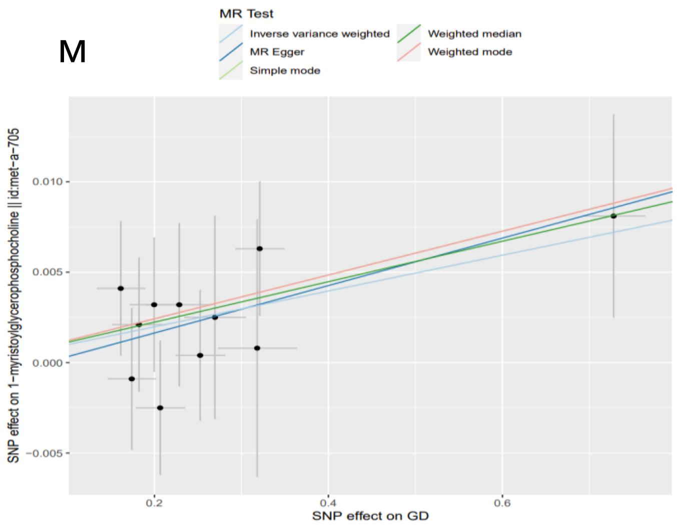

Figure S17 Scatter plots for the Graves' disease on thirteen potential metabolites. (A) Glutamine, (B) Stearate (18:0), (C) 2-hydroxystearate, (D) 1-oleoylglycerol (1-monoolein), (E) 2-hydroxyisobutyrate, (F) 3-(4-hydroxyphenyl)lactate, (G) Scyllo-inositol, (H) 1-arachidonoylglycerophosphocholine*, (I) 1-palmitoleoylglycerophosphocholine*, (J) 1-docosahexaenoylglycerophosphocholine*, (K) N2,N2-dimethylguanosine, (L) Glutaroyl carnitine, (M) 1-myristoylglycerophosphocholine

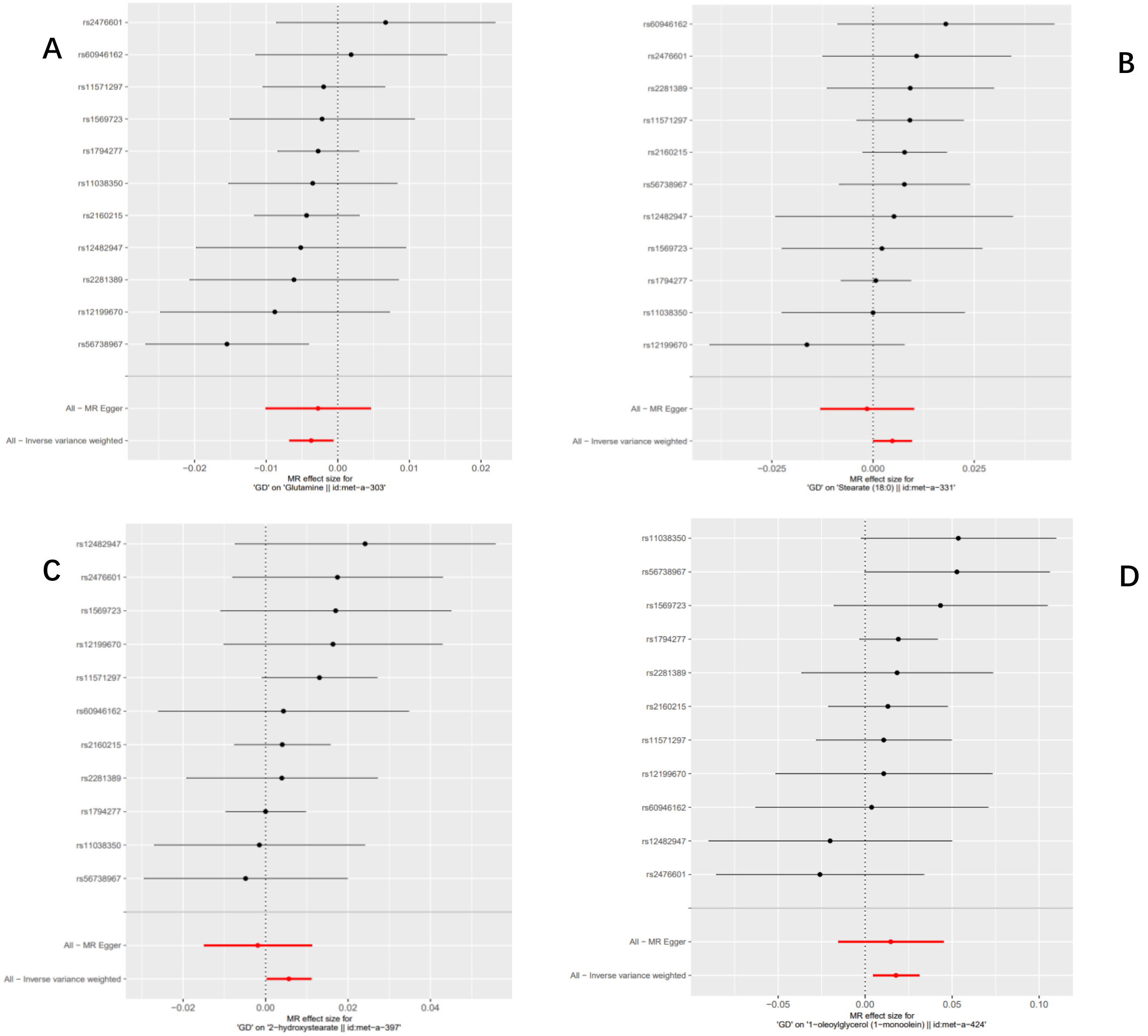
A
rs2476601
rs60946162
rs11571297
rs1569723
rs1794277
rs11038350
rs2160215
rs12482947
rs2281389
rs12199670
rs56738967
All − MR Egger
All − Inverse variance weighted
−0.02 −0.01 0.00 0.01 0.02
MR effect size for
'GD' on 'Glutamine || id:met−a−303'
B
rs60946162
rs2476601
rs2281389
rs11571297
rs2160215
rs56738967
rs12482947
rs1569723
rs1794277
rs11038350
rs12199670
All − MR Egger
All − Inverse variance weighted
−0.025 0.000 0.025
MR effect size for
'GD' on 'Stearate (18:0) || id:met−a−331'
C
rs12482947
rs2476601
rs1569723
rs12199670
rs11571297
rs60946162
rs2160215
rs2281389
rs1794277
rs11038350
rs56738967
All − MR Egger
All − Inverse variance weighted
−0.02 0.00 0.02 0.04
MR effect size for
'GD' on '2−hydroxystearate || id:met−a−397'
D
rs11038350
rs56738967
rs1569723
rs1794277
rs2281389
rs2160215
rs11571297
rs12199670
rs60946162
rs12482947
rs2476601
All − MR Egger
All − Inverse variance weighted
−0.05 0.00 0.05 0.10
MR effect size for
'GD' on '1−oleoylglycerol (1−monoolein) || id:met−a−424'

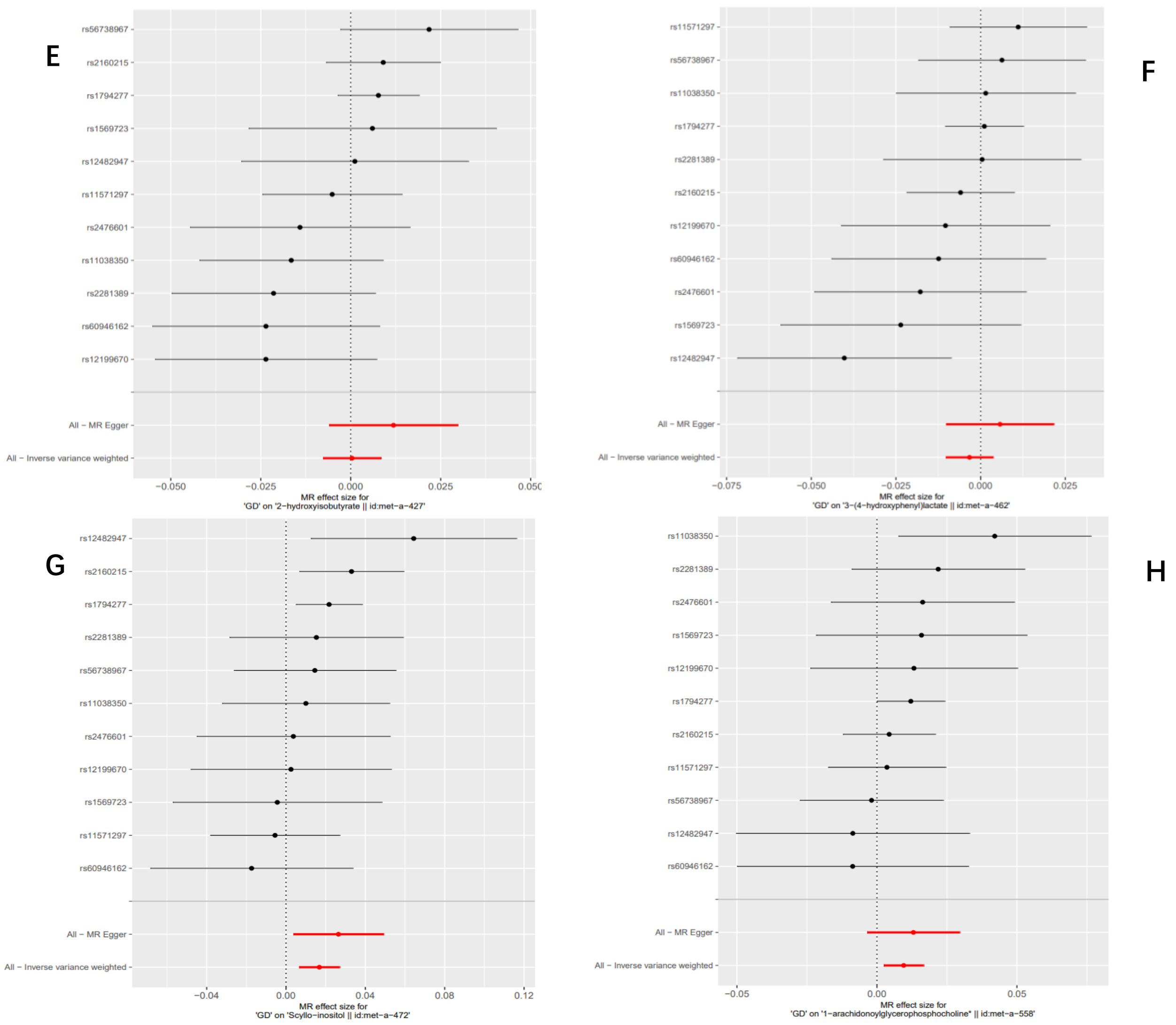
E
rs56738967
rs2160215
rs1794277
rs1569723
rs12482947
rs11571297
rs2476601
rs11038350
rs2281389
rs60946162
rs12199670
All − MR Egger
All − Inverse variance weighted
−0.050 −0.025 0.000 0.025 0.050
MR effect size for
'GD' on '2−hydroxyisobutyrate || id:met−a−427'
F
rs11571297
rs56738967
rs11038350
rs1794277
rs2281389
rs2160215
rs12199670
rs60946162
rs2476601
rs1569723
rs12482947
All − MR Egger
All − Inverse variance weighted
−0.075 −0.050 −0.025 0.000 0.025
MR effect size for
'GD' on '3−(4−hydroxyphenyl)lactate || id:met−a−462'
G
rs12482947
rs2160215
rs1794277
rs2281389
rs56738967
rs11038350
rs2476601
rs12199670
rs1569723
rs11571297
rs60946162
All − MR Egger
All − Inverse variance weighted
−0.04 0.00 0.04 0.08 0.12
MR effect size for
'GD' on 'Scyllo−inositol || id:met−a−472'
H
rs11038350
rs2281389
rs2476601
rs1569723
rs12199670
rs1794277
rs2160215
rs11571297
rs56738967
rs12482947
rs60946162
All − MR Egger
All − Inverse variance weighted
−0.05 0.00 0.05
MR effect size for
'GD' on '1−arachidonoylglycerophosphocholine* || id:met−a−558'

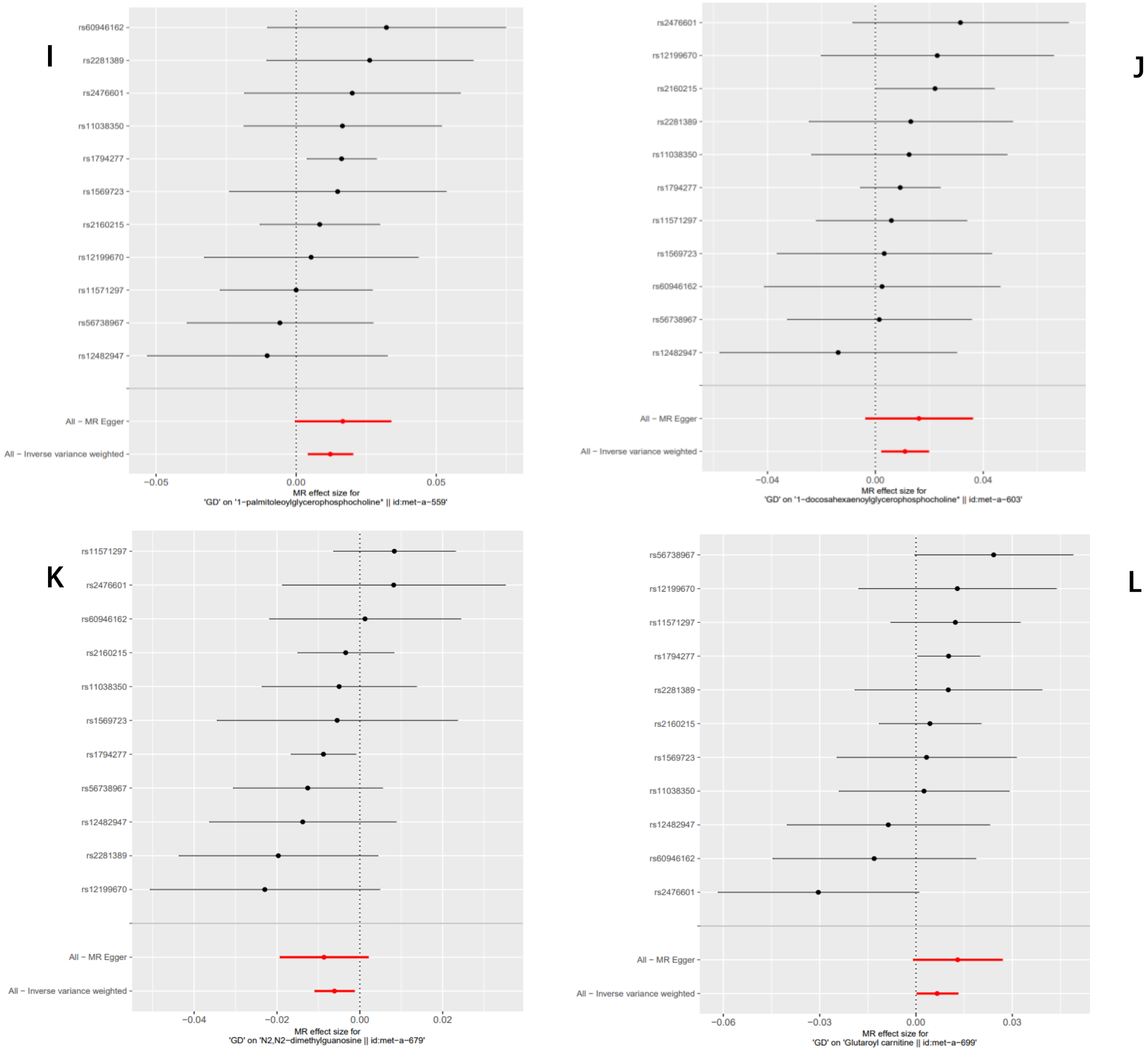
I
rs60946162
rs2281389
rs2476601
rs11038350
rs1794277
rs1569723
rs2160215
rs12199670
rs11571297
rs56738967
rs12482947
All − MR Egger
All − Inverse variance weighted
−0.05
0.00
0.05
MR effect size for
'GD' on '1−palmitoleoylglycerophosphocholine*' || id:met−a−559'
J
rs2476601
rs12199670
rs2160215
rs2281389
rs11038350
rs1794277
rs11571297
rs1569723
rs60946162
rs56738967
rs12482947
All − MR Egger
All − Inverse variance weighted
−0.04
0.00
0.04
MR effect size for
'GD' on '1−docosahexaenoylglycerophosphocholine*' || id:met−a−603'
K
rs11571297
rs2476601
rs60946162
rs2160215
rs11038350
rs1569723
rs1794277
rs56738967
rs12482947
rs2281389
rs12199670
All − MR Egger
All − Inverse variance weighted
−0.04
−0.02
0.00
0.02
MR effect size for
'GD' on 'N2,N2−dimethylguanosine || id:met−a−679'
L
rs56738967
rs12199670
rs11571297
rs1794277
rs2281389
rs2160215
rs1569723
rs11038350
rs12482947
rs60946162
rs2476601
All − MR Egger
All − Inverse variance weighted
−0.06
−0.03
0.00
0.03
MR effect size for
'GD' on 'Glutaroyl carnitine || id:met−a−699'

M

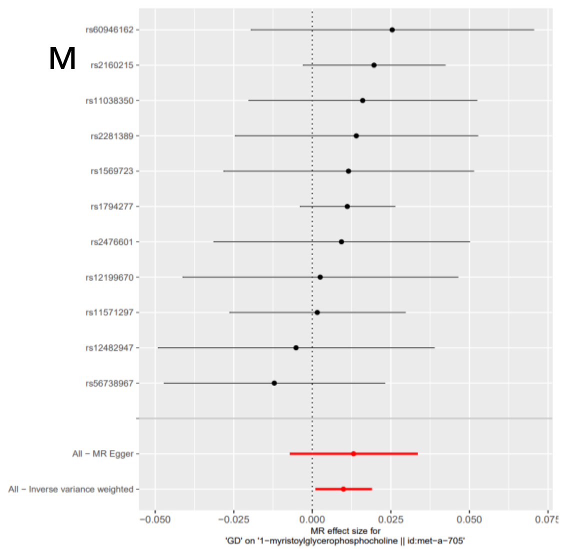

Figure S18 Forest plots for the Graves' disease on thirteen potential metabolites. (A) Glutamine, (B) Stearate (18:0), (C) 2-hydroxystearate, (D) 1-oleoylglycerol (1-monoolein), (E) 2-hydroxyisobutyrate, (F) 3-(4-hydroxyphenyl)lactate, (G) Scyllo-inositol, (H) 1-arachidonoylglycerophosphocholine*, (I) 1-palmitoleoylglycerophosphocholine*, (J) 1-docosahexaenoylglycerophosphocholine*, (K) N2,N2-dimethylguanosine, (L) Glutaroyl carnitine, (M) 1-myristoylglycerophosphocholine

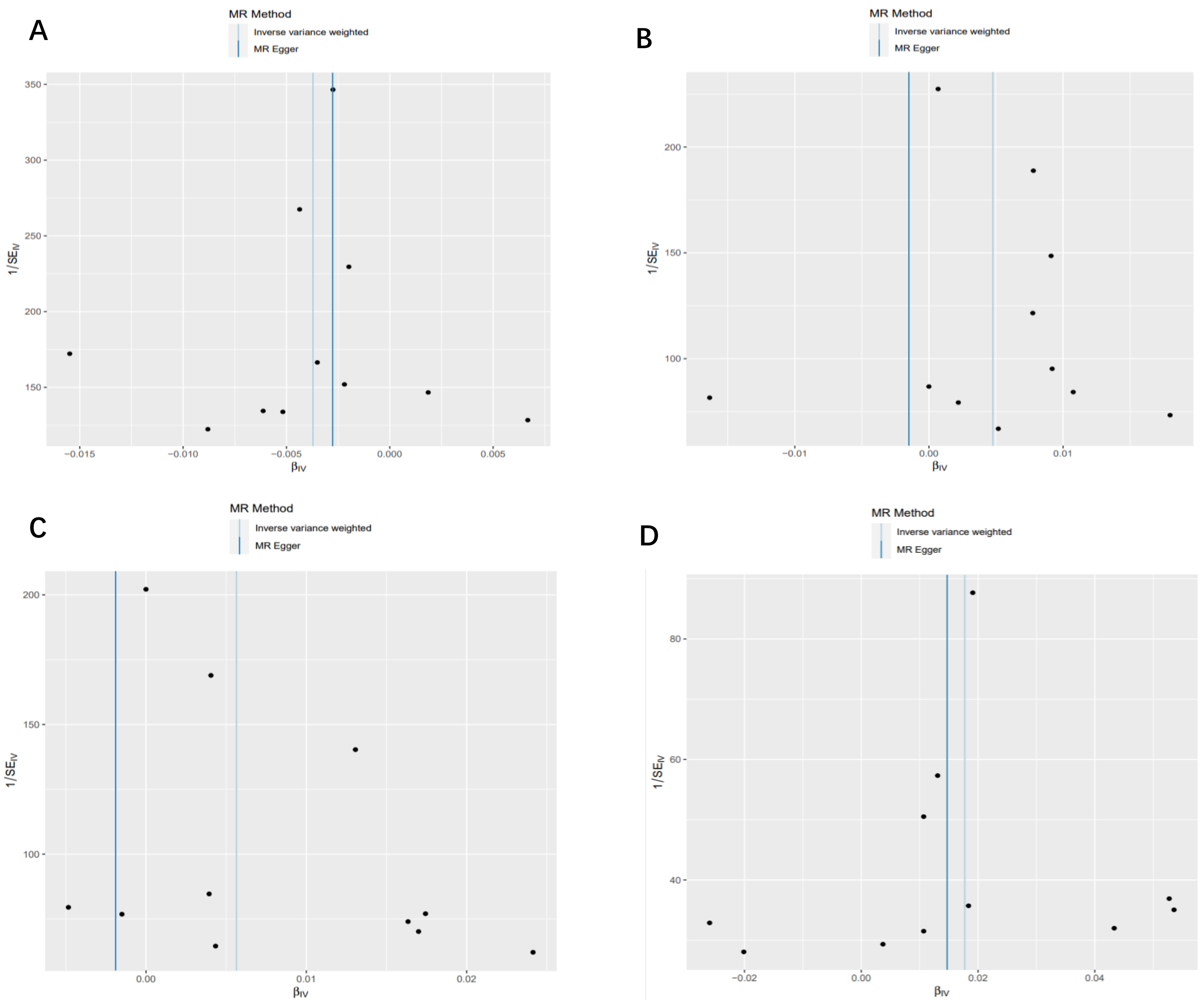
A
MR Method
Inverse variance weighted
MR Egger
350
300
250
200
150
1/SE$_{IV}$
−0.015
−0.010
−0.005
0.000
0.005
β$_{IV}$
B
MR Method
Inverse variance weighted
MR Egger
200
150
100
1/SE$_{IV}$
−0.01
0.00
0.01
β$_{IV}$
C
MR Method
Inverse variance weighted
MR Egger
200
150
100
1/SE$_{IV}$
0.00
0.01
0.02
β$_{IV}$
D
MR Method
Inverse variance weighted
MR Egger
80
60
40
1/SE$_{IV}$
−0.02
0.00
0.02
0.04
β$_{IV}$

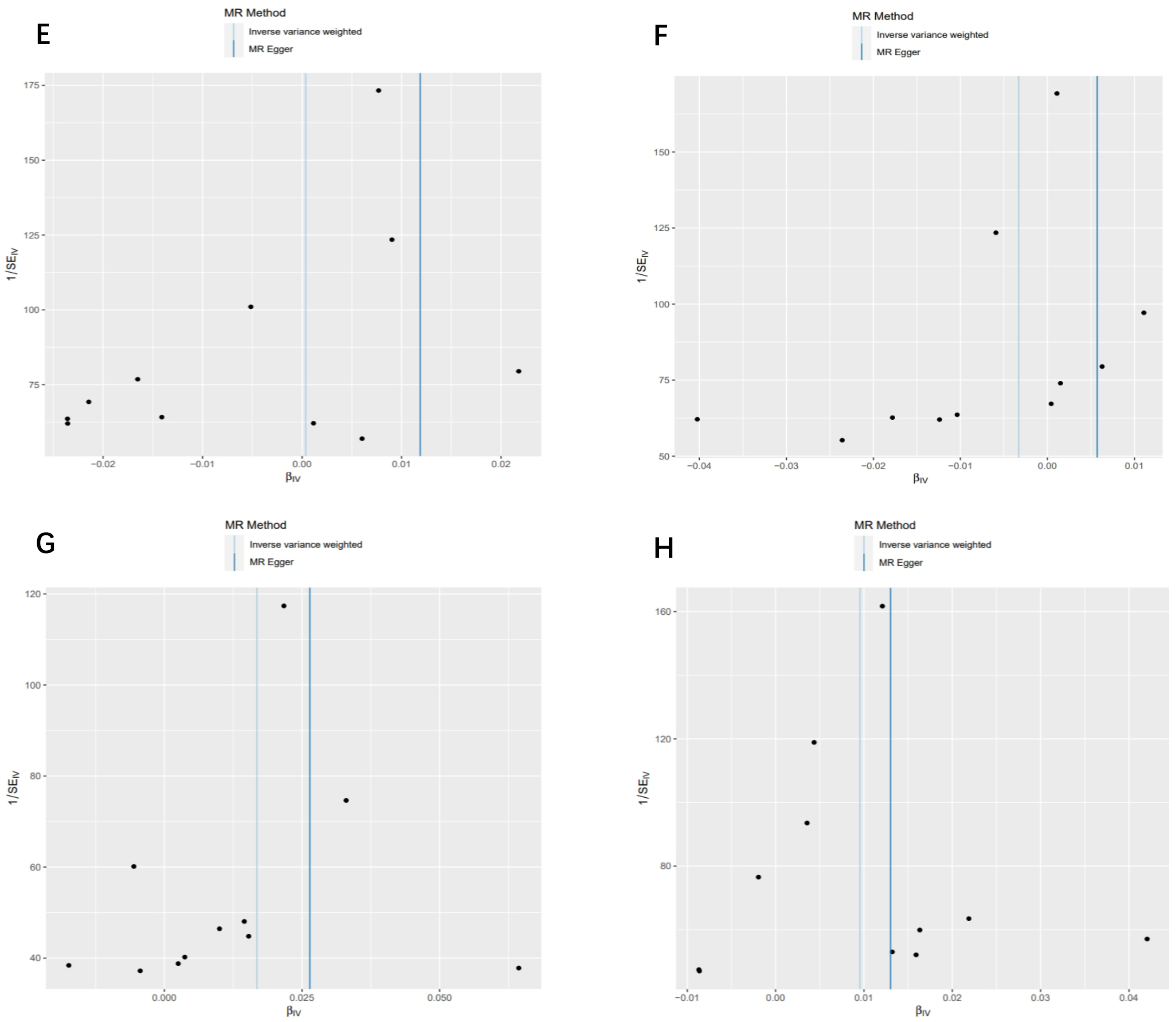
E
F
G
H
MR Method
Inverse variance weighted
MR Egger
$1/SE_{IV}$
β_{IV}
175
150
125
100
75
50
120
80
60
40
160
-0.04
-0.03
-0.02
-0.01
0.00
0.01
0.02
0.03
0.04
0.000
0.025
0.050

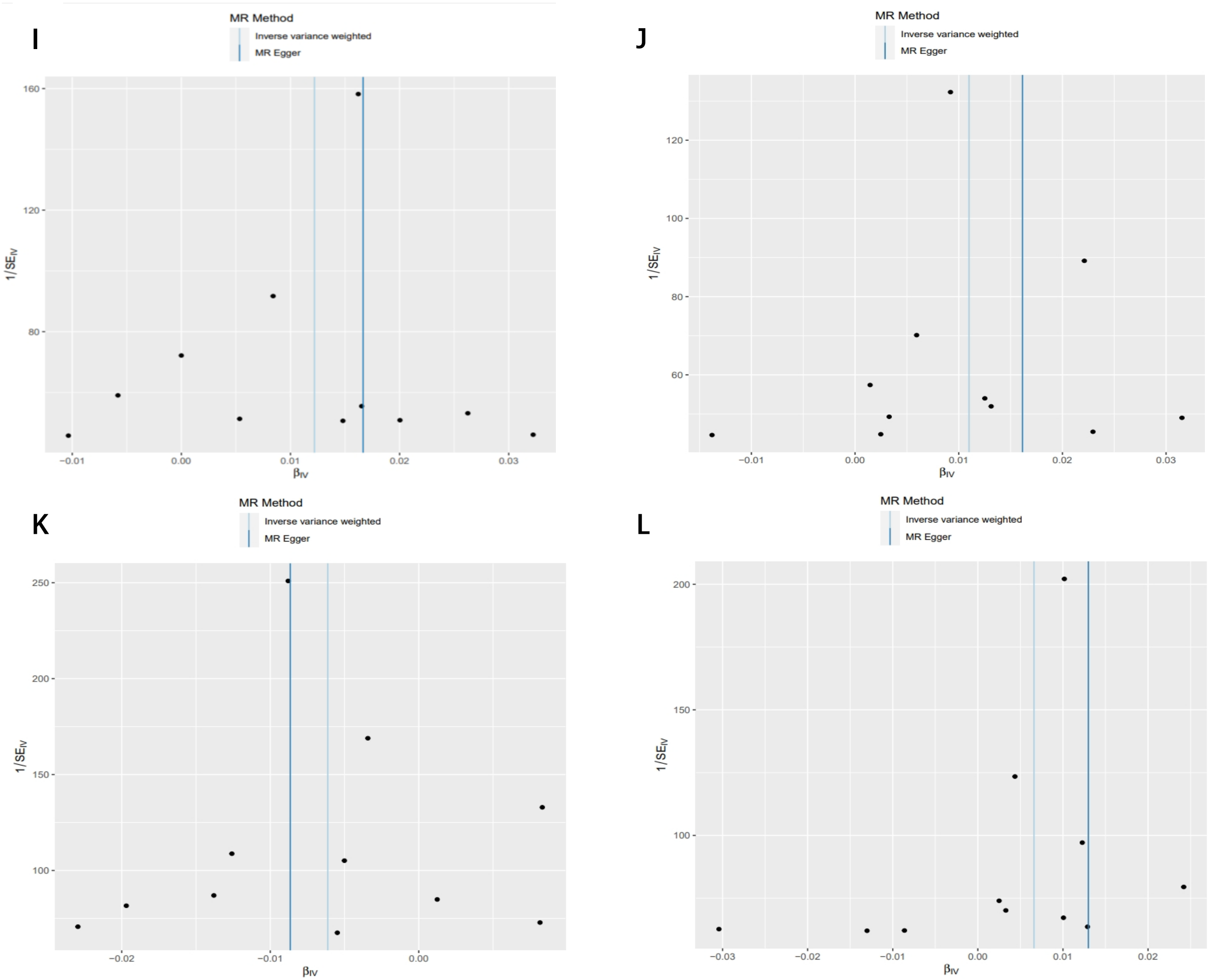
I
MR Method
Inverse variance weighted
MR Egger
160
120
80
1/SE_IV
-0.01
0.00
0.01
0.02
0.03
β_IV
J
MR Method
Inverse variance weighted
MR Egger
120
100
80
60
1/SE_IV
-0.01
0.00
0.01
0.02
0.03
β_IV
K
MR Method
Inverse variance weighted
MR Egger
250
200
150
100
1/SE_IV
-0.02
-0.01
0.00
β_IV
L
MR Method
Inverse variance weighted
MR Egger
200
150
100
1/SE_IV
-0.03
-0.02
-0.01
0.00
0.01
0.02
β_IV

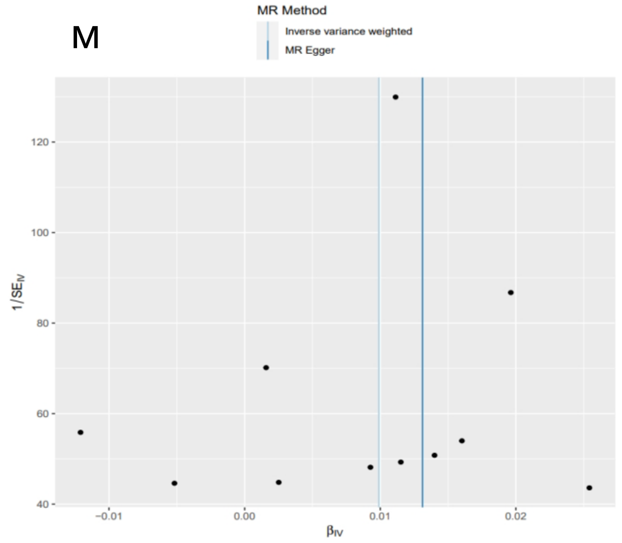

Figure S19 Funnel plots for the Graves' disease on thirteen potential metabolites. (A) Glutamine, (B) Stearate (18:0), (C) 2-hydroxystearate, (D) 1-oleoylglycerol (1-monoolein), (E) 2-hydroxyisobutyrate, (F) 3-(4-hydroxyphenyl)lactate, (G) Scyllo-inositol, (H) 1-arachidonoylglycerophosphocholine*, (I) 1-palmitoleoylglycerophosphocholine*, (J) 1-docosahexaenoylglycerophosphocholine*, (K) N2,N2-dimethylguanosine, (L) Glutaroyl carnitine, (M) 1-myristoylglycerophosphocholine

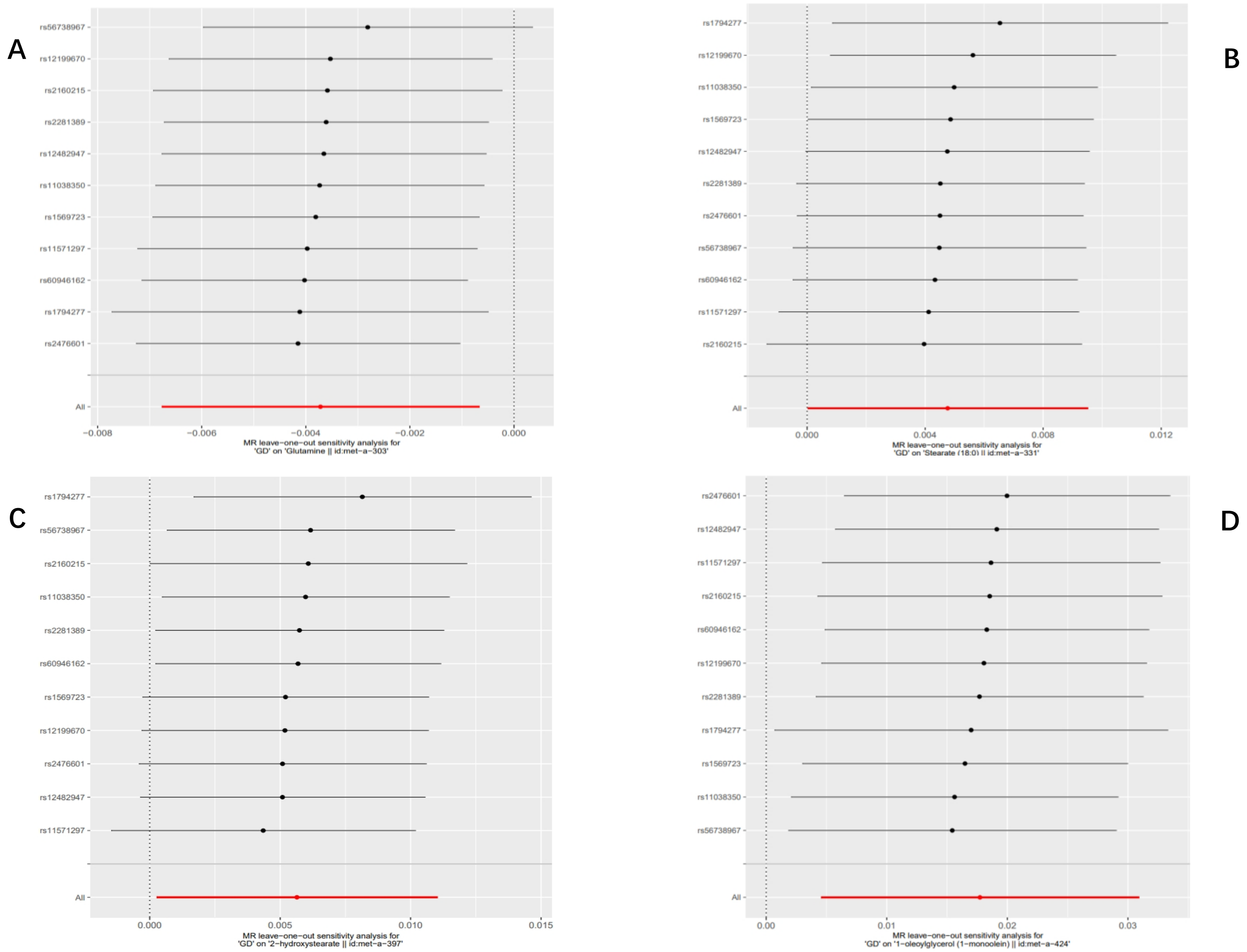
A
rs56738967
rs12199670
rs2160215
rs2281389
rs12482947
rs11038350
rs1569723
rs11571297
rs60946162
rs1794277
rs2476601
All
−0.008
−0.006
−0.004
−0.002
0.000
MR leave−one−out sensitivity analysis for
'GD' on 'Glutamine || id:met−a−303'
B
rs1794277
rs12199670
rs11038350
rs1569723
rs12482947
rs2281389
rs2476601
rs56738967
rs60946162
rs11571297
rs2160215
All
0.000
0.004
0.008
0.012
MR leave−one−out sensitivity analysis for
'GD' on 'Stearate (18:0) || id:met−a−331'
C
rs1794277
rs56738967
rs2160215
rs11038350
rs2281389
rs60946162
rs1569723
rs12199670
rs2476601
rs12482947
rs11571297
All
0.000
0.005
0.010
0.015
MR leave−one−out sensitivity analysis for
'GD' on '2−hydroxystearate || id:met−a−397'
D
rs2476601
rs12482947
rs11571297
rs2160215
rs60946162
rs12199670
rs2281389
rs1794277
rs1569723
rs11038350
rs56738967
All
0.00
0.01
0.02
0.03
MR leave−one−out sensitivity analysis for
'GD' on '1−oleoylglycerol (1−monoolein) || id:met−a−424'

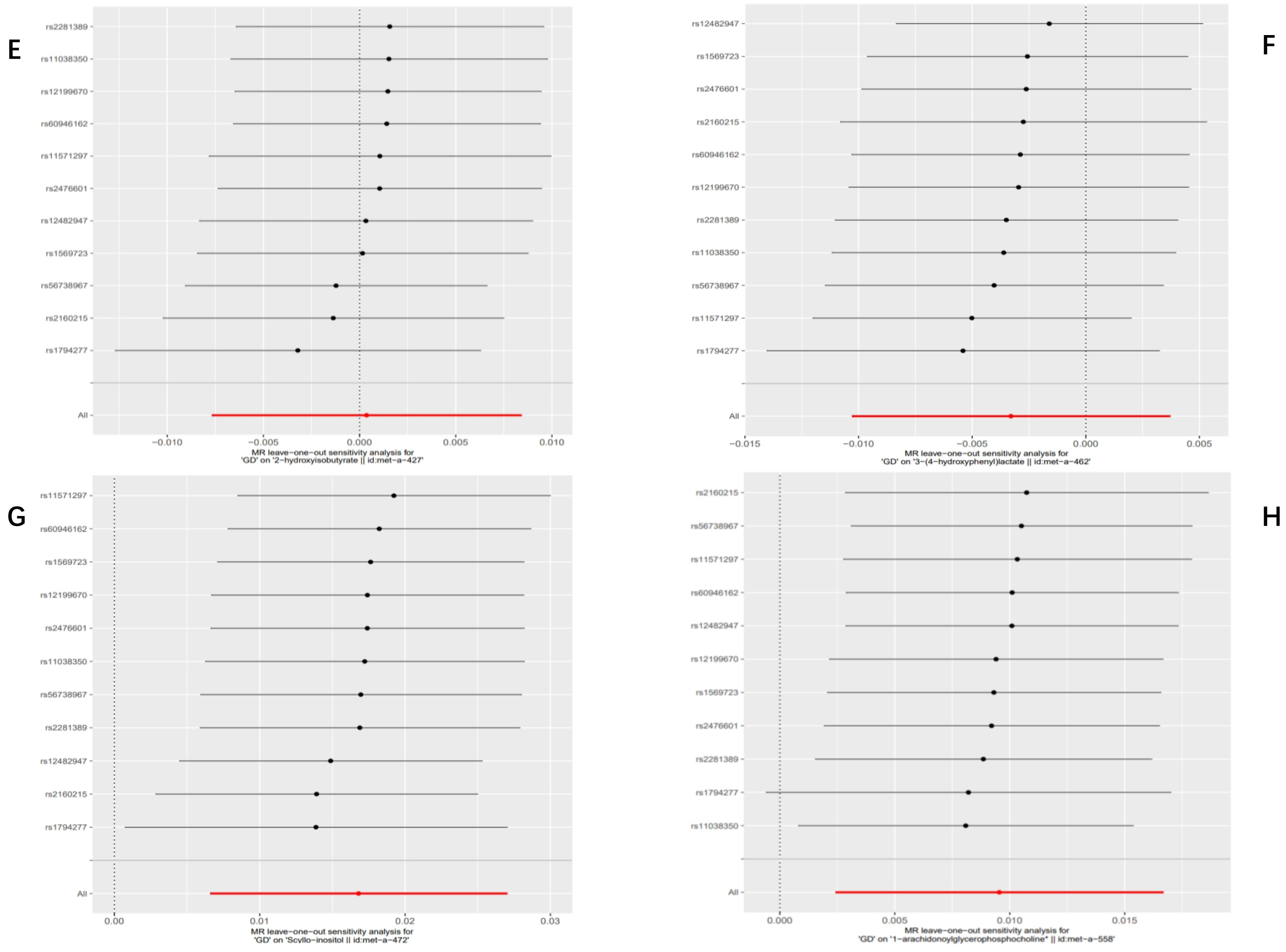
E
rs2281389
rs11038350
rs12199670
rs60946162
rs11571297
rs2476601
rs12482947
rs1569723
rs56738967
rs2160215
rs1794277
All
−0.010 −0.005 0.000 0.005 0.010
MR leave−one−out sensitivity analysis for
'GD' on '2−hydroxyisobutyrate || id:met−a−427'
F
rs12482947
rs1569723
rs2476601
rs2160215
rs60946162
rs12199670
rs2281389
rs11038350
rs56738967
rs11571297
rs1794277
All
−0.015 −0.010 −0.005 0.000 0.005
MR leave−one−out sensitivity analysis for
'GD' on '3−(4−hydroxyphenyl)lactate || id:met−a−462'
G
rs11571297
rs60946162
rs1569723
rs12199670
rs2476601
rs11038350
rs56738967
rs2281389
rs12482947
rs2160215
rs1794277
All
0.00 0.01 0.02 0.03
MR leave−one−out sensitivity analysis for
'GD' on 'Scyllo−inositol || id:met−a−472'
H
rs2160215
rs56738967
rs11571297
rs60946162
rs12482947
rs12199670
rs1569723
rs2476601
rs2281389
rs1794277
rs11038350
All
0.000 0.005 0.010 0.015
MR leave−one−out sensitivity analysis for
'GD' on '1−arachidonoylglycerophosphocholine* || id:met−a−558'

I

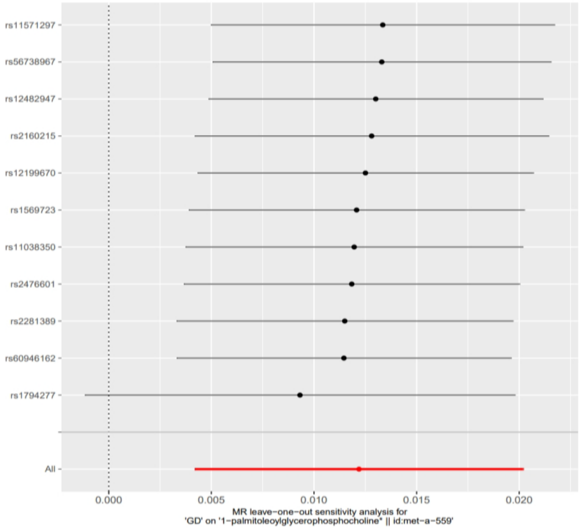
rs11571297
rs56738967
rs12482947
rs2160215
rs12199670
rs1569723
rs11038350
rs2476601
rs2281389
rs60946162
rs1794277
All
0.000
0.005
0.010
0.015
0.020
MR leave−one−out sensitivity analysis for
'GD' on '1−palmitoleoylglycerophosphocholine* || id:met−a−559'

J

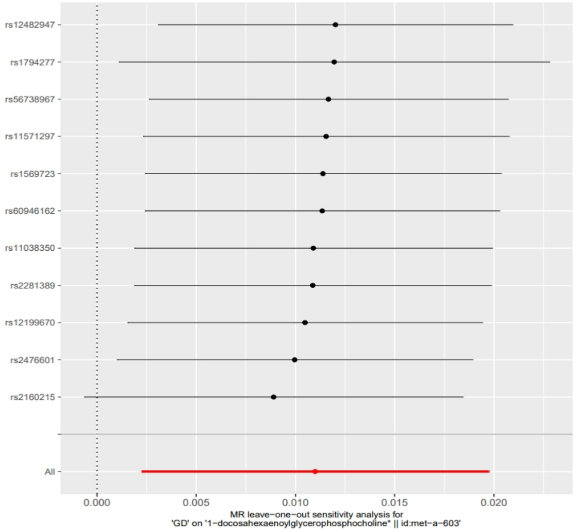
rs12482947
rs1794277
rs56738967
rs11571297
rs1569723
rs60946162
rs11038350
rs2281389
rs12199670
rs2476601
rs2160215
All
0.000
0.005
0.010
0.015
0.020
MR leave−one−out sensitivity analysis for
'GD' on '1−docosahexaenoylglycerophosphocholine* || id:met−a−603'

K

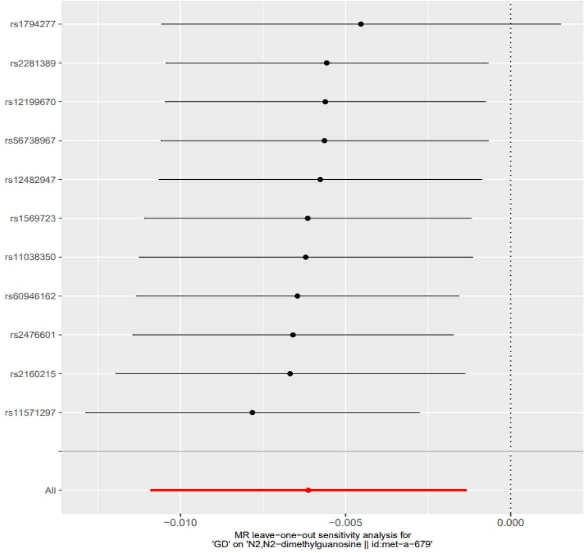
rs1794277
rs2281389
rs12199670
rs56738967
rs12482947
rs1569723
rs11038350
rs60946162
rs2476601
rs2160215
rs11571297
All
−0.010
−0.005
0.000
MR leave−one−out sensitivity analysis for
'GD' on 'N2,N2−dimethylguanosine || id:met−a−679'

L

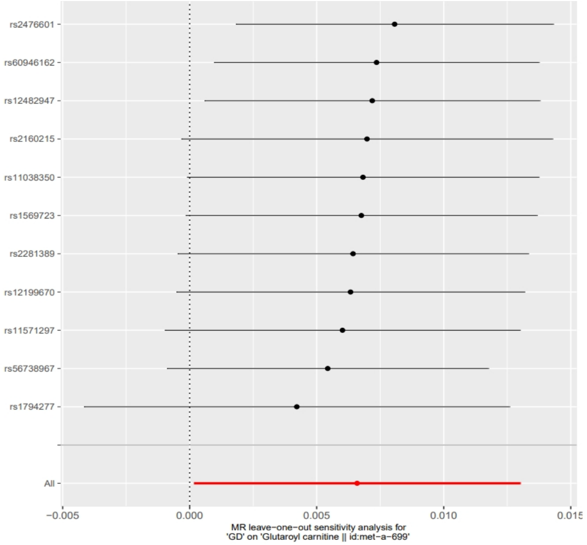
rs2476601
rs60946162
rs12482947
rs2160215
rs11038350
rs1569723
rs2281389
rs12199670
rs11571297
rs56738967
rs1794277
All
−0.005
0.000
0.005
0.010
MR leave−one−out sensitivity analysis for
'GD' on 'Glutaroyl carnitine || id:met−a−699'

M

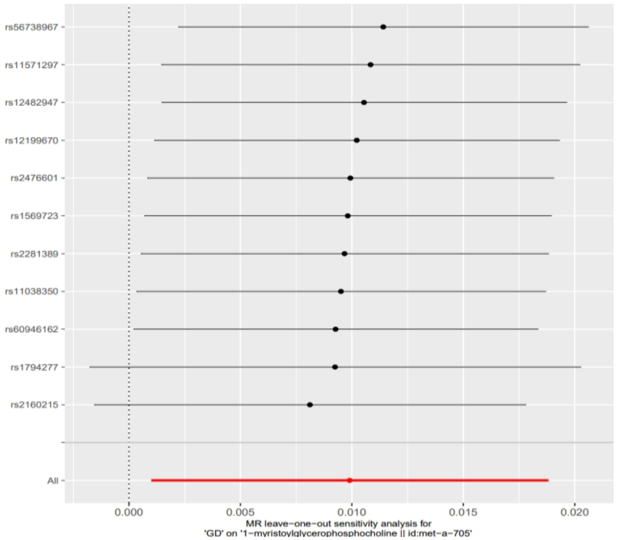

Figure S20 Leave-one-out plots for the Graves' disease on thirteen potential metabolites. (A) Glutamine, (B) Stearate (18:0), (C) 2-hydroxystearate, (D) 1-oleoylglycerol (1-monoolein), (E) 2-hydroxyisobutyrate, (F) 3-(4-hydroxyphenyl)lactate, (G) Scyllo-inositol, (H) 1-arachidonoylglycerophosphocholine*, (I) 1-palmitoleoylglycerophosphocholine*, (J) 1-docosahexaenoylglycerophosphocholine*, (K) N2,N2-dimethylguanosine, (L) Glutaroyl carnitine, (M) 1-myristoylglycerophosphocholine
